# Extended Discrete-Time Population Model to Describe the Competition of Nutrient-Producing Protocells

**DOI:** 10.1007/s11538-025-01488-0

**Published:** 2025-07-18

**Authors:** Richárd Kicsiny, Tamás Bódai, László Székely, Zoltán Varga

**Affiliations:** 1 Department of Mathematics and Modelling, Institute of Mathematics and Basic Science, Hungarian University of Agriculture and Life Sciences, Páter K. u. 1., Gödöllő, 2100 Hungary; 2 Department of Applied Statistics, Institute of Mathematics and Basic Science, Hungarian University of Agriculture and Life Sciences, Páter K. u. 1., Gödöllő, 2100 Hungary

**Keywords:** Nutrient-producing protocells, Discrete-time dynamic population model, Computer experiments, Anomaly on survival, Artificial life

## Abstract

Modeling the behavior of simple communities of *protocells* (as basic life-like organisms) is of vital importance since their better understanding may help to describe more complex (artificial and real) ecological systems. In this paper, we extend a recently developed *discrete-time* dynamic population model (called *preliminary model*) to a more general, completely reformulated version for describing the competition in a community of three protocell species (one *generalist* and two *specialists*). The advantage for the generalist is that it produces more kinds of nutrients than the specialists. In contrast to the preliminary model, the reproduction times and the times of (first) appearance of the three species can be all different in the *extended model*. The aim is to achieve the most basic/fundamental model that already displays complex population phenomena, like competitive exclusion, keystone species and an interesting “anomaly” regarding the connection between the survival of certain species and the decreasing rates of certain nutrients in the environment. Although, we could achieve this aim with a three-species model, at the simplest level, the model can be easily extended for more species in the future. The mentioned “anomaly” is a new discovery as it was not observed in the preliminary model. A particular equilibrium, when only the generalist survives, is exactly analyzed, where, interestingly, the golden ratio arises regarding the densities of the protocells of different ages. In future works, the extended model may serve as a useful tool for studying further phenomena in ecosystems, in their pure/abstract form.

**Supplementary Information:**

The online version contains supplementary material available at 10.1007/s11538-025-01488-0.

## Introduction

Modeling *protocells’* behavior increasingly comprehensively and accurately, from the genetic level to the level of populations, is a basic scientific issue. (In the present paper, the concept of protocells is applied as the most fundamental type of artificial living organisms. In general terms, it corresponds to the concept of cells, as used in biology). Since one can consider the multi-protocell communities as the most basic several-species systems, their better description may serve as an appropriate starting point to understand and model the processes in more complex artificial living and real biological systems more accurately.

Accordingly, in the present paper, we extend a recently developed *discrete-time* dynamic population model (Kicsiny et al. 2025) (called *preliminary model*) to a more general version (called *extended model*), by means of complete reformulation, for describing the competition in a community of three nutrient-producing protocell species (one *generalist* and two *specialists*). The model describes the time development of the densities (quantities) of the protocells and the nutrients.

In fact, we established the preliminary population model on the basis of modeling the protocells with Turing machines (Turing 1936) at the genetic level, nevertheless, this basis can be disregarded at the population level, and the extended mathematical model can be applied to ecosystems of protocells of arbitrary origins.

In some sense, the extended model is aimed to be the most simple/basic one that can already produce complex dynamic phenomena of ecosystems. Such phenomena, observed in computer experiments, are the competitive exclusion (Gause 1932; Hardin 1960), the effect of keystone species (Paine 1969; 1995) and a certain “anomaly” discussed in Sect. 3.1. We could achieve this simplicity with a system of not more than three species (however, the model can be easily extended to systems of more species in future works). The mentioned “anomaly” is a novel discovery of the extended model as it was not observed in the simulations of the preliminary model.

It should be mentioned that the direct subject of our study is *artificial life* (*ALife*), by means of the concept of protocells itself, nevertheless, the consequences regarding natural/biological life are clear as, for example, the mentioned phenomena (competitive exclusion etc.), observed in the extended model, are life-like.

ALife deals with the analysis of systems with respect to natural life processes and their evolution, in terms of computer model simulations, robotics and biochemistry. The term of “artificial life” was introduced by the biologist Christopher G. Langton in 1986 (see (Wilson, 1999)). According to the classification defined by Bedau (2003), there are three types of ALife: A) soft (derived from software), B) hard (derived from hardware) and C) wet (derived from biochemistry), see also (Gershenson et al. 2020). For an overview of category A), (Komosinski and Adamatzky 2009) can be a reference. Category B), involving robotics is reviewed in (Adamatzky and Komosinski 2009). Finally, for category C), (Dittrich et al. 2001) is a general review. As one of the most important concepts in the latter category, Tibor Gánti’s abstract model, the chemoton (Gánti et al., 2003), as a kind of protocell, is to be mentioned. The subject of the present paper falls in category A) using a discrete-time dynamics in modeling population/competition processes of protocells.

The proposed (extended) model is of discrete-time, nevertheless, its closest and most competent alternative might be the classical, competitive, basically *continuous-time*, Lotka-Volterra model (Duan et al. 2023; Scudo and Ziegler 1978; Volterra 1931), more specifically its following three-species version:

$$\frac{{dQ_{1} \left( t \right)}}{dt} = r_{1} Q_{1} \left( t \right)\left( {1 - \frac{{Q_{1} \left( t \right) + \alpha_{1,2} Q_{2} \left( t \right) + \alpha_{1,3} Q_{3} \left( t \right)}}{{K_{1} }}} \right),$$

$$\frac{{dQ_{2} \left( t \right)}}{dt} = r_{2} Q_{2} \left( t \right)\left( {1 - \frac{{Q_{2} \left( t \right) + \alpha_{2,1} Q_{1} \left( t \right) + \alpha_{2,3} Q_{3} \left( t \right)}}{{K_{2} }}} \right),$$

$$\frac{{dQ_{3} \left( t \right)}}{dt} = r_{3} Q_{3} \left( t \right)\left( {1 - \frac{{Q_{3} \left( t \right) + \alpha_{3,1} Q_{1} \left( t \right) + \alpha_{3,2} Q_{2} \left( t \right)}}{{K_{3} }}} \right),$$

where ${Q}_{1}\left(t\right)$, ${Q}_{2}\left(t\right)$ and ${Q}_{3}\left(t\right)$ are the species’ densities and ${K}_{1}$, ${K}_{2}$, ${K}_{3}$, ${r}_{1}$, ${r}_{2}$, ${r}_{3}$, ${\alpha }_{\text{1,2}}$, ${\alpha }_{\text{1,3}}$, ${\alpha }_{\text{2,1}}$, ${\alpha }_{\text{2,3}}$, ${\alpha }_{\text{3,1}}$, ${\alpha }_{\text{3,2}}$ are constant parameters. Discrete-time predator–prey models were studied e.g. in (Streipert et al. 2022; Weide et al. 2019). In (Gámez et al. 2017) systems-theoretical study was carried out for more general (but continuous-time) population systems.

Compared to the above Lotka-Volterra model, our population model differs in several aspects, namely:

- In the extended model, the species’ reproduction times can be different.
- The different species are able to produce different nutrients that are necessary for themselves and for the other species of the community. (Basically, the advantage for the generalist is that it can produce more kinds of nutrients than the specialists.)
- The model can consider the nutrient accumulating and decaying effects of the environment. In other words, both the case when some effect (e.g. some chemical reaction) increases and the case when some other effect (e.g. some radiation or chemical reaction) reduces the densities of some of the nutrients (denoted by $x$, ${x}_{1}$ and ${x}_{2}$) available in the environment.
- The model directly counts with the limited lifetime and the current age of the protocells.
- In addition to the above, if the amounts of the nutrients are scarce for all demands, the model directly considers the probabilistic feature of the sharing of the nutrients by the protocells, following a uniform distribution.

We note that in the extended model different time constraints will play a key role. Recently, in the dynamic ecological modeling of the fine details, time constraints represent an important issue, see (Garay et al. 2015). Indeed, in predator–prey models, time constraints derived from the activity distribution with respect to searching and handling, definitely determine the dynamic system properties, such as stable coexistence. A particular time constraint is time delay considered e.g. in (Din 2021) and (Din et al. 2021), dealing with models in epidemiology. In dynamic modeling, uncertainties are mostly dealt with stochastic versions, see e.g. (Din 2021; Din et al. 2021; Din and Li 2021; tul Ain, 2024). In continuous-time dynamic models the application of fractional order derivatives is a recent development, see (Ullah 2024; Khan et al. 2024).

Summing up, while the above mentioned Lotka-Volterra system is a *black-box model*, which means that it ignores the internal structure within a complex system, our approach results in a *white-box model*. The latter means that it is *mechanism-based*, involving the fine details of the interactions. A methodological discussion on the advantages and drawbacks of black-box, white-box and intermediate grey-box models can be found in (Loyola-Gonzalez 2019).

While the well-known matrix model of Leslie (1945) considers a population’s limited lifetime and age distribution, it describes the dynamics of a single species, which constitutes the main difference compared to our (several-species) model. Another often used approach to modeling of population dynamics is evolutionary game dynamics (Escobar-Cuevas et al. 2024; Hofbauer and Sigmund 1988). In this approach, the densities of the different phenotypes (mutants) evolve in time determined by the difference between their fitness values and the average fitness value of the whole system (replicator dynamics). Population genetics is combined with evolutionary games e.g. in (Garay and Varga 1998). Under some linearity conditions, the densities converge to an asymptotically stable solution, which is determined by the so-called evolutionary stable strategies of the related evolutionary game model. In the extended model, however, the natural selection (Darwin 1859) operates not by means of heuristic fitness values but through that whether the protocells get the needed nutrients.

As for the practical applicability of the extended model, we mention that it may help to simulate the interactions between populations and may contribute to the better understanding of the efficiency of biological pest control (biocontrol), an important issue of *bio-* and *agricultural engineering*. For the discussion of different technics of biocontrol (e.g. such as inoculative and inundative releases) (Heimpel and Mills 2017) is a recent monograph.

Summing up, the main contributions of the present paper are the following:

1. A recent discrete-time dynamic population model (preliminary model) for a generalist and two specialist protocell species is extended to a more general model (extended model). In contrast to the preliminary model, the reproduction times of all three species and their times of (first) appearance in the ecosystem can be different in the extended model. (In the preliminary model, the specialists’ reproduction times were fixed to be equal, and each species started its operation at the same (initial) modeling time point.)
2. Among other complex dynamic phenomena of ecosystems (like the competitive exclusion or the effect of keystone species), the extended model is able to produce an interesting “anomaly” regarding the connection between the survival of certain species and the rates of decrease of certain nutrients. This “anomaly” is new in the sense that it was not observed in the preliminary model.

The main importance of the present work is that it realizes a kind of most basic possible model that already displays complex and interesting population dynamics phenomena. The property of being “basic” implies here that the governing mathematical relations of the model themselves are simple algebraic ones and that there are only three species (with possibly different reproduction times) in the modelled system. Because of this simplicity, we think that in future works, the present extended model will serve as a simple and useful tool for studying more life-like phenomena (e.g. selection and evolution) in ecosystems, in their most direct mathematical/algorithmic form (similarly as in (Feverati and Musso 2008; Musso and Feverati 2012)).

The paper is organized as follows: In Sect. 2, the extended model is presented. Section 3 discusses the results produced by the model in computer experiments and their practical interpretation. Furthermore, the exact mathematical analysis of a particular equilibrium of the protocell species is provided in Sect. 3. Final conclusions and future research lines are given in Sect. 4.

## Extended Population Model

In this section, the extended population model describing the competition for the nutrients in a common habitat/environment among three protocell species (a generalist denoted by ${T}_{0}$ and two specialists denoted by ${T}_{1}$ and ${T}_{2}$) is presented. This dynamic model (formulated in Sect. 2.2) describes the densities of the protocells and the densities of the nutrients as functions of time. The extended model is an extended/generalized, and completely reformulated, version of the preliminary model.

Briefly speaking, the main operation of the extended model (which could be enough for a satisfactory understanding of the results of the paper) is as follows: At the current modeling time point, (only) the active protocells (for which the current modeling time point is an operation time point as well) compete for the nutrients available in the environment. Their access to the nutrients depends on the densities of themselves and of the nutrients according to probabilistic rules. If an active protocell gets all required nutrients, it survives producing an offspring and certain nutrient(s), otherwise it dies without producing any offspring and nutrient. Then the next modeling time point follows. Under different conditions in the environment and different parameter values of the model, diverse developments of the densities can be observed by means of the extended model (in computer experiments).

### Main Features Determining the Model

The main features determining the extended model, which come partly from the features of the preliminary model (Kicsiny et al. 2025), are the following:

- The reproduction times of the generalist (${\tau }_{0}$) and the two specialists (${\tau }_{1}$ for ${T}_{1}$ and ${\tau }_{2}$ for ${T}_{2}$) can be different (${\tau }_{0}\ne {\tau }_{1}$, ${\tau }_{0}\ne {\tau }_{2}$, ${\tau }_{1}\ne {\tau }_{2}$ are possible), as well as their initial operation time points can be different—in contrast to the preliminary model, where ${\tau }_{1}={\tau }_{2}$, and all species start its operation at the same (first) modeling time point.
- At the beginning of a given reproductive cycle of a protocell, it requires all three types of nutrients $x$, ${x}_{1}$ and ${x}_{2}$, 1 unit of each, for its normal functioning, including the production of a single offspring (per cycle) and some nutrients. The protocell survives if this requirement about the nutrients is satisfied, otherwise, the protocell does not survive at the current cycle of itself.
- A protocell’s lifetime is at most 2 cycles, which means $2{\tau }_{0}$ time for the generalist, $2{\tau }_{1}$ time for specialist ${T}_{1}$ and $2{\tau }_{2}$ time for specialist ${T}_{2}$. Let us call a protocell at the beginning of its first cycle “young”, and let us call a protocell at the beginning of its second cycle “old”. In accordance with it, a protocell is able to produce at most 2 offspring in its life. (Generally speaking, 2 offspring are needed minimally in order that the overall density of the protocells is able to increase—under proper conditions.) This happens if the protocell gets 1 unit of $x$, ${x}_{1}$ and ${x}_{2}$ at the beginning of its both cycles. If any of the needed nutrients is missing, the protocell dies (does not survive) without producing any offspring at its current cycle, furthermore, it releases the already absorbed nutrients (if any) to the environment (by the next modeling time point of the model). In fact, the model takes the protocells’ current age and limited lifetime into account.
- The different species are able to produce different kinds of nutrients. More precisely, in case of survival, ${T}_{0}$ produces two units of both ${x}_{1}$ and ${x}_{2}$ (in sum, four units of nutrients), ${T}_{1}$ produces two units of only ${x}_{1}$ and ${T}_{2}$ produces two units of only ${x}_{2}$ at its current cycle. Furthermore, whatever protocell, if it survives at its current cycle, it passes half of the produced nutrients to its offspring directly and the other half to the environment. Particularly, ${T}_{0}$ passes 1 unit of ${x}_{1}$ and 1 unit of ${x}_{2}$ both to its offspring and to the environment, while ${T}_{1}$ passes 1 unit of ${x}_{1}$ to its offspring and the same to the environment and ${T}_{2}$ passes 1 unit of ${x}_{2}$ to its offspring and the same to the environment.
- The model can describe either the nutrient accumulation or the nutrient decaying effect of the environment, that is, either when some effect (e.g. some chemical reaction) increases or when some effect (e.g. some chemical reaction or radiation) decreases the available amount of nutrients $x$, ${x}_{1}$ and ${x}_{2}$ in the environment. In the mathematical model below, the rates of increase/decrease of $x$, ${x}_{1}$ and ${x}_{2}$ in the environment are expressed by the positive/negative values of the parameters $b$, ${b}_{1}$ and ${b}_{2}$, respectively. (Therefore, ${x}_{1}$ and ${x}_{2}$ can be produced both by the protocells and in the environment, independently of the protocells, while $x$ can be produced only in the environment.)
- Finally, the probabilistic character of the equal/uniform share that the protocells have regarding the nutrients in the environment is also described by the model according to a (discrete) uniform distribution, in case of that the densities of the nutrients in the environment are not enough for all needs.

Before formulating the discrete-time model mathematically, the modeling time points of the model (${t}_{0}$, ${t}_{1}$, ${t}_{2}$, …) and the operation time points of the protocells (${h}_{{\uptau }_{0}}\left(0\right)$, ${h}_{{\uptau }_{0}}\left(1\right)$, ${h}_{{\uptau }_{0}}\left(2\right)$, … for ${T}_{0}$; ${h}_{{\uptau }_{1}}\left(0\right)$, ${h}_{{\uptau }_{1}}\left(1\right)$, ${h}_{{\uptau }_{1}}\left(2\right)$, … for ${T}_{1}$ and ${h}_{{\uptau }_{2}}\left(0\right)$, ${h}_{{\uptau }_{2}}\left(1\right)$, ${h}_{{\uptau }_{2}}\left(2\right)$, … for ${T}_{2}$) should be defined. In contrast to the preliminary model, the different protocell species can appear more generally, at different times, in the ecosystem in the extended model. It is expressed by the following symbols:

${t}_{\text{0,0}}$: initial operation time point of the generalist (${T}_{0}$),

${t}_{\text{1,0}}$: initial operation time point of the “first” specialist (${T}_{1}$),

${t}_{\text{2,0}}$: initial operation time point of the “second” specialist (${T}_{2}$).

It is required that at least on of ${t}_{\text{0,0}}$, ${t}_{\text{1,0}}$ and ${t}_{\text{2,0}}$ is 0 (initial modeling time point of the ecosystem).

The set of the (all) modeling time points (of the ecosystem) is defined as

$$\begin{aligned} T & := \left\{ {t_{0,0} ; t_{0,0} + \tau_{0} ;{ }t_{0,0} + 2\tau_{0} ; \ldots } \right\} \cup \left\{ {t_{1,0} ; t_{1,0} + \tau_{1} ;{ }t_{1,0} + 2\tau_{1} ; \ldots } \right\}\\ &\quad \cup \left\{ {t_{2,0} ; t_{2,0} + \tau_{2} ;{ }t_{2,0} + 2\tau_{2} ; \ldots } \right\}. \end{aligned}$$

Let the (modeling) time points ${t}_{\text{i}}$ $\left(i=0, 1, 2,\dots \right)$ be the elements of $T$ in increasing order (${t}_{0}:=0<{t}_{1}<{t}_{2}<\dots$).

Then it holds for a unique index ${i}_{0}$ that ${t}_{{\text{i}}_{0}}={t}_{\text{0,0}}$, for a unique index ${i}_{1}$ that ${t}_{{\text{i}}_{1}}={t}_{\text{1,0}}$ and for a unique index ${i}_{2}$ that ${t}_{{\text{i}}_{2}}={t}_{\text{2,0}}$. (In addition, $\text{min}\left({i}_{0}, {i}_{1}, {i}_{2}\right)=0$.)

**Fig. 1:**
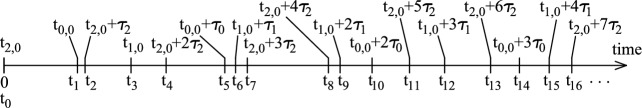
Modeling time points according to the (possibly different) reproduction times of the three protocell species

Figure 1 represents the modeling time points of the model, generally.

Let us define the sequences $\left( {h_{{{\uptau }_{0} }} \left( i \right)} \right)$, $\left( {h_{{{\uptau }_{1} }} \left( i \right)} \right)$ and $\left( {h_{{{\uptau }_{2} }} \left( i \right)} \right)$ as follows:

1. $$h_{{\tau_{0} }} \left( {i_{0} } \right):= t_{0,0}$$; $$h_{{\tau_{1} }} \left( {i_{1} } \right): = t_{1,0}$$; $$h_{{\tau_{2} }} \left( {i_{2} } \right): = t_{2,0}$$.
2. If $i < i_{0}$, then $h_{{{\uptau }_{0} }} \left( i \right)$ is not defined,
  if $$i > i_{0}$$, then $$h_{{{\uptau }_{0} }} \left( i \right): = \left\{ {\begin{array}{*{20}c} {t_{{\text{i}}} , if t_{{\text{i}}} = h_{{{\uptau }_{0} }} \left( {i - 1} \right) + {\uptau }_{0} ,} \\ {h_{{{\uptau }_{0} }} \left( {i - 1} \right), if t_{{\text{i}}} < h_{{{\uptau }_{0} }} \left( {i - 1} \right) + {\uptau }_{0} .} \\ \end{array} } \right.$$
3. If $i < i_{1}$, then $h_{{{\uptau }_{1} }} \left( i \right)$ not defined,
  if $$i > i_{1}$$, then $$h_{{{\uptau }_{1} }} \left( i \right): = \left\{ {\begin{array}{*{20}c} {t_{{\text{i}}} , if t_{{\text{i}}} = h_{{{\uptau }_{1} }} \left( {i - 1} \right) + {\uptau }_{1} ,} \\ {h_{{{\uptau }_{1} }} \left( {i - 1} \right), if t_{{\text{i}}} < h_{{{\uptau }_{1} }} \left( {i - 1} \right) + {\uptau }_{1} .} \\ \end{array} } \right.$$
4. If $i < i_{2}$, then $h_{{{\uptau }_{2} }} \left( i \right)$ not defined,
  if $$i > i_{2}$$, then $$h_{{{\uptau }_{2} }} \left( i \right): = \left\{ {\begin{array}{*{20}c} {t_{{\text{i}}} , if t_{{\text{i}}} = h_{{{\uptau }_{2} }} \left( {i - 1} \right) + {\uptau }_{2} ,} \\ {h_{{{\uptau }_{2} }} \left( {i - 1} \right), if t_{{\text{i}}} < h_{{{\uptau }_{2} }} \left( {i - 1} \right) + {\uptau }_{2} .} \\ \end{array} } \right.$$

#### Remark 2.1

${h}_{{\uptau }_{0}}\left(i\right)$, ${h}_{{\uptau }_{1}}\left(i\right)$ and ${h}_{{\uptau }_{2}}\left(i\right)$, if defined, stand essentially for the latest modeling time point $t\le {t}_{\text{i}}$ which is an operation time point of ${T}_{0}$, ${T}_{1}$ and ${T}_{2}$, respectively. Any modeling time point ${t}_{\text{i}}$ is an operation time point of ${T}_{0}$ if and only if ${t}_{\text{i}}={h}_{{\uptau }_{0}}\left(i\right)$. Otherwise, ${t}_{\text{i}}>{h}_{{\uptau }_{0}}\left(i\right)$, provided that ${h}_{{\uptau }_{0}}\left(i\right)$ is defined. Looking back from the current modeling time point ${t}_{\text{i}}$, ${h}_{{\uptau }_{0}}\left(i-1\right)$, if defined, is always the last, already passed ($<{t}_{\text{i}}$) operation time point of ${T}_{0}$. The functions ${h}_{{\uptau }_{1}}$ and ${h}_{{\uptau }_{2}}$ have similar meaning in relation to ${T}_{1}$ and ${T}_{2}$, respectively. (Some examples are the following in the particular case of Fig. 1: ${h}_{{\uptau }_{0}}\left(0\right)$ and ${h}_{{\uptau }_{1}}\left(0\right)$ are not defined, ${h}_{{\uptau }_{2}}\left(0\right)={t}_{\text{2,0}}={t}_{0}=0$, ${h}_{{\uptau }_{0}}\left(1\right)={t}_{\text{0,0}}={t}_{1}$, ${h}_{{\uptau }_{1}}\left(1\right)$ is not defined, ${h}_{{\uptau }_{2}}\left(1\right)={h}_{{\uptau }_{2}}\left(0\right)={t}_{0}=0$, ${h}_{{\uptau }_{0}}\left(2\right)={h}_{{\uptau }_{0}}\left(1\right)={t}_{1}$, ${h}_{{\uptau }_{1}}\left(2\right)$ is not defined, ${h}_{{\uptau }_{2}}\left(2\right)={t}_{2}$, ${h}_{{\uptau }_{0}}\left(3\right)={h}_{{\uptau }_{0}}\left(2\right)={t}_{1}$, ${h}_{{\uptau }_{1}}\left(3\right)={t}_{\text{1,0}}={t}_{3}$, ${h}_{{\uptau }_{2}}\left(3\right)={h}_{{\uptau }_{2}}\left(2\right)={t}_{2}$.)

In the below list, the main events of the population are summarized, according to Fig. 1, regarding the three types of protocells:

At times ${t}_{\text{0,0}}={t}_{1}$, ${t}_{\text{0,0}}+{\tau }_{0}={t}_{5}$, ${t}_{\text{0,0}}+{2\tau }_{0}={t}_{10}$, …: A young generalist, having (a unit of) ${x}_{1}$ and ${x}_{2}$ from its parent, tries to absorb (a unit of) $x$ from the environment. If it can absorb it, it produces an offspring supplied with (a unit of) ${x}_{1}$ and ${x}_{2}$ and releases (another unit of) ${x}_{1}$ and ${x}_{2}$ to the environment. After this, it becomes old. An old generalist tries to absorb (a unit of) $x$, ${x}_{1}$ and ${x}_{2}$ from the environment. If it can absorb them, it produces an offspring supplied with (a unit of) ${x}_{1}$ and ${x}_{2}$ and releases (another unit of) ${x}_{1}$ and ${x}_{2}$ to the environment. After this, it dies. If a generalist cannot absorb all needed nutrients, it dies and releases its already absorbed nutrient(s), if any, to the environment at the next modeling time point (${t}_{2}$, ${t}_{6}$, ${t}_{11}$, …).

At times ${t}_{\text{1,0}}={t}_{3}$, ${t}_{\text{1,0}}+{\tau }_{1}={t}_{6}$, ${t}_{\text{1,0}}+{2\tau }_{1}={t}_{9}$, …: A young ${T}_{1}$ specialist, having (a unit of) ${x}_{1}$ from its parent, tries to absorb (a unit of) $x$ and ${x}_{2}$ from the environment. If it can absorb them, it produces an offspring supplied with (a unit of) ${x}_{1}$ and releases (another unit of) ${x}_{1}$ to the environment. After this, it becomes old. An old ${T}_{1}$ specialist tries to absorb (a unit of) $x$, ${x}_{1}$ and ${x}_{2}$ from the environment. If it can absorb them, it produces an offspring supplied with (a unit of) ${x}_{1}$ and releases (another unit of) ${x}_{1}$ to the environment. After this, it dies. If a ${T}_{1}$ specialist cannot absorb all needed nutrients, it dies and releases its already absorbed nutrient(s), if any, to the environment at the next modeling time point (${t}_{4}$, ${t}_{7}$, ${t}_{10}$, …).

At times ${t}_{\text{2,0}}={t}_{0}$, ${t}_{\text{2,0}}+{\tau }_{2}={t}_{2}$, ${t}_{\text{2,0}}+{2\tau }_{2}={t}_{4}$, …: A young ${T}_{2}$ specialist, having (a unit of) ${x}_{2}$ from its parent, tries to absorb (a unit of) $x$ and ${x}_{1}$ from the environment. If it can absorb them, it produces an offspring supplied with (a unit of) ${x}_{2}$ and releases (another unit of) ${x}_{2}$ to the environment. After this, it becomes old. An old ${T}_{2}$ specialist tries to absorb (a unit of) $x$, ${x}_{1}$ and ${x}_{2}$ from the environment. If it can absorb them, it produces an offspring supplied with (a unit of) ${x}_{2}$ and releases (another unit of) ${x}_{2}$ to the environment. After this, it dies. If a ${T}_{2}$ specialist cannot absorb all needed nutrients, it dies and releases its already absorbed nutrient(s), if any, to the environment at the next modeling time point (${t}_{1}$, ${t}_{3}$, ${t}_{5}$, …).

### Mathematical Formulation of the Model

In this section, the exact mathematical formulation of the extended model is provided. Despite of that the differences between the particular cases that can be described by the extended model and the preliminary model seem relatively slight (compared to the numerous characteristic details of the models), the mathematical formulations of the two models are completely different. (As it was already mentioned, the extended model is more general than the preliminary model, regarding certain time parameters of the ecosystem.)

Let the young ${T}_{0}$-type protocell be denoted by ${T}_{\text{0,1}}$ and the old ${T}_{0}$-type protocell be denoted by ${T}_{\text{0,2}}$. In a similar way, let the young ${T}_{1}$-type protocell be denoted by ${T}_{\text{1,1}}$, the old ${T}_{1}$-type protocell be denoted by ${T}_{\text{1,2}}$, the young ${T}_{2}$-type protocell be denoted by ${T}_{\text{2,1}}$ and the old ${T}_{2}$-type protocell be denoted by ${T}_{\text{2,2}}$.

**Fig. 2:**
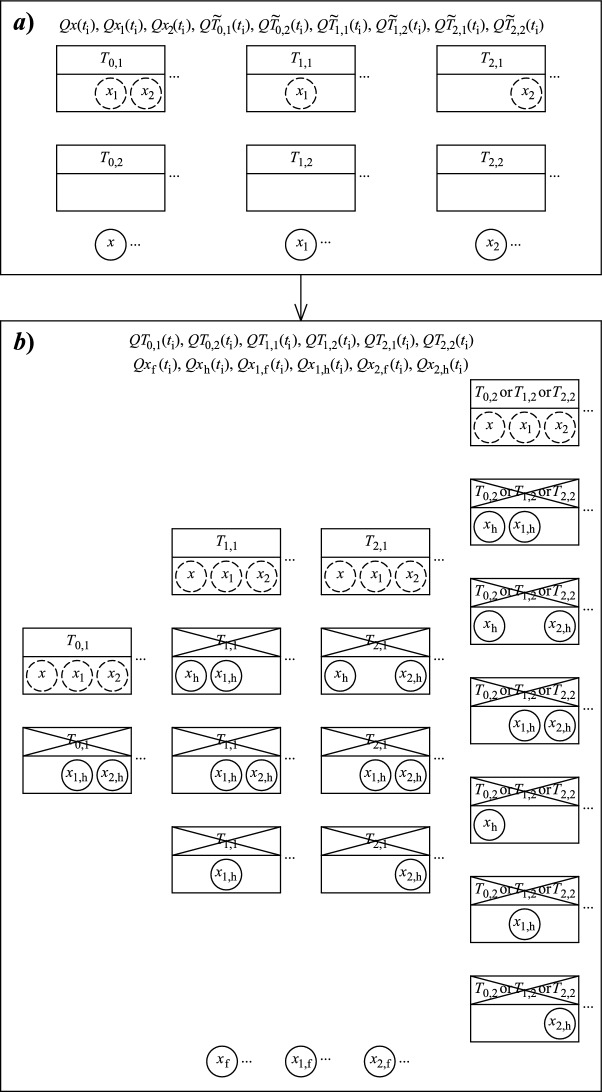
State variables of the model at modeling time point ${t}_{\text{i}}$ at two sub-steps ***a*** just before the competition and ***b*** just after the competition

Figure 2 presents the state variables of the discrete-time model at an arbitrary modeling time point ${t}_{\text{i}}$ $\left(i=0, 1, 2, \dots \right)$ at two different sub-steps: (*a*) just before the competition, that is, just before that the present protocells absorb the proper/available part of the present nutrients in the environment, according to their demands and the opportunities (see sub-figure *a*) in Fig. 2); (*b*) just after the competition, that is, just after that the present protocells absorb the proper/available part of the present nutrients in the environment, according to their demands and the opportunities (see sub-figure *b*) in Fig. 2). The competition is directed at getting the nutrients in the environment, which are possibly not enough for all protocells.

The state variables $Qx\left({t}_{\text{i}}\right)$, $Q{x}_{1}\left({t}_{\text{i}}\right)$ and $Q{x}_{2}\left({t}_{\text{i}}\right)$ are the densities of the three nutrients $x$, ${x}_{1}$ and ${x}_{2}$, respectively, at ${t}_{\text{i}}$, just before the competition. The state variables $Q{\widetilde{T}}_{\text{0,1}}\left({t}_{\text{i}}\right)$, $Q{\widetilde{T}}_{\text{0,2}}\left({t}_{\text{i}}\right)$, $Q{\widetilde{T}}_{\text{1,1}}\left({t}_{\text{i}}\right)$, $Q{\widetilde{T}}_{\text{1,2}}\left({t}_{\text{i}}\right)$, $Q{\widetilde{T}}_{\text{2,1}}\left({t}_{\text{i}}\right)$ and $Q{\widetilde{T}}_{\text{2,2}}\left({t}_{\text{i}}\right)$ are the densities of the protocells ${T}_{\text{0,1}}$, ${T}_{\text{0,2}}$, ${T}_{\text{1,1}}$, ${T}_{\text{1,2}}$, ${T}_{\text{2,1}}$ and ${T}_{\text{2,2}}$, respectively, at ${t}_{\text{i}}$, just before the competition as well.

The state variables $Q{T}_{\text{0,1}}\left({t}_{\text{i}}\right)$, $Q{T}_{\text{0,2}}\left({t}_{\text{i}}\right)$, $Q{T}_{\text{1,1}}\left({t}_{\text{i}}\right)$, $Q{T}_{\text{1,2}}\left({t}_{\text{i}}\right)$, $Q{T}_{\text{2,1}}\left({t}_{\text{i}}\right)$ and $Q{T}_{\text{2,2}}\left({t}_{\text{i}}\right)$ are the densities of the protocells ${T}_{\text{0,1}}$, ${T}_{\text{0,2}}$, ${T}_{\text{1,1}}$, ${T}_{\text{1,2}}$, ${T}_{\text{2,1}}$ and ${T}_{\text{2,2}}$, respectively, at ${t}_{\text{i}}$, just after the competition. Since these are the densities of the protocells surviving the competition at modeling time point ${t}_{\text{i}}$, naturally, $Q{T}_{\text{0,1}}\left({t}_{\text{i}}\right)\le Q{\widetilde{T}}_{\text{0,1}}\left({t}_{\text{i}}\right)$, $Q{T}_{\text{0,2}}\left({t}_{\text{i}}\right)\le Q{\widetilde{T}}_{\text{0,2}}\left({t}_{\text{i}}\right)$, $Q{T}_{\text{1,1}}\left({t}_{\text{i}}\right)\le Q{\widetilde{T}}_{\text{1,1}}\left({t}_{\text{i}}\right)$, $Q{T}_{\text{1,2}}\left({t}_{\text{i}}\right)\le Q{\widetilde{T}}_{\text{1,2}}\left({t}_{\text{i}}\right)$, $Q{T}_{\text{2,1}}\left({t}_{\text{i}}\right)\le Q{\widetilde{T}}_{\text{2,1}}\left({t}_{\text{i}}\right)$, $Q{T}_{\text{2,2}}\left({t}_{\text{i}}\right)\le Q{\widetilde{T}}_{\text{2,2}}\left({t}_{\text{i}}\right)$ hold.

If the density of any of the nutrients is not enough to meet the demands of all protocells before the competition (that is, if either $Qx\left({t}_{\text{i}}\right)$ or $Q{x}_{1}\left({t}_{\text{i}}\right)$ or $Q{x}_{2}\left({t}_{\text{i}}\right)$ is not enough for all protocells with densities ${\widetilde{T}}_{\text{0,1}}\left({t}_{\text{i}}\right)$, $Q{\widetilde{T}}_{\text{0,2}}\left({t}_{\text{i}}\right)$, $Q{\widetilde{T}}_{\text{1,1}}\left({t}_{\text{i}}\right)$, $Q{\widetilde{T}}_{\text{1,2}}\left({t}_{\text{i}}\right)$, $Q{\widetilde{T}}_{\text{2,1}}\left({t}_{\text{i}}\right)$ and $Q{\widetilde{T}}_{\text{2,2}}\left({t}_{\text{i}}\right)$), then the requiring protocells share the available amount of these nutrients in the competition, in accordance with (the probabilities of) discrete uniform distribution. This distribution fits the protocells’ (supposed) equal chances to get a (still free) unit of the nutrients (in the environment). If a protocell cannot get 1 unit of at least one of the required nutrients, it cannot operate, it does not survive the competition, and the units of nutrients that it may have already absorbed (from its parent or the environment) remain untouched by it and get out to the environment. There, the other protocells are able to utilize them at the (beginning of the) next modeling time point ${t}_{\text{i}+1}$. Let the total amount of such hidden nutrients—which are not utilized at the current modeling time point ${t}_{\text{i}}$ but released at the next modeling time point ${t}_{\text{i}+1}$—be denoted by $Q{x}_{\text{h}}\left({t}_{\text{i}}\right)$, $Q{x}_{1,\text{h}}\left({t}_{\text{i}}\right)$ and $Q{x}_{2,\text{h}}\left({t}_{\text{i}}\right)$ with respect to nutrients $x$, ${x}_{1}$ and ${x}_{2}$, respectively.

At the same time (at sub-step *b* of modeling time point ${t}_{\text{i}}$), let the nutrient densities that are not absorbed in the environment by the protocells (as there may be more from the given nutrients than required) be denoted by $Q{x}_{\text{f}}\left({t}_{\text{i}}\right)$, $Q{x}_{1,\text{f}}\left({t}_{\text{i}}\right)$ and $Q{x}_{2,\text{f}}\left({t}_{\text{i}}\right)$ with respect to $x$, ${x}_{1}$ and ${x}_{2}$, respectively. Consequently, these amounts remain free and utilizable at the next modeling time point (${t}_{\text{i}+1}$).

In Fig. 2, $\cdots$ marks the potential existence of more (or the existence of none) of each types of components (protocells or nutrients) in the system, at ${t}_{\text{i}}$. The protocells are marked by rectangles, the nutrients are marked by circles in the figure. The (units of) nutrients absorbed by protocells are displayed within the corresponding protocells (rectangles). The crossed out protocells in the figure do not survive the competition, the nutrients within them (${x}_{\text{h}}$, ${x}_{1,\text{h}}$, ${x}_{2,\text{h}}$) get out to the environment (by the beginning of the next modeling time point ${t}_{\text{i}+1}$). The not crossed out protocells survive the competition. In the figure, only the nutrients circled by solid lines (not dashed lines) are included in the densities $Qx\left({t}_{\text{i}}\right)$, $Q{x}_{1}\left({t}_{\text{i}}\right)$, $Q{x}_{2}\left({t}_{\text{i}}\right)$, $Q{x}_{\text{h}}\left({t}_{\text{i}}\right)$, $Q{x}_{1,\text{h}}\left({t}_{\text{i}}\right)$, $Q{x}_{2,\text{h}}\left({t}_{\text{i}}\right)$, $Q{x}_{\text{f}}\left({t}_{\text{i}}\right)$, $Q{x}_{1,\text{f}}\left({t}_{\text{i}}\right)$ and $Q{x}_{2,\text{f}}\left({t}_{\text{i}}\right)$. In sub-figure *b*, the nutrients circled by dashed lines do not get out to the environment any more, they are utilized in the operation of the surviving protocells.

#### *Remark 2.2*

The density values can take not only (non-negative) integers (but also fractional numbers). This can be interpreted as, for example, the density of the protocells might be measured in g, kg, … Such kinds of units are generally used for different types of biomasses. For simplicity, it is assumed in this work, that the measures of the state variables are chosen in such a way that 1 unit of protocells require exactly 1 unit of each of the nutrients $x$, ${x}_{1}$ and ${x}_{2}$. This possible harmonization of the measures does not modify qualitatively the behavior of the dynamic model. Similarly, the time values in the model are assumed to be measured in proper units (minutes, days, weeks, years etc.), as well as the parameters ($b$, ${b}_{1}$ and ${b}_{2}$).

Let us introduce the following abbreviating symbols for the sum of different densities of the protocells:

$$QT: = QT_{0,1} + QT_{0,2} + QT_{1,1} + QT_{1,2} + QT_{2,1} + QT_{2,2} ,$$

$$QT_{0} : = QT_{0,1} + QT_{0,2} ,\;QT_{1} : = QT_{1,1} + QT_{1,2} ,\;QT_{2} : = QT_{2,1} + QT_{2,2} ,$$

$$Q\tilde{T}: = Q\tilde{T}_{0,1} + Q\tilde{T}_{0,2} + Q\tilde{T}_{1,1} + Q\tilde{T}_{1,2} + Q\tilde{T}_{2,1} + Q\tilde{T}_{2,2} ,$$

$$Q\tilde{T}_{0} : = Q\tilde{T}_{0,1} + Q\tilde{T}_{0,2} ,\;Q\tilde{T}_{1} : = Q\tilde{T}_{1,1} + Q\tilde{T}_{1,2} ,\;Q\tilde{T}_{2} : = Q\tilde{T}_{2,1} + Q\tilde{T}_{2,2} .$$

The detailed formal description of the mathematical model, at any modeling time point $t_{i}$, is according to Fig. 2. In the model below, seven main operation cases (Cases I, II, …, VII) and several sub-cases within them cover all possible operation circumstances in the competition.

First of all, the initial values should be set in the model:

$$Qx\left( 0 \right): = Qx_{{{\text{init}}}} ;\;Qx_{1} \left( 0 \right): = Qx_{{1,{\text{init}}}} ;\;Qx_{2} \left( 0 \right): = Qx_{{2,{\text{init}}}} .$$

If $t_{{\text{i}}} < t_{0,0}$, then $Q\tilde{T}_{0,1} \left( {t_{{\text{i}}} } \right): = Q\tilde{T}_{0,2} \left( {t_{{\text{i}}} } \right): = 0$. If $t_{{\text{i}}} = t_{0,0}$, then $Q\tilde{T}_{0,1} \left( {t_{{\text{i}}} } \right): = QT_{{0,1,{\text{init}}}}$ and $Q\tilde{T}_{0,2} \left( {t_{{\text{i}}} } \right): = QT_{{0,2,{\text{init}}.}}$

If $t_{{\text{i}}} < t_{1,0}$, then $Q\tilde{T}_{1,1} \left( {t_{{\text{i}}} } \right): = Q\tilde{T}_{1,2} \left( {t_{{\text{i}}} } \right): = 0$. If $t_{{\text{i}}} = t_{1,0}$, then $Q\tilde{T}_{1,1} \left( {t_{{\text{i}}} } \right): = QT_{{1,1,{\text{init}}}}$ and $Q\tilde{T}_{1,2} \left( {t_{{\text{i}}} } \right): = QT_{{1,2,{\text{init}}.}}$

If $t_{{\text{i}}} < t_{2,0}$, then $Q\tilde{T}_{2,1} \left( {t_{{\text{i}}} } \right): = Q\tilde{T}_{2,2} \left( {t_{{\text{i}}} } \right): = 0$. If $t_{{\text{i}}} = t_{2,0}$, then $Q\tilde{T}_{2,1} \left( {t_{{\text{i}}} } \right): = QT_{{2,1,{\text{init}}}}$ and $Q\tilde{T}_{2,2} \left( {t_{{\text{i}}} } \right): = QT_{{2,2,{\text{init}}}} .$

In the following, the seven operation cases (Cases I, II, …, VII) are detailed. (For only Case I, detailed comments are provided. The other operation cases could be explained similarly.)

I. ${\varvec{t}}_{{\mathbf{i}}} = {\varvec{h}}_{{{{\varvec{\uptau}}}_{0} }} \left( {\varvec{i}} \right)$ and ${\varvec{t}}_{{\mathbf{i}}} \ne {\varvec{h}}_{{{{\varvec{\uptau}}}_{1} }} \left( {\varvec{i}} \right)$ (${\varvec{t}}_{{\mathbf{i}}} > {\varvec{h}}_{{{{\varvec{\uptau}}}_{1} }} \left( {\varvec{i}} \right)$ or ${\varvec{h}}_{{{{\varvec{\uptau}}}_{1} }} \left( {\varvec{i}} \right)$ is not defined) and ${\varvec{t}}_{{\mathbf{i}}} \ne {\varvec{h}}_{{{{\varvec{\uptau}}}_{2} }} \left( {\varvec{i}} \right)$ (${\varvec{t}}_{{\mathbf{i}}} > {\varvec{h}}_{{{{\varvec{\uptau}}}_{2} }} \left( {\varvec{i}} \right)$ or ${\varvec{h}}_{{{{\varvec{\uptau}}}_{2} }} \left( {\varvec{i}} \right)$ is not defined)

In this case, $t_{{\text{i}}}$ is an operation time point of $T_{0}$ and not an operation time point of $T_{1}$ and $T_{2}$.

1. Densities just before the competition

This sub-case corresponds to sub-figure *a*) in Fig. 2.

1. $${\varvec{t}}_{{\mathbf{i}}} = 0$$

$t_{{\text{i}}}$ Is the initial (first) modeling time point. Different densities are not to be determined in this case. Go to the determination of the densities just after the competition, that is, to sub-case I.2 below.

1. $${\varvec{t}}_{{\mathbf{i}}} > 0$$

$t_{{\text{i}}}$ Is not the initial modeling time point (yet).

$$Q\tilde{T}_{1,1} \left( {t_{{\text{i}}} } \right) = QT_{1,1} \left( {t_{{{\text{i}} - 1}} } \right),$$

$$Q\tilde{T}_{1,2} \left( {t_{{\text{i}}} } \right) = QT_{1,2} \left( {t_{{{\text{i}} - 1}} } \right),$$

$$Q\tilde{T}_{2,1} \left( {t_{{\text{i}}} } \right) = QT_{2,1} \left( {t_{{{\text{i}} - 1}} } \right),$$

$$Q\tilde{T}_{2,2} \left( {t_{{\text{i}}} } \right) = QT_{2,2} \left( {t_{{{\text{i}} - 1}} } \right),$$

$$Qx\left( {t_{{\text{i}}} } \right) = max\left( {Qx_{{\text{f}}} \left( {t_{{{\text{i}} - 1}} } \right) + Qx_{{\text{h}}} \left( {t_{{{\text{i}} - 1}} } \right) + b\left( {t_{{\text{i}}} - t_{{{\text{i}} - 1}} } \right),{ }0} \right),$$

where $b\left( {t_{{\text{i}}} - t_{{{\text{i}} - 1}} } \right)$ is the density of nutrient $x$ produced in the environment during the time period $\left[ {t_{{{\text{i}} - 1}} , t_{{\text{i}}} } \right]$. (In case of negative values, the nutrient is actually not produced but decayed in the environment.) The operation $max$ assures that negative (meaningless) amounts of nutrient cannot exist in the environment.

- $${\varvec{t}}_{{\mathbf{i}}} = {\varvec{t}}_{0,0}$$

In this case, $t_{{\text{i}}}$ is the initial (first) operation time point of $T_{0}$.

$$Qx_{1} \left( {t_{{\text{i}}} } \right) = max\left( {Qx_{{1,{\text{f}}}} \left( {t_{{{\text{i}} - 1}} } \right) + Qx_{{1,{\text{h}}}} \left( {t_{{{\text{i}} - 1}} } \right) + b_{1} \left( {t_{{\text{i}}} - t_{{{\text{i}} - 1}} } \right),{ }0} \right),$$

$$Qx_{2} \left( {t_{{\text{i}}} } \right) = max\left( {Qx_{{2,{\text{f}}}} \left( {t_{{{\text{i}} - 1}} } \right) + Qx_{{2,{\text{h}}}} \left( {t_{{{\text{i}} - 1}} } \right) + b_{2} \left( {t_{{\text{i}}} - t_{{{\text{i}} - 1}} } \right),{ }0} \right),$$

where the explanation for the $max$ operation is similar to that above, used in the determination of $Qx\left( {t_{{\text{i}}} } \right)$.

1. $${\varvec{t}}_{{\mathbf{i}}} > {\varvec{t}}_{0,0}$$

In this case, $t_{{\text{i}}}$ is not the initial operation time point of $T_{0}$ (yet).

$$Q\tilde{T}_{0,1} \left( {t_{{\text{i}}} } \right) = QT_{0} \left( {h_{{{\uptau }_{0} }} \left( {i - 1} \right)} \right),$$

$$Q\tilde{T}_{0,2} \left( {t_{{\text{i}}} } \right) = QT_{0,1} \left( {h_{{{\uptau }_{0} }} \left( {i - 1} \right)} \right),$$

$$Qx_{1} \left( {t_{{\text{i}}} } \right) = max\left( {Qx_{{1,{\text{f}}}} \left( {t_{{{\text{i}} - 1}} } \right) + Qx_{{1,{\text{h}}}} \left( {t_{{{\text{i}} - 1}} } \right) + b_{1} \left( {t_{{\text{i}}} - t_{{{\text{i}} - 1}} } \right) + QT_{0} \left( {h_{{{\uptau }_{0} }} \left( {i - 1} \right)} \right),{ }0} \right),$$

$$Qx_{2} \left( {t_{{\text{i}}} } \right) = max\left( {Qx_{{2,{\text{f}}}} \left( {t_{{{\text{i}} - 1}} } \right) + Qx_{{2,{\text{h}}}} \left( {t_{{{\text{i}} - 1}} } \right) + b_{2} \left( {t_{{\text{i}}} - t_{{{\text{i}} - 1}} } \right) + QT_{0} \left( {h_{{{\uptau }_{0} }} \left( {i - 1} \right)} \right),{ }0} \right),$$

where the explanation for the $max$ operation is similar to that above, used in the determination of $Qx\left( {t_{{\text{i}}} } \right)$.

I.2. Densities just after the competition

This sub-case corresponds to sub-figure *b*) in Fig. 2.

Determination of the density of the $T_{0,1}$-type protocells surviving the competition ($QT_{0,1} \left( {t_{{\text{i}}} } \right)$)

This density ($QT_{0,1} \left( {t_{{\text{i}}} } \right)$) is that of the $T_{0,1}$-type protocells which (expectedly) get (the needed) nutrient $x$ (protocells of density $Q\tilde{T}_{0} \left( {t_{{\text{i}}} } \right)$ compete for $x$ of density $Qx\left( {t_{{\text{i}}} } \right)$):

If $Q\tilde{T}_{0,1} \left( {t_{{\text{i}}} } \right) = 0$ or $Qx\left( {t_{{\text{i}}} } \right) = 0$, then

$$QT_{0,1} \left( {t_{{\text{i}}} } \right) = 0,$$

Otherwise, computing with expected value,

$$QT_{0,1} \left( {t_{{\text{i}}} } \right) = min\left( {\frac{{Q\tilde{T}_{0,1} \left( {t_{{\text{i}}} } \right)Qx\left( {t_{{\text{i}}} } \right)}}{{Q\tilde{T}_{0} \left( {t_{{\text{i}}} } \right)}},{ }Q\tilde{T}_{0,1} \left( {t_{{\text{i}}} } \right)} \right),$$

where the first term (the fraction) in the brackets is the (expected) density of the ${T}_{\text{0,1}}$-type protocells which get nutrient $x$ in the competition (at modeling time point ${t}_{\text{i}}$), assuming that all competing protocells have even chances to get the needed nutrients. If the available density of nutrient $x$ before the competition were higher than the density of the protocells competing for $x$ ($Qx\left( {t_{{\text{i}}} } \right) > Q\tilde{T}_{0} \left( {t_{{\text{i}}} } \right)$), then the density of the surviving $T_{0,1}$-type protocells would result in a higher value than their initial density ($Q\tilde{T}_{0,1} \left( {t_{{\text{i}}} } \right)$), which is impossible. This is excluded by the operation $min$ that maximizes the density of the surviving $T_{0,1}$-type protocells (at the value $Q\tilde{T}_{0,1} \left( {t_{{\text{i}}} } \right)$).

Determination of the density of the $T_{0,2}$-type protocells surviving the competition ($QT_{0,2} \left( {t_{i} } \right)$)

This density ($QT_{{0,2,{\text{x}}}} \left( {t_{{\text{i}}} } \right)$) is that of the $T_{0,2}$-type protocells which get nutrient $x$ (there is still $Qx\left( {t_{{\text{i}}} } \right) - QT_{0,1} \left( {t_{i} } \right)$ of $x$ “after” the share of the $T_{0,1}$-type protocells, which is completely left for the $T_{0,2}$-type protocells):

$$QT_{{0,2,{\text{x}}}} \left( {t_{{\text{i}}} } \right) = min\left( {Qx\left( {t_{{\text{i}}} } \right) - QT_{0,1} \left( {t_{i} } \right),{ }Q\tilde{T}_{0,2} \left( {t_{{\text{i}}} } \right)} \right).$$

This density ($QT_{{0,2,{\text{x}},{\text{x}}1}} \left( {t_{{\text{i}}} } \right)$) is that of the $T_{0,2}$-type protocells which get both nutrients $x$ and $x_{1}$ (protocells of density $Q\tilde{T}_{0,2} \left( {t_{{\text{i}}} } \right)$ compete for $x_{1}$ of density $Qx_{1} \left( {t_{{\text{i}}} } \right)$):

If $QT_{{0,2,{\text{x}}}} \left( {t_{{\text{i}}} } \right) = 0$ or $Qx_{1} \left( {t_{{\text{i}}} } \right) = 0$, then

$$QT_{{0,2,{\text{x}},{\text{x}}1}} \left( {t_{{\text{i}}} } \right) = 0.$$

Othe﻿rwise (computing with expected value, similarly as above, in the determination of $QT_{0,1} \left( {t_{{\text{i}}} } \right)$),

$$QT_{{0,2,{\text{x}},{\text{x}}1}} \left( {t_{{\text{i}}} } \right) = min\left( {\frac{{QT_{{0,2,{\text{x}}}} \left( {t_{{\text{i}}} } \right)Qx_{1} \left( {t_{{\text{i}}} } \right)}}{{Q\tilde{T}_{0,2} \left( {t_{{\text{i}}} } \right)}},{ }QT_{{0,2,{\text{x}}}} \left( {t_{{\text{i}}} } \right)} \right).$$

This density ($QT_{0,2} \left( {t_{{\text{i}}} } \right)$) is that of the $T_{0,2}$-type protocells which get all of nutrients $x$, $x_{1}$ and $x_{2}$ (protocells of density $Q\tilde{T}_{0,2} \left( {t_{{\text{i}}} } \right)$ compete for $x_{2}$ of density $Qx_{2} \left( {t_{{\text{i}}} } \right)$):

If $QT_{{0,2,{\text{x}},{\text{x}}1}} \left( {t_{{\text{i}}} } \right) = 0$ or $Qx_{2} \left( {t_{{\text{i}}} } \right) = 0$, then

$$QT_{0,2} \left( {t_{{\text{i}}} } \right) = 0,$$

Otherwise (computing with expected value, similarly as above, in the determination of $QT_{0,1} \left( {t_{{\text{i}}} } \right)$),

$$QT_{0,2} \left( {t_{{\text{i}}} } \right) = min\left( {\frac{{QT_{{0,2,{\text{x}},{\text{x}}1}} \left( {t_{{\text{i}}} } \right)Qx_{2} \left( {t_{{\text{i}}} } \right)}}{{Q\tilde{T}_{0,2} \left( {t_{{\text{i}}} } \right)}},{ }QT_{{0,2,{\text{x}},{\text{x}}1}} \left( {t_{{\text{i}}} } \right)} \right).$$

This density of $x$ is not absorbed by the protocells in the competition (surplus of $x$):

$$Qx_{{\text{f}}} \left( {t_{{\text{i}}} } \right) = max\left( {Qx\left( {t_{{\text{i}}} } \right) - Q\tilde{T}_{0} \left( {t_{{\text{i}}} } \right),{ }0} \right),$$

where the explanation for the $max$ operation is similar to that above, used in the determination of $Qx\left( {t_{{\text{i}}} } \right)$.

This is the density of nutrient $x$ which is absorbed by the not surviving protocells (which go extinct by the next modeling time point $t_{{{\text{i}} + 1}}$) and, consequently, get out to the environment by the next modeling time point $t_{{{\text{i}} + 1}}$:

$$Qx_{{\text{h}}} \left( {t_{{\text{i}}} } \right) = min\left( {Qx\left( {t_{{\text{i}}} } \right) - QT_{0,1} \left( {t_{{\text{i}}} } \right),{ }Q\tilde{T}_{0,2} \left( {t_{{\text{i}}} } \right)} \right) - QT_{0,2} \left( {t_{{\text{i}}} } \right).$$

This density of $x_{1}$ is not absorbed by the protocells in the competition (surplus of $x_{1}$):

$$Qx_{{1,{\text{f}}}} \left( {t_{{\text{i}}} } \right) = max\left( {Qx_{1} \left( {t_{{\text{i}}} } \right) - Q\tilde{T}_{0,2} \left( {t_{{\text{i}}} } \right),{ }0} \right),$$

where the explanation for the $max$ operation is similar to that above, used in the determination of $Qx\left( {t_{{\text{i}}} } \right)$.

This is the density of nutrient $x_{1}$ absorbed by the not surviving protocells (which go extinct by the next modeling time point $t_{{{\text{i}} + 1}}$) and, consequently, get out to the environment by the next modeling time point $t_{{{\text{i}} + 1}}$:

$$Qx_{{1,{\text{h}}}} \left( {t_{{\text{i}}} } \right) = min\left( {Qx_{1} \left( {t_{{\text{i}}} } \right),{ }Q\tilde{T}_{0,2} \left( {t_{{\text{i}}} } \right)} \right) - QT_{0,2} \left( {t_{{\text{i}}} } \right).$$

This density of $x_{2}$ is not absorbed by the protocells in the competition (surplus of $x_{2}$):

$$Qx_{{2,{\text{f}}}} \left( {t_{{\text{i}}} } \right) = max\left( {Qx_{2} \left( {t_{{\text{i}}} } \right) - Q\tilde{T}_{0,2} \left( {t_{{\text{i}}} } \right),{ }0} \right),$$

where the explanation for the $max$ operation is similar to that above, used in the determination of $Qx\left( {t_{{\text{i}}} } \right)$.

This is the density of nutrient $x_{2}$ absorbed by the not surviving protocells (which go extinct by the next modeling time point $t_{{{\text{i}} + 1}}$) and, consequently, get out to the environment by the next modeling time point $t_{{{\text{i}} + 1}}$:

$$Qx_{{2,{\text{h}}}} \left( {t_{{\text{i}}} } \right) = min\left( {Qx_{2} \left( {t_{{\text{i}}} } \right),{ }Q\tilde{T}_{0,2} \left( {t_{{\text{i}}} } \right)} \right) - QT_{0,2} \left( {t_{{\text{i}}} } \right).$$

The densities of those protocell types are not changed (in the competition) for which the current modeling time point ($t_{{\text{i}}}$) is not an operation time point:

$$QT_{1,1} \left( {t_{{\text{i}}} } \right) = Q\tilde{T}_{1,1} \left( {t_{{\text{i}}} } \right),$$

$$QT_{1,2} \left( {t_{{\text{i}}} } \right) = Q\tilde{T}_{1,2} \left( {t_{{\text{i}}} } \right),$$

$$QT_{2,1} \left( {t_{{\text{i}}} } \right) = Q\tilde{T}_{2,1} \left( {t_{{\text{i}}} } \right),$$

$$QT_{2,2} \left( {t_{{\text{i}}} } \right) = Q\tilde{T}_{2,2} \left( {t_{{\text{i}}} } \right).$$

The formulas of the other operation cases (Cases II, III, …, VII) can be found in Appendix A (in the Supplementary Information).

## Results of the Dynamic Model and Their Biological Interpretation

In this section, the results of some (selected) computer experiments with the extended model, along with their biological interpretations, and the exact mathematical analysis of a particular equilibrium of the protocell species are presented.

### Computer Experiments with the Extended Model

We programmed the discrete-time population model of Sect. 2 in the Matlab software (Attaway 2019) for simulation purposes. In this manner, the modelled change of the different densities (of the protocells and the nutrients) over time can be observed directly.

**Fig. 3:**
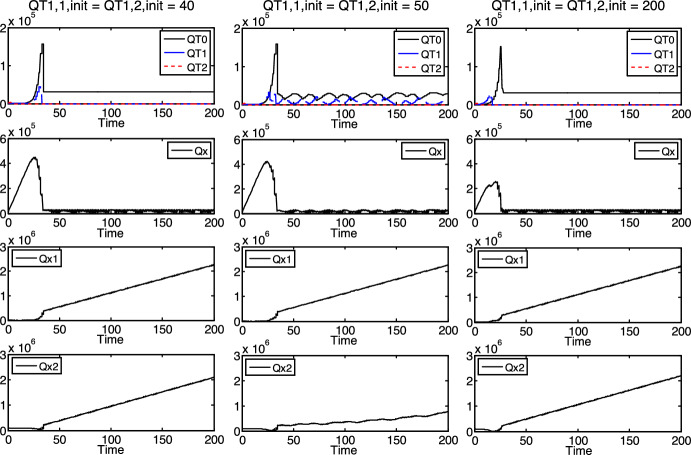
Dependence of the survival of $T_{1}$ on its initial density, incommensurable reproduction times

**Fig. 4:**
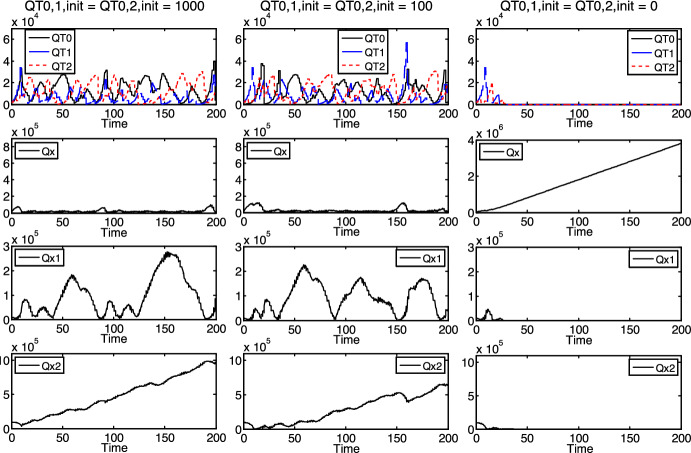
Dependence of the survival of $T_{1}$ and $T_{2}$ on the existence of $T_{0}$ (as a keystone species), incommensurable reproduction times

**Fig. 5:**
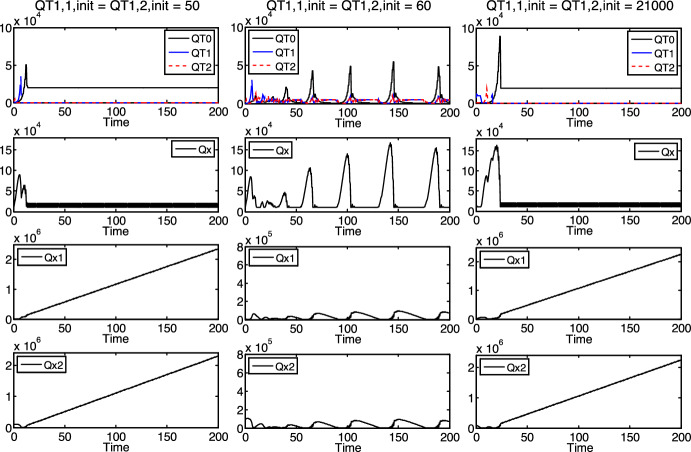
Dependence of the survival of $T_{1}$ on its initial density, commensurable reproduction times

**Fig. 6:**
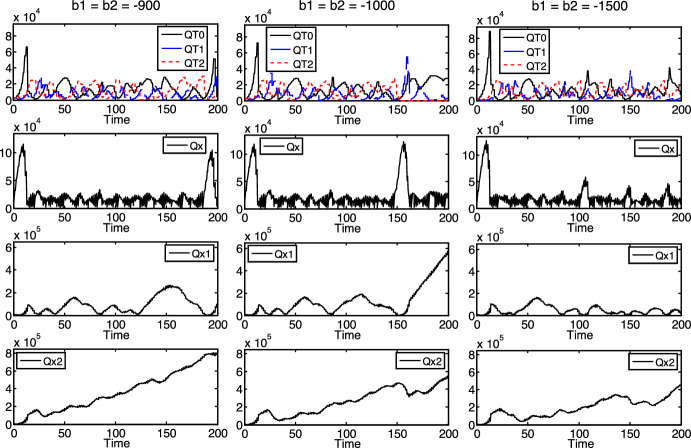
Dependence of the survival of $T_{2}$ on $b_{1}$ and $b_{2}$, incommensurable reproduction times

**Fig. 7:**
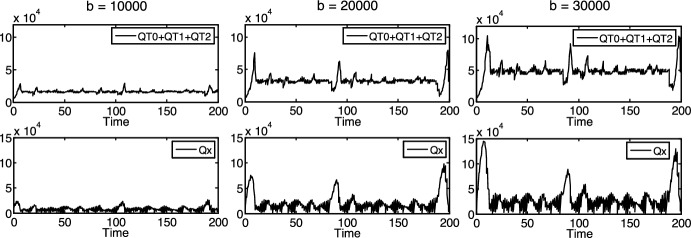
Dependence of the protocells’ overall density ($QT_{0} + QT_{1} + QT_{2}$) on $b$, incommensurable reproduction times

By means of running the model in numerical simulations, while setting different parameter and initial values, we could already detect several interesting and important aspects of the model. The studied modeling time interval is 200 (in proper time units) in the below experiments, which is assumed to be enough for our prediction aims. For the representation of the gained results in this section (in Figs. 3, 4, 5, 6 and 7), let us use the already defined variables $QT_{0} = QT_{0,1} + QT_{0,2}$, $QT_{1} = QT_{1,1} + QT_{1,2}$ and $QT_{2} = QT_{2,1} + QT_{2,2}$.

One of the important phenomena observed in the computer experiments is the dependence of the survival on the initial density of a given species in an ecosystem (see Fig. 3).

Namely, under the setup of certain initial and parameter values ($b = 20000$, $b_{1} = b_{2} = - 1000$, $\tau_{0} = \pi /2 \approx 1.57$, $\tau_{1} = \tau_{0} \cdot \pi /4 + 0.2 \approx 1.43$, $\tau_{2} = 1.7$, $t_{0,0} = t_{1,0} = t_{2,0} = 0$, $QT_{{0,1,{\text{init}}}} = QT_{{0,2,{\text{init}}}} = 100$, $QT_{{2,1,{\text{init}}}} = QT_{{2,2,{\text{init}}}} = 1000$, $Qx_{{{\text{init}}}} = Qx_{{1,{\text{init}}}} = 10000$, $Qx_{{2,{\text{init}}}} = 100000$), changing only the initial density of the $T_{1}$ specialist, it goes extinct if its initial density is relatively low ($QT_{{1,1,{\text{init}}}} = QT_{{1,2,{\text{init}}}} = 40$, see the first column in Fig. 3), and it survives if this value is a bit higher ($QT_{{1,1,{\text{init}}}} = QT_{{1,2,{\text{init}}}} = 50$, see the second column in Fig. 3). This difference is in accordance with the competitive exclusion principle (Gause 1932; Hardin 1960). If the initial density is even higher ($QT_{{1,1,{\text{init}}}} = QT_{{1,2,{\text{init}}}} = 200$, see the third column in Fig. 3), then $T_{1}$ goes extinct again, maybe because of that its relatively high density raises the overall density of the whole ecosystem too much to maintain both species (both $T_{1}$ and $T_{2}$). Then, in the resulted, more intensive, competition for the limited resources (nutrients), the less fit species (seemingly $T_{1}$) goes extinct—according to the concept of natural selection (Darwin 1859).

In connection with the above setup, the absolutely different (even incommensurable) reproduction times, $\tau_{0} \ne \tau_{1}$, $\tau_{0} \ne \tau_{2}$, $\tau_{1} \ne \tau_{2}$, represent the essential novelty of the population model, which is more general than the preliminary model, where $\tau_{1} = \tau_{2}$ was required.

Another phenomenon, observed in the simulations, relates to the concept of keystone species (see Fig. 4).

Namely, under certain initial and parameter values ($b = 20000$, $b_{1} = b_{2} = - 500$, $\tau_{0} = \pi /2 \approx 1.57$, $\tau_{1} = \tau_{0} \cdot \pi /4 + 0.2 \approx 1.43$, $\tau_{2} = 1.7$, $t_{0,0} = t_{1,0} = t_{2,0} = 0$, $QT_{{1,1,{\text{init}}}} = QT_{{1,2,{\text{init}}}} = QT_{{2,1,{\text{init}}}} = QT_{{2,2,{\text{init}}}} = 1000$, $Qx_{{{\text{init}}}} = Qx_{{1,{\text{init}}}} = 10000$, $Qx_{{2,{\text{init}}}} = 100000$), changing only the initial density of the $T_{0}$ generalist, the ($T_{1}$ and $T_{2}$) specialists survive if the generalist is present in the ecosystem either at a relatively high ($QT_{{0,1,{\text{init}}}} = QT_{{0,2,{\text{init}}}} = 1000$, see the first column in Fig. 4) or only at a relatively low ($QT_{{0,1,{\text{init}}}} = QT_{{0,2,{\text{init}}}} = 100$, see the second column in Fig. 4) level. However, they go extinct if the generalist is not present ($QT_{{0,1,{\text{init}}}} = QT_{{0,2,{\text{init}}}} = 0$, see the third column in Fig. 4). It is in accordance with that the generalist is a keystone species.

Since the proposed model is more general than the preliminary model, it can be used for the same parameter sets as the preliminary model as well, that is, for the case of equal reproduction times of the specialists ($\tau_{1} = \tau_{2}$). In such cases, we could explore essentially the same phenomena in the ecosystem as above (in Figs. 3 and 4, where the reproduction times were different), in our computer experiments. For example, we could detect the dependence of the survival of a species on its initial density (similarly as in Fig. 3), see Fig. 5 (where $b = 20000$, $b_{1} = b_{2} = - 500$, $\tau_{0} = 1$, $\tau_{1} = \tau_{2} = 0.5$, $t_{0,0} = t_{1,0} = t_{2,0} = 0$, $QT_{{0,1,{\text{init}}}} = QT_{{0,2,{\text{init}}}} = 100$, $QT_{{2,1,{\text{init}}}} = QT_{{2,2,{\text{init}}}} = 1000$, $Qx_{{{\text{init}}}} = Qx_{{1,{\text{init}}}} = 10000$, $Qx_{{2,{\text{init}}}} = 100000$).

More precisely, in Fig. 5, $T_{1}$ and $T_{2}$ always go extinct or survive together, depending on the initial density of $T_{1}$. (The equal reproduction times of the specialists may be responsible for their joint behavior.) Accordingly, both specialists go extinct if the initial density of $T_{1}$ is relatively low ($QT_{{1,1,{\text{init}}}} = QT_{{1,2,{\text{init}}}} = 50$, see the first column in Fig. 5) and survive if this value is a bit higher ($QT_{{1,1,{\text{init}}}} = QT_{{1,2,{\text{init}}}} = 60$, see the second column in Fig. 5), which represents the competitive exclusion principle. If the initial density of $T_{1}$ is even higher ($QT_{{1,1,{\text{init}}}} = QT_{{1,2,{\text{init}}}} = 21000$, see the third column in Fig. 5), then both specialists go extinct again, according to the collapse of the population because of the limited resources.

Interestingly, we could detect a special phenomenon with the model in case of different reproduction times, which we could not detect if $\tau_{1} = \tau_{2}$. Accordingly, we could not observe it in the preliminary model either, so it is a novelty compared to the preliminary model. This is a certain “anomaly” depending on the rates of decrease of nutrients $x_{1}$ and $x_{2}$ in the environment, that is, on $b_{1}$ and $b_{2}$ (with negative values expressing decrease instead of increase), see Fig. 6.

Namely, under the setup of certain initial and parameter values ($b = 20000$, $\tau_{0} = \pi /2 \approx 1.57$, $\tau_{1} = \tau_{0} \cdot \pi /4 + 0.2 \approx 1.43$, $\tau_{2} = 1.7$, $t_{0,0} = t_{1,0} = t_{2,0} = 0$, $QT_{{0,1,{\text{init}}}} = QT_{{0,2,{\text{init}}}} = QT_{{1,1,{\text{init}}}} = QT_{{1,2,{\text{init}}}} = QT_{{2,1,{\text{init}}}} = QT_{{2,2,{\text{init}}}} = 1000$, $Qx_{{{\text{init}}}} = Qx_{{1,{\text{init}}}} = Qx_{{2,{\text{init}}}} = 10000$), changing only $b_{1}$ and $b_{2}$, the $T_{2}$ specialist survives at a certain rate of decrease ($b_{1} = b_{2} = - 900$, see the first column in Fig. 6, from about the time instant 150), and it goes extinct if the decreasing of nutrients $x_{1}$ and $x_{2}$ is a bit more intensive ($b_{1} = b_{2} = - 1000$, see the second column in Fig. 6, from about the time instant 150). Surprisingly, in contrast to the normal expectations, $T_{2}$ survives again if the rate of decrease is even higher (in absolute value) ($b_{1} = b_{2} = - 1500$, see the third column in Fig. 6). (Of course, above a certain rate of decrease level, $b_{1} = b_{2} \ll - 1500$, not presented in Fig. 6, $T_{2}$ will always die out normally.)

A possible explanation of this “anomaly”, at a heuristic level, is the following: First of all, the situation of $T_{1}$ (in the case of Fig. 6) is basically more advantageous in the competition than that of $T_{2}$ since $T_{1}$ reproduces faster ($\tau_{1} < \tau_{2}$). It seems that below a certain rate of decrease of nutrients $x_{1}$ and $x_{2}$, there are enough nutrients for both specialists to survive (see the first column in Fig. 6). However, if the decreasing is more intensive (up to a certain level), then the amount of nutrients $x_{1}$ and $x_{2}$ may be still enough for $T_{1}$ to stay at a relatively strong position but not enough yet for the less fit $T_{2}$. Seemingly, this biased situation (against $T_{2}$) could result in that $T_{1}$ is able to overcome $T_{2}$ in such a way that $T_{2}$ dies out, while $T_{1}$ survives (see the second column in Fig. 6, from about time 150). Perhaps, if the rate of decrease of $x_{1}$ and $x_{2}$ is even higher, the nutrients are not enough for either specialist to stay at a relatively strong position. This may result in a less biased (mutually disadvantageous) situation and that $T_{1}$ cannot overcome $T_{2}$. Up to a certain rate of decrease level, it may cause that both specialists are able to survive again (see the second column in Fig. 6).

It can be seen in Figs. 3, 4, 5 and 6 that, if a kind of protocells survives, its density fluctuates in a highly aperiodic way, with relatively high amplitudes. The only exceptional case is when the generalist survives and both specialists go extinct (see the first and third columns in Figs. 3 and 5). In the latter case, it seems that the density of the generalist tends to an (asymptotically stable) equilibrium. (This equilibrium of the first column in Fig. 5 is proved analytically in Sect. 3.2 below.) Otherwise, the non-linearity of the mathematical model should be responsible for the mentioned fluctuations.

In addition, Figs. 3, 4, 5 and 6 also suggest that the overall density of the protocells ($QT = QT_{0} + QT_{1} + QT_{2}$) is limited, mostly by the density of nutrient $x$ ($Qx$), which clearly has an upper bound nearly everywhere in Figs. 3, 4, 5 and 6. (The only exception is the third column in Fig. 4, where each kind of protocells goes extinct or does not exist at all.) It suggests that the density of nutrient $x$, or in other words, its rate of increase $b$ functions (and might be used in bioengineering applications) as an external control over the overall density of the protocells. It is well supported by the simulation results in Fig. 7 (where $b_{1} = b_{2} = - 500$, $\tau_{0} = \pi /2 \approx 1.57$, $\tau_{1} = \tau_{0} \cdot \pi /4 + 0.2 \approx 1.43$, $\tau_{2} = 1.7$, $t_{0,0} = t_{1,0} = t_{2,0} = 0$, $QT_{{0,1,{\text{init}}}} = QT_{{0,2,{\text{init}}}} = QT_{{1,1,{\text{init}}}} = QT_{{1,2,{\text{init}}}} = QT_{{2,1,{\text{init}}}} = QT_{{2,2,{\text{init}}}} = 1000$, $Qx_{{{\text{init}}}} = Qx_{{1,{\text{init}}}} = 10000$, $Qx_{{2,{\text{init}}}} = 100000$), where $QT_{0} + QT_{1} + QT_{2}$ increases with increasing $b$ values.

In particular, the mean value of $QT$ in Fig. 7 is 16184 in the first column, 32305 in the second column and 48474 in the third column, where $b$ is 10,000, 20,000 and 30,000, respectively (and the mean value of $Qx$ is 7881, 20568 and 31591, respectively).

### Exact Analysis of the Equilibrium of the Generalist

**Fig. 8:**
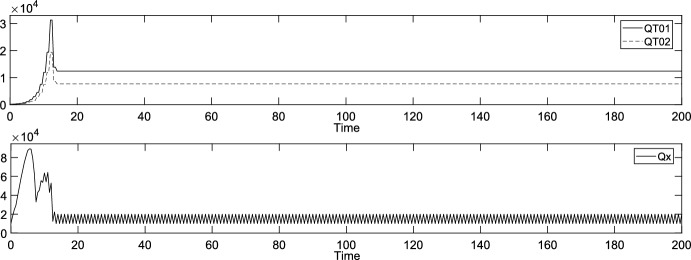
Densities of the young and old generalists, $T_{0,1}$ and $T_{0,2}$, and nutrient $x$ in the case of the first column of Fig. 5

It is of special importance, in general, to analyze the stability of equilibria in competitive multi-species systems (see e.g. (Varga and Szathmáry 1997)). Based on the above experiments, we could observe no equilibria regarding the protocells’ densities when the specialists survive. Only the above highly aperiodic behavior was observed in such cases. Accordingly, if not all protocells go completely extinct, only the apparent (positive) equilibrium regarding the generalist’s density can be examined analytically, in case of the specialists’ extinction. Such an equilibrium can be seen in the first column of Fig. 5, where the reproduction times of the specialist are equal and half of that of the generalist ($\tau_{1} = \tau_{2} = \tau_{0} /2 = 0.5$). Let us study this equilibrium in this section below. Figure 8 enlarges the graphs of the corresponding densities of the generalist and nutrient $x$ (cf. the first and second sub-figures in the first column of Fig. 5). More precisely, not the overall density of the generalist ($QT_{0}$) but the densities of its young and old varieties ($QT_{0,1}$ and $QT_{0,2}$) are displayed (and the densities of the specialists are not displayed) in the first sub-figure of Fig. 8.

One can easily see from the mathematical representation of the model (in Sect. 2) that, strictly speaking, this is not an equilibrium regarding the densities. This is rather a periodic orbit with a short period (a period of 2 time steps)—except $Qx_{1}$ and $Qx_{2}$, which become monotonically increasing, not constant (see the third and fourth sub-figures in the first column of Fig. 5). It is suggested by the computer experiments that this “motion” alternates between the operation cases VI.1/B/*d* and VII.1/B/*h*. At every odd-numbered modeling time step/point (time points $t_{1} = \tau_{1} = 0.5$, $t_{3} = 3\tau_{1} = 1.5$, $t_{5} = 5\tau_{1} = 2.5$, …), only the specialists reproduce. At every even-numbered modeling time step/point (time points $t_{2} = 2\tau_{1} = 1$, $t_{4} = 4\tau_{1} = 2$, $t_{6} = 3\tau_{1} = 3$, …), both the generalist and the specialists reproduce.

Taking the advantage of that the specialists’ densities are zero at the equilibrium and that $t_{{{\text{i}} + 1}} - t_{{\text{i}}} = \tau_{1} = 0.5$ $\left( {i = 0, 1, 2, \ldots } \right)$, we can build each odd-numbered modeling time point in the next even-numbered modeling time point. In this way, we get an autonomous time-contracted dynamic model expressing the densities at every second consecutive modeling time point, that is, at the end of all whole reproductive cycles of the generalist (at $t = 1, 2, 3, \ldots$).

Regarding the time-contracted model, the computer experiments suggest that the discussed equilibrium is locally asymptotically stable (excluding the strictly monotonically increasing variables $Qx_{1}$ and $Qx_{2}$). Consequently, it is assumed that $Qx_{1}$ and $Qx_{2}$ are always enough to completely supply the generalist (of density $QT_{0} = QT_{0,1} + QT_{0,2}$) with nutrients $x_{1}$ and $x_{2}$. Then the equilibrium and its stability can be checked (as well as the validity of the assumption itself). See Appendix B (in the Supplementary Information) for the details (and e.g. (Elaydi 2005)). The Wolfram Mathematica software was applied for the analysis (Wolfram 2003). Finally, the resulted equilibrium is

$$\left( {Qx^{*} ,{ }QT_{0,1}^{*} ,{ }QT_{0,2}^{*} } \right) = \left( {b,{ }\frac{\sqrt 5 - 1}{2}b,{ }\left( {1 - \frac{\sqrt 5 - 1}{2}} \right)b} \right).$$

It is locally asymptotically stable with respect to the subspace of variables $Qx$, $QT_{0,1}$ and $QT_{0,2}$. This equilibrium, along with its stability, is in accordance with the observations in the computer experiments. See the case of Fig. 8, where $b = 20000$ and this equilibrium is

$$\left( {b,{ }\frac{\sqrt 5 - 1}{2}b,{ }\left( {1 - \frac{\sqrt 5 - 1}{2}} \right)b} \right) \approx \left( {20000,{ }12361,{ }7639} \right).$$

Interestingly, the golden ratio, known e.g. from the celebrated Fibonacci sequence, appears in the equilibrium.

## Conclusions

It is of great importance to model the behavior of basic protocell communities (as protocells are basic life-like artificial organisms) as precisely as possible since understanding them better may help to describe more complex ecological systems, too.

In the present paper, we worked out an extended, completely reformulated version (extended model) of a recently developed discrete-time dynamic population model (preliminary model). The extended mathematical model is more general than the preliminary model as it can handle completely different reproduction times and initial operation time points of the protocell species (a generalist and two specialists).

The model counts with the species’ different reproduction times and that they produce different types of nutrients. The advantage for the generalist is that it produces more kinds of nutrients than the specialists do. The limited lifetime and the current age of the protocells as well as the environment’s nutrient accumulating and decaying effects are also included.

Our work was aimed at a model which is as basic/fundamental as possible (with only three modelled species) while already displays complex population dynamics behavior (like competitive exclusion and keystone species), in computer experiments.

In order to indicate the practical applicability of the model, we emphasize that it can present the competitive exclusion principle, well-known in population ecology. Namely, under the same model parameter values, the density of a given specialist remains at a relatively high level (although, displaying a highly aperiodic behavior) if its initial value is above a certain threshold value, while the specialist goes extinct if its initial density starts from below that value. The generalist, instead, survives in both cases.

It is interesting to point out that the dynamic model also showed that the generalist’s bare presence (at least at a low level), is also of primary importance concerning the survival of the specialists. Namely, both specialists go extinct if the generalist’s density is zero. This phenomenon is supporting for the general concept of the species protection program that it may be important to preserve any (seemingly perhaps insignificant) species since it might be a keystone species meaning that it might be of primary importance concerning the survival of other species. In addition, the extended model could produce a certain “anomaly” with respect to the connection between the survival of a given species and the rates of decrease of certain nutrients in the environment. This “anomaly” is a new phenomenon of the extended model (and of the different reproduction times of the specialists) in the sense that it was not observed previously, in the simulations of the preliminary model (where the two specialists’ reproduction times were fixed to be the same).

A particular equilibrium, when only the generalist survives, was exactly analyzed. Interestingly, the golden ratio arose in this equilibrium regarding the densities of the “young” and “old” protocells.

Regarding the application possibilities in the practice, at the population level, the proposed (extended) model may serve as a modeling tool in bio- and agricultural engineering (e.g. in the field of biological pest control in particular ecosystems, e.g. such as greenhouses). In particular, based on our dynamic model, control and observation systems could be set up, see e.g. (Gámez et al. 2017). The main challenge will be that unlike the quoted papers dealing with continuous-time model, in the present case we have discrete-time dynamics.

We think that in further research in the future, the worked out extended model (with simple algebraic relations) may serve as a simple useful tool for studying more life-like phenomena in ecosystems in their pure/most direct form. It is also worth continuing with the analytical study of the dynamic model, regarding further equilibria (besides the one analyzed in the previous section), finding the Lyapunov spectrum, bifurcations, periodic or quasi-periodic orbits, (chaotic) attractors etc., and their possible biological interpretations. Furthermore, the model may be extended to more than three species and nutrients.

Our paper analyzes the interaction of several populations. In a next study, a discrete-time control system is to be investigated, where the control function is the release of individuals of one of the populations. This model will be analog to those used in modeling of biological pest control.

Finally, the predictions provided by the extended model, especially in relation to the discovered “anomaly”, might be validated experimentally, based on real (measured) systems of populations.

## Supplementary Information

Below is the link to the electronic supplementary material.

Supplementary file1 (PDF 761 KB)

## Data Availability

The paper has no associated data.
