# Supplementary material for: Extended Discrete-Time Population Model to Describe the Competition of Nutrient-Producing Protocells
Source: Bull Math Biol. 2025 Jul 18;87(8):111. doi: 10.1007/s11538-025-01488-0 (PMC12274253; doi:10.1007/s11538-025-01488-0)
Supplement: Supplementary file 1 — Supplementary file1 (PDF 761 KB) [file 11538_2025_1488_MOESM1_ESM.pdf]

Appendix

for the paper

"Extended discrete-time population model to describe the competition of nutrient-producing protocells"

The following abbreviating symbols for the sum of different densities of young and old protocells (defined in the paper as well) are used at some places in this Appendix:

$QT_0 := QT_{0,1} + QT_{0,2}$, $QT_1 := QT_{1,1} + QT_{1,2}$, $QT_2 := QT_{2,1} + QT_{2,2}$,

$Q\tilde{T} := Q\tilde{T}_{0,1} + Q\tilde{T}_{0,2} + Q\tilde{T}_{1,1} + Q\tilde{T}_{1,2} + Q\tilde{T}_{2,1} + Q\tilde{T}_{2,2}$,

$Q\tilde{T}_0 := Q\tilde{T}_{0,1} + Q\tilde{T}_{0,2}$, $Q\tilde{T}_1 := Q\tilde{T}_{1,1} + Q\tilde{T}_{1,2}$, $Q\tilde{T}_2 := Q\tilde{T}_{2,1} + Q\tilde{T}_{2,2}$.

A. Supplementary relations for the operation cases of the extended model

II. $t_i \neq h_{\tau_0}(i)$ ($t_i > h_{\tau_0}(i)$ or $h_{\tau_0}(i)$ is not defined) and $t_i = h_{\tau_1}(i)$ and $t_i \neq h_{\tau_2}(i)$ ($t_i > h_{\tau_2}(i)$ or $h_{\tau_2}(i)$ is not defined)

In this case, t_i is an operation time point of T_1 and not an operation time point of T_0 and T_2.

II.1. Densities just before the competition

This sub-case corresponds to sub-figure *a*) in Fig. 2.

II.1/A) $t_i = 0$

Different densities are not to be determined in this case. Go to the determination of the densities just after the competition, that is, to sub-case II.2 below.

II.1/B) $t_i > 0$

$Q\tilde{T}_{0,1}(t_i) = QT_{0,1}(t_{i-1})$,

$Q\tilde{T}_{0,2}(t_i) = QT_{0,2}(t_{i-1})$,

$Q\tilde{T}_{2,1}(t_i) = QT_{2,1}(t_{i-1})$,

$Q\tilde{T}_{2,2}(t_i) = QT_{2,2}(t_{i-1})$,

$Qx(t_i) = max\big(Qx_f(t_{i-1}) + Qx_h(t_{i-1}) + b(t_i - t_{i-1}), 0\big)$.

II.1/B/a) $t_i = t_{1,0}$

In this case, t_i is the initial operation time point of T_1.

$Qx_1(t_i) = max\big(Qx_{1,f}(t_{i-1}) + Qx_{1,h}(t_{i-1}) + b_1(t_i - t_{i-1}), 0\big)$,

$Qx_2(t_i) = max\big(Qx_{2,f}(t_{i-1}) + Qx_{2,h}(t_{i-1}) + b_2(t_i - t_{i-1}), 0\big)$.

II.1/B/b) $t_i > t_{1,0}$

In this case, t_i is not the initial operation time point of T_1 (yet).

$$Q\tilde{T}_{1,1}(t_i) = QT_1\left(h_{\tau_1}(i-1)\right),$$

$$Q\tilde{T}_{1,2}(t_i) = QT_{1,1}\left(h_{\tau_1}(i-1)\right),$$

$$Qx_1(t_i) = max\left(Qx_{1,f}(t_{i-1}) + Qx_{1,h}(t_{i-1}) + b_1(t_i - t_{i-1}) + QT_1\left(h_{\tau_1}(i-1)\right), 0\right),$$

$$Qx_2(t_i) = max\big(Qx_{2,f}(t_{i-1}) + Qx_{2,h}(t_{i-1}) + b_2(t_i - t_{i-1}), 0\big).$$

II.2. Densities just after the competition

This sub-case corresponds to sub-figure *b*) in Fig. 2.

Determination of the density of the $T_{1,1}$-type protocells surviving the competition ($QT_{1,1}(t_i)$)

This density ($QT_{1,1,\mathrm{x}}(t_\mathrm{i})$) is that of the $T_{1,1}$-type protocells which get nutrient x (protocells of density $Q\tilde{T}_1(t_\mathrm{i})$ compete for x of density $Qx(t_\mathrm{i})$):

If $Q\tilde{T}_{1,1}(t_\mathrm{i}) = 0$ or $Qx(t_\mathrm{i}) = 0$, then

$QT_{1,1,\mathrm{x}}(t_\mathrm{i}) = 0,$

otherwise (computing with expected value, similarly as in Case I, in the determination of $QT_{0,1}(t_\mathrm{i})$),

$$QT_{1,1,\mathrm{x}}(t_\mathrm{i}) = min\left(\frac{Q\tilde{T}_{1,1}(t_\mathrm{i})Qx(t_\mathrm{i})}{Q\tilde{T}_1(t_\mathrm{i})}, Q\tilde{T}_{1,1}(t_\mathrm{i})\right).$$

This density ($QT_{1,1}(t_\mathrm{i})$) is that of the $T_{1,1}$-type protocells which get both nutrients x and x_2 (protocells of density $Q\tilde{T}_1(t_\mathrm{i})$ compete for x_2 of density $Qx_2(t_\mathrm{i})$):

If $QT_{1,1,\mathrm{x}}(t_\mathrm{i}) = 0$ or $Qx_2(t_\mathrm{i}) = 0$, then

$QT_{1,1}(t_\mathrm{i}) = 0,$

otherwise (computing with expected value),

$$QT_{1,1}(t_\mathrm{i}) = min\left(\frac{QT_{1,1,\mathrm{x}}(t_\mathrm{i})Qx_2(t_\mathrm{i})}{Q\tilde{T}_1(t_\mathrm{i})}, QT_{1,1,\mathrm{x}}(t_\mathrm{i})\right).$$

Determination of the density of the $T_{1,2}$-type protocells surviving the competition ($QT_{1,2}(t_\mathrm{i})$)

This density ($QT_{1,2,\mathrm{x}}(t_\mathrm{i})$) is that of the $T_{1,2}$-type protocells which get nutrient x (there is still $Qx(t_\mathrm{i}) - QT_{1,1,\mathrm{x}}(t_\mathrm{i})$ of x, which is completely left for them, that is, for the corresponding $T_{1,2}$-type protocells):

$$QT_{1,2,\mathrm{x}}(t_\mathrm{i}) = min\left(Qx(t_\mathrm{i}) - QT_{1,1,\mathrm{x}}(t_i), Q\tilde{T}_{1,2}(t_\mathrm{i})\right).$$

This density ($QT_{1,2,\mathrm{x},\mathrm{x1}}(t_\mathrm{i})$) is that of the $T_{1,2}$-type protocells which get x and x_1 (protocells of density $Q\tilde{T}_{1,2}(t_\mathrm{i})$ compete for x_1 of density $Qx_1(t_\mathrm{i})$):

If $QT_{1,2,\mathrm{x}}(t_\mathrm{i}) = 0$ or $Qx_1(t_\mathrm{i}) = 0$, then

$QT_{1,2,\mathrm{x},\mathrm{x1}}(t_\mathrm{i}) = 0,$

otherwise,

$$QT_{1,2,\mathrm{x},\mathrm{x1}}(t_\mathrm{i}) = min\left(\frac{QT_{1,2,\mathrm{x}}(t_\mathrm{i})Qx_1(t_\mathrm{i})}{Q\tilde{T}_{1,2}(t_\mathrm{i})}, QT_{1,2,\mathrm{x}}(t_\mathrm{i})\right).$$

This density ($QT_{1,2}(t_\mathrm{i})$) is that of the $T_{1,2}$-type protocells which get x, x_1 and x_2 (protocells of density $Q\tilde{T}_1(t_\mathrm{i}) - QT_{1,1,\mathrm{x}}(t_\mathrm{i})$ compete for the still free x_2 of density $Qx_2(t_\mathrm{i}) - QT_{1,1}(t_\mathrm{i})$):

If $QT_{1,2,\mathrm{x},\mathrm{x1}}(t_\mathrm{i}) = 0$ or $Qx_2(t_\mathrm{i}) - QT_{1,1}(t_\mathrm{i}) = 0$, then

$QT_{1,2}(t_\mathrm{i}) = 0,$

otherwise,

$$QT_{1,2}(t_\mathrm{i}) = min\left(\frac{QT_{1,2,\mathrm{x},\mathrm{x1}}(t_\mathrm{i})\left(Qx_2(t_\mathrm{i}) - QT_{1,1}(t_\mathrm{i})\right)}{Q\tilde{T}_1(t_\mathrm{i}) - QT_{1,1,\mathrm{x}}(t_\mathrm{i})}, QT_{1,2,\mathrm{x},\mathrm{x1}}(t_\mathrm{i})\right).$$

This density of x is not absorbed by the protocells in the competition (surplus of x):

$$Qx_\mathrm{f}(t_\mathrm{i}) = max\left(Qx(t_\mathrm{i}) - Q\tilde{T}_1(t_\mathrm{i}), 0\right).$$

This is the density of nutrient x absorbed by the not surviving protocells (which go extinct by the next modeling time point t_{i+1}) and, consequently, get out to the environment by the next modeling time point t_{i+1}:

$$Qx_h(t_i) = min\left(Qx(t_i), Q\tilde{T}_1(t_i)\right) - QT_1(t_i).$$

This density of x_1 is not absorbed by the protocells in the competition (surplus of x_1):

$$Qx_{1,f}(t_i) = max\left(Qx_1(t_i) - Q\tilde{T}_{1,2}(t_i), 0\right).$$

This is the density of nutrient x_1 absorbed by the not surviving protocells and, consequently, get out to the environment by the next modeling time point:

$$Qx_{1,h}(t_i) = min\left(Qx_1(t_i), Q\tilde{T}_{1,2}(t_i)\right) - QT_{1,2}(t_i).$$

This density of x_2 is not absorbed by the protocells in the competition (surplus of x_2):

$$Qx_{2,f}(t_i) = max\left(Qx_2(t_i) - Q\tilde{T}_1(t_i), 0\right).$$

This is the density of nutrient x_2 absorbed by the not surviving protocells and, consequently, get out to the environment by the next modeling time point:

$$Qx_{2,h}(t_i) = min\left(Qx_2(t_i), Q\tilde{T}_1(t_i)\right) - QT_1(t_i).$$

The densities of those protocell types are not changed for which the current modeling time point (t_i) is not an operation time point:

$$QT_{0,1}(t_i) = Q\tilde{T}_{0,1}(t_i),$$

$$QT_{0,2}(t_i) = Q\tilde{T}_{0,2}(t_i),$$

$$QT_{2,1}(t_i) = Q\tilde{T}_{2,1}(t_i),$$

$$QT_{2,2}(t_i) = Q\tilde{T}_{2,2}(t_i).$$

<u>III.</u> $t_i \neq h_{\tau_0}(i)$ ($t_i > h_{\tau_0}(i)$ or $h_{\tau_0}(i)$ is not defined) and $t_i \neq h_{\tau_1}(i)$ ($t_i > h_{\tau_1}(i)$ or $h_{\tau_1}(i)$ is not defined) and $t_i = h_{\tau_2}(i)$

In this case t_i is an operation time point of T_2 and not an operation time point of T_0 and T_1.

III.1. Densities just before the competition

This sub-case corresponds to sub-figure *a*) in Fig. 2.

III.1/A) $t_i = 0$

Different densities are not to be determined in this case. Go to the determination of the densities just after the competition, that is, to sub-case III.2 below.

III.1/B) $t_i > 0$

$$Q\tilde{T}_{0,1}(t_i) = QT_{0,1}(t_{i-1}),$$

$$Q\tilde{T}_{0,2}(t_i) = QT_{0,2}(t_{i-1}),$$

$$Q\tilde{T}_{1,1}(t_i) = QT_{1,1}(t_{i-1}),$$

$$Q\tilde{T}_{1,2}(t_i) = QT_{1,2}(t_{i-1}),$$

$$Qx(t_i) = max(Qx_f(t_{i-1}) + Qx_h(t_{i-1}) + b(t_i - t_{i-1}), 0).$$

III.1/B/a) $t_i = t_{2,0}$

In this case, t_i is the initial operation time point of T_2.

$$Qx_1(t_i) = max\left(Qx_{1,f}(t_{i-1}) + Qx_{1,h}(t_{i-1}) + b_1(t_i - t_{i-1}), 0\right),$$

$Qx_2(t_\mathrm{i}) = max\big(Qx_{2,\mathrm{f}}(t_{\mathrm{i}-1}) + Qx_{2,\mathrm{h}}(t_{\mathrm{i}-1}) + b_2(t_\mathrm{i} - t_{\mathrm{i}-1}), 0\big).$

III.1/B/b) $\boldsymbol{t_\mathrm{i} > t_{2,0}}$

In this case, t_i is not the initial operation time point of T_2 (yet).

$Q\tilde{T}_{2,1}(t_\mathrm{i}) = QT_2\left(h_{\tau_2}(i-1)\right),$

$Q\tilde{T}_{2,2}(t_\mathrm{i}) = QT_{2,1}\left(h_{\tau_2}(i-1)\right),$

$Qx_1(t_\mathrm{i}) = max\big(Qx_{1,\mathrm{f}}(t_{\mathrm{i}-1}) + Qx_{1,\mathrm{h}}(t_{\mathrm{i}-1}) + b_1(t_\mathrm{i} - t_{\mathrm{i}-1}), 0\big),$

$Qx_2(t_\mathrm{i}) = max\left(Qx_{2,\mathrm{f}}(t_{\mathrm{i}-1}) + Qx_{2,\mathrm{h}}(t_{\mathrm{i}-1}) + b_2(t_\mathrm{i} - t_{\mathrm{i}-1}) + QT_2\left(h_{\tau_2}(i-1)\right), 0\right).$

III.2. Densities just after the competition

This sub-case corresponds to sub-figure *b*) in Fig. 2.

Determination of the density of the $T_{2,1}$-type protocells surviving the competition ($QT_{2,1}(t_\mathrm{i})$)

This density ($QT_{2,1,\mathrm{x}}(t_\mathrm{i})$) is that of the $T_{2,1}$-type protocells which get x (protocells of density $Q\tilde{T}_2(t_\mathrm{i})$ compete for x of density $Qx(t_\mathrm{i})$):

If $Q\tilde{T}_{2,1}(t_\mathrm{i}) = 0$ or $Qx(t_\mathrm{i}) = 0$, then

$QT_{2,1,\mathrm{x}}(t_\mathrm{i}) = 0,$

otherwise,

$QT_{2,1,\mathrm{x}}(t_\mathrm{i}) = min\left(\frac{Q\tilde{T}_{2,1}(t_\mathrm{i})Qx(t_\mathrm{i})}{Q\tilde{T}_2(t_\mathrm{i})}, Q\tilde{T}_{2,1}(t_\mathrm{i})\right).$

This density ($QT_{2,1}(t_\mathrm{i})$) is that of the $T_{2,1}$-type protocells which get x and x_1 (protocells of density $Q\tilde{T}_2(t_\mathrm{i})$ compete for x_1 of density $Qx_1(t_\mathrm{i})$):

If $QT_{2,1,\mathrm{x}}(t_\mathrm{i}) = 0$ or $Qx_1(t_\mathrm{i}) = 0$, then

$QT_{2,1}(t_\mathrm{i}) = 0,$

otherwise,

$QT_{2,1}(t_\mathrm{i}) = min\left(\frac{QT_{2,1,\mathrm{x}}(t_\mathrm{i})Qx_1(t_\mathrm{i})}{Q\tilde{T}_2(t_\mathrm{i})}, QT_{2,1,\mathrm{x}}(t_\mathrm{i})\right).$

Determination of the density of the $T_{2,2}$-type protocells surviving the competition ($QT_{2,2}(t_\mathrm{i})$)

This density ($QT_{2,2,\mathrm{x}}(t_\mathrm{i})$) is that of the $T_{2,2}$-type protocells which get nutrient x (there is still $Qx(t_\mathrm{i}) - QT_{2,1,\mathrm{x}}(t_\mathrm{i})$ of x, which is completely left for them):

$QT_{2,2,\mathrm{x}}(t_\mathrm{i}) = min\left(Qx(t_\mathrm{i}) - QT_{2,1,\mathrm{x}}(t_\mathrm{i}), Q\tilde{T}_{2,2}(t_\mathrm{i})\right).$

This density ($QT_{2,2,\mathrm{x,x1}}(t_\mathrm{i})$) is that of the $T_{2,2}$-type protocells which get x and x_1 (protocells of density $Q\tilde{T}_2(t_\mathrm{i}) - QT_{2,1,\mathrm{x}}(t_\mathrm{i})$ compete for x_1 of density $Qx_1(t_\mathrm{i}) - QT_{2,1}(t_\mathrm{i})$):

If $QT_{2,2,\mathrm{x}}(t_\mathrm{i}) = 0$ or $Qx_1(t_\mathrm{i}) - QT_{2,1}(t_\mathrm{i}) = 0$, then

$QT_{2,2,\mathrm{x,x1}}(t_\mathrm{i}) = 0,$

otherwise,

$QT_{2,2,\mathrm{x,x1}}(t_\mathrm{i}) = min\left(\frac{QT_{2,2,\mathrm{x}}(t_\mathrm{i})\big(Qx_1(t_\mathrm{i}) - QT_{2,1}(t_\mathrm{i})\big)}{Q\tilde{T}_2(t_\mathrm{i}) - QT_{2,1,\mathrm{x}}(t_i)}, QT_{2,2,\mathrm{x}}(t_\mathrm{i})\right).$

This density ($QT_{2,2}(t_{\mathrm{i}})$) is that of the $T_{2,2}$-type protocells which get x, x_1 and x_2 (protocells of density $Q\tilde{T}_{2,2}(t_{\mathrm{i}})$ compete for x_2 of density $Qx_2(t_{\mathrm{i}})$):

If $QT_{2,2,x,x1}(t_{\mathrm{i}}) = 0$ or $Qx_2(t_{\mathrm{i}}) = 0$, then

$QT_{2,2}(t_{\mathrm{i}}) = 0$,

otherwise,

$$QT_{2,2}(t_{\mathrm{i}}) = min\left(\frac{QT_{2,2,\mathrm{x},\mathrm{x}1}(t_{\mathrm{i}})Qx_2(t_{\mathrm{i}})}{Q\tilde{T}_{2,2}(t_{\mathrm{i}})}, QT_{2,2,\mathrm{x},\mathrm{x}1}(t_{\mathrm{i}})\right).$$

This density of x is not absorbed by the protocells in the competition (surplus of x):

$$Qx_{\mathrm{f}}(t_{\mathrm{i}}) = max\big(Qx(t_{\mathrm{i}}) - Q\tilde{T}_2(t_{\mathrm{i}}), 0\big).$$

This is the density of x absorbed by the not surviving protocells and, consequently, get out to the environment by the next modeling time point:

$$Qx_{\mathrm{h}}(t_{\mathrm{i}}) = min\left(Qx(t_{\mathrm{i}}), Q\tilde{T}_2(t_{\mathrm{i}})\right) - QT_2(t_{\mathrm{i}}).$$

This density of x_1 is not absorbed by the protocells in the competition (surplus of x_1):

$$Qx_{1,\mathrm{f}}(t_{\mathrm{i}}) = max\big(Qx_1(t_{\mathrm{i}}) - Q\tilde{T}_2(t_{\mathrm{i}}), 0\big).$$

This is the density of x_1 absorbed by the not surviving protocells and, consequently, get out to the environment by the next modeling time point:

$$Qx_{1,\mathrm{h}}(t_{\mathrm{i}}) = min\left(Qx_1(t_{\mathrm{i}}), Q\tilde{T}_2(t_{\mathrm{i}})\right) - QT_2(t_{\mathrm{i}}).$$

This density of x_2 is not absorbed by the protocells in the competition (surplus of x_2):

$$Qx_{2,\mathrm{f}}(t_{\mathrm{i}}) = max\big(Qx_2(t_{\mathrm{i}}) - Q\tilde{T}_{2,2}(t_{\mathrm{i}}), 0\big).$$

This is the density of x_2 absorbed by the not surviving protocells and, consequently, get out to the environment by the next modeling time point:

$$Qx_{2,\mathrm{h}}(t_{\mathrm{i}}) = min\left(Qx_2(t_{\mathrm{i}}), Q\tilde{T}_{2,2}(t_{\mathrm{i}})\right) - QT_{2,2}(t_{\mathrm{i}}).$$

The densities of those protocell types are not changed for which the current modeling time point (t_{i}) is not an operation time point:

$QT_{0,1}(t_{\mathrm{i}}) = Q\tilde{T}_{0,1}(t_{\mathrm{i}})$,

$QT_{0,2}(t_{\mathrm{i}}) = Q\tilde{T}_{0,2}(t_{\mathrm{i}})$,

$QT_{1,1}(t_{\mathrm{i}}) = Q\tilde{T}_{1,1}(t_{\mathrm{i}})$,

$QT_{1,2}(t_{\mathrm{i}}) = Q\tilde{T}_{1,2}(t_{\mathrm{i}})$.

IV. $t_{\mathrm{i}} = h_{\tau_0}(i)$ and $t_{\mathrm{i}} = h_{\tau_1}(i)$ and $t_{\mathrm{i}} \neq h_{\tau_2}(i)$ ($t_{\mathrm{i}} > h_{\tau_2}(i)$ or $h_{\tau_2}(i)$ is not defined)

In this case, t_{i} is an operation time point of T_0 and T_1 and not an operation time point of T_2.

IV.1. Densities just before the competition

This sub-case corresponds to sub-figure *a*) in Fig. 2.

IV.1/A) $t_{\mathrm{i}} = 0$

Different densities are not to be determined in this case. Go to the determination of the densities just after the competition, that is, to sub-case IV.2 below.

IV.1/B) $t_{\mathrm{i}} > 0$

$Q\tilde{T}_{2,1}(t_{\mathrm{i}}) = QT_{2,1}(t_{\mathrm{i}-1})$,

$Q\tilde{T}_{2,2}(t_{\mathrm{i}}) = QT_{2,2}(t_{\mathrm{i-1}})$,

$Qx(t_{\mathrm{i}}) = max(Qx_{\mathrm{f}}(t_{\mathrm{i-1}}) + Qx_{\mathrm{h}}(t_{\mathrm{i-1}}) + b(t_{\mathrm{i}} - t_{\mathrm{i-1}}), 0)$.

IV.1/B/a) $\boldsymbol{t_{\mathrm{i}} = t_{0,0}}$ ***and*** $\boldsymbol{t_{\mathrm{i}} = t_{1,0}}$

In this case, t_{i} is the initial operation time point of T_0 and T_1.

$Qx_1(t_{\mathrm{i}}) = max\big(Qx_{1,\mathrm{f}}(t_{\mathrm{i-1}}) + Qx_{1,\mathrm{h}}(t_{\mathrm{i-1}}) + b_1(t_{\mathrm{i}} - t_{\mathrm{i-1}}), 0\big)$,

$Qx_2(t_{\mathrm{i}}) = max\big(Qx_{2,\mathrm{f}}(t_{\mathrm{i-1}}) + Qx_{2,\mathrm{h}}(t_{\mathrm{i-1}}) + b_2(t_{\mathrm{i}} - t_{\mathrm{i-1}}), 0\big)$.

IV.1/B/b) $\boldsymbol{t_{\mathrm{i}} = t_{0,0}}$ ***and*** $\boldsymbol{t_{\mathrm{i}} > t_{1,0}}$

In this case, t_{i} is the initial operation time point of T_0 but not the initial operation time point of T_1 (yet).

$Q\tilde{T}_{1,1}(t_{\mathrm{i}}) = QT_1\left(h_{\tau_1}(i-1)\right)$,

$Q\tilde{T}_{1,2}(t_{\mathrm{i}}) = QT_{1,1}\left(h_{\tau_1}(i-1)\right)$,

$Qx_1(t_{\mathrm{i}}) = max\left(Qx_{1,\mathrm{f}}(t_{\mathrm{i-1}}) + Qx_{1,\mathrm{h}}(t_{\mathrm{i-1}}) + b_1(t_{\mathrm{i}} - t_{\mathrm{i-1}}) + QT_1\left(h_{\tau_1}(i-1)\right), 0\right)$,

$Qx_2(t_{\mathrm{i}}) = max\big(Qx_{2,\mathrm{f}}(t_{\mathrm{i-1}}) + Qx_{2,\mathrm{h}}(t_{\mathrm{i-1}}) + b_2(t_{\mathrm{i}} - t_{\mathrm{i-1}}), 0\big)$.

IV.1/B/c) $\boldsymbol{t_{\mathrm{i}} > t_{0,0}}$ ***and*** $\boldsymbol{t_{\mathrm{i}} = t_{1,0}}$

In this case, t_{i} is not the initial operation time point of T_0 (yet) but the initial operation time point of T_1.

$Q\tilde{T}_{0,1}(t_{\mathrm{i}}) = QT_0\left(h_{\tau_0}(i-1)\right)$,

$Q\tilde{T}_{0,2}(t_{\mathrm{i}}) = QT_{0,1}\left(h_{\tau_0}(i-1)\right)$,

$Qx_1(t_{\mathrm{i}}) = max\left(Qx_{1,\mathrm{f}}(t_{\mathrm{i-1}}) + Qx_{1,\mathrm{h}}(t_{\mathrm{i-1}}) + b_1(t_{\mathrm{i}} - t_{\mathrm{i-1}}) + QT_0\left(h_{\tau_0}(i-1)\right), 0\right)$,

$Qx_2(t_{\mathrm{i}}) = max\left(Qx_{2,\mathrm{f}}(t_{\mathrm{i-1}}) + Qx_{2,\mathrm{h}}(t_{\mathrm{i-1}}) + b_2(t_{\mathrm{i}} - t_{\mathrm{i-1}}) + QT_0\left(h_{\tau_0}(i-1)\right), 0\right)$.

IV.1/B/d) $\boldsymbol{t_{\mathrm{i}} > t_{0,0}}$ ***and*** $\boldsymbol{t_{\mathrm{i}} > t_{1,0}}$

In this case, t_{i} is not the initial operation time point of T_0 and T_1 (yet).

$Q\tilde{T}_{0,1}(t_{\mathrm{i}}) = QT_0\left(h_{\tau_0}(i-1)\right)$,

$Q\tilde{T}_{0,2}(t_{\mathrm{i}}) = QT_{0,1}\left(h_{\tau_0}(i-1)\right)$,

$Q\tilde{T}_{1,1}(t_{\mathrm{i}}) = QT_1\left(h_{\tau_1}(i-1)\right)$,

$Q\tilde{T}_{1,2}(t_{\mathrm{i}}) = QT_{1,1}\left(h_{\tau_1}(i-1)\right)$,

$Qx_1(t_{\mathrm{i}}) = max\left(Qx_{1,\mathrm{f}}(t_{\mathrm{i-1}}) + Qx_{1,\mathrm{h}}(t_{\mathrm{i-1}}) + b_1(t_{\mathrm{i}} - t_{\mathrm{i-1}}) + QT_0\left(h_{\tau_0}(i-1)\right) + \right.$
$\left. QT_1\left(h_{\tau_1}(i-1)\right), 0\right)$,

$Qx_2(t_{\mathrm{i}}) = max\left(Qx_{2,\mathrm{f}}(t_{\mathrm{i-1}}) + Qx_{2,\mathrm{h}}(t_{\mathrm{i-1}}) + b_2(t_{\mathrm{i}} - t_{\mathrm{i-1}}) + QT_0\left(h_{\tau_0}(i-1)\right), 0\right)$.

IV.2. Densities just after the competition

This sub-case corresponds to sub-figure *b*) in Fig. 2.

Determination of the density of the $T_{0,1}$*-type protocells surviving the competition (*$QT_{0,1}(t_{\mathrm{i}})$*)*

This density ($QT_{0,1}(t_i)$) is that of the $T_{0,1}$-type protocells which get x (protocells of density $Q\tilde{T}_0(t_i) + Q\tilde{T}_1(t_i)$ compete for x of density $Qx(t_i)$):

If $Q\tilde{T}_{0,1}(t_i) = 0$ or $Qx(t_i) = 0$, then

$QT_{0,1}(t_i) = 0,$

otherwise,

$$QT_{0,1}(t_i) = min\left(\frac{Q\tilde{T}_{0,1}(t_i)Qx(t_i)}{Q\tilde{T}_0(t_i)+Q\tilde{T}_1(t_i)}, Q\tilde{T}_{0,1}(t_i)\right).$$

Determination of the density of the $T_{0,2}$-type protocells surviving the competition ($QT_{0,2}(t_i)$)

This density ($QT_{0,2,x}(t_i)$) is that of the $T_{0,2}$-type protocells which get x (protocells of density $Q\tilde{T}_{0,2}(t_i) + Q\tilde{T}_1(t_i)$ compete for x of density $Qx(t_i) - QT_{0,1}(t_i)$):

If $Q\tilde{T}_{0,2}(t_i) = 0$ or $Qx(t_i) - QT_{0,1}(t_i) = 0$, then

$QT_{0,2,x}(t_i) = 0,$

otherwise,

$$QT_{0,2,x}(t_i) = min\left(\frac{Q\tilde{T}_{0,2}(t_i)\left(Qx(t_i)-QT_{0,1}(t_i)\right)}{Q\tilde{T}_{0,2}(t_i)+Q\tilde{T}_1(t_i)}, Q\tilde{T}_{0,2}(t_i)\right).$$

This density ($QT_{0,2,x,x1}(t_i)$) is that of the $T_{0,2}$-type protocells which get x and x_1 (protocells of density $Q\tilde{T}_{0,2}(t_i) + Q\tilde{T}_{1,2}(t_i)$ compete for x_1 of density $Qx_1(t_i)$):

If $QT_{0,2,x}(t_i) = 0$ or $Qx_1(t_i) = 0$, then

$QT_{0,2,x,x1}(t_i) = 0,$

otherwise,

$$QT_{0,2,x,x1}(t_i) = min\left(\frac{QT_{0,2,x}(t_i)Qx_1(t_i)}{Q\tilde{T}_{0,2}(t_i)+Q\tilde{T}_{1,2}(t_i)}, QT_{0,2,x}(t_i)\right).$$

This density ($QT_{0,2}(t_i)$) is that of the $T_{0,2}$-type protocells which get x, x_1 and x_2 (protocells of density $Q\tilde{T}_{0,2}(t_i) + Q\tilde{T}_1(t_i)$ compete for x_2 of density $Qx_2(t_i)$):

If $QT_{0,2,x,x1}(t_i) = 0$ or $Qx_2(t_i) = 0$, then

$QT_{0,2}(t_i) = 0,$

otherwise,

$$QT_{0,2}(t_i) = min\left(\frac{QT_{0,2,x,x1}(t_i)Qx_2(t_i)}{Q\tilde{T}_{0,2}(t_i)+Q\tilde{T}_1(t_i)}, QT_{0,2,x,x1}(t_i)\right).$$

Determination of the density of the $T_{1,1}$-type protocells surviving the competition ($QT_{1,1}(t_i)$)

This density ($QT_{1,1,x}(t_i)$) is that of the $T_{1,1}$-type protocells which get x (protocells of density $Q\tilde{T}_1(t_i)$ compete for x of density $Qx(t_i) - QT_{0,1}(t_i) - QT_{0,2,x}(t_i)$):

If $Q\tilde{T}_{1,1}(t_i) = 0$ or $Qx(t_i) - QT_{0,1}(t_i) - QT_{0,2,x}(t_i) = 0$, then

$QT_{1,1,x}(t_i) = 0,$

otherwise,

$$QT_{1,1,x}(t_i) = min\left(\frac{Q\tilde{T}_{1,1}(t_i)\left(Qx(t_i)-QT_{0,1}(t_i)-QT_{0,2,x}(t_i)\right)}{Q\tilde{T}_1(t_i)}, Q\tilde{T}_{1,1}(t_i)\right).$$

This density ($QT_{1,1}(t_\mathrm{i})$) is that of the $T_{1,1}$-type protocells which get x and x_2 (protocells of density $Q\tilde{T}_{0,2}(t_\mathrm{i}) - QT_{0,2,\mathrm{x,x1}}(t_\mathrm{i}) + Q\tilde{T}_1(t_\mathrm{i})$ compete for x_2 of density $Qx_2(t_\mathrm{i}) - QT_{0,2}(t_\mathrm{i})$):

If $QT_{1,1,\mathrm{x}}(t_\mathrm{i}) = 0$ or $Qx_2(t_\mathrm{i}) - QT_{0,2}(t_\mathrm{i}) = 0$, then

$QT_{1,1}(t_\mathrm{i}) = 0$,

otherwise,

$$QT_{1,1}(t_\mathrm{i}) = min\left(\frac{QT_{1,1,\mathrm{x}}(t_\mathrm{i})\left(Qx_2(t_\mathrm{i}) - QT_{0,2}(t_\mathrm{i})\right)}{Q\tilde{T}_{0,2}(t_\mathrm{i}) - QT_{0,2,\mathrm{x,x1}}(t_\mathrm{i}) + Q\tilde{T}_1(t_\mathrm{i})}, QT_{1,1,\mathrm{x}}(t_\mathrm{i})\right).$$

Determination of the density of the $T_{1,2}$-type protocells surviving the competition ($QT_{1,2}(t_\mathrm{i})$)

This density ($QT_{1,2,\mathrm{x}}(t_\mathrm{i})$) is that of the $T_{1,2}$-type protocells which get nutrient x (there is still $Qx(t_\mathrm{i}) - QT_{0,1}(t_\mathrm{i}) - QT_{0,2,\mathrm{x}}(t_\mathrm{i}) - QT_{1,1,\mathrm{x}}(t_\mathrm{i})$ of x, which is completely left for them):

$$QT_{1,2,\mathrm{x}}(t_\mathrm{i}) = min\left(Qx(t_\mathrm{i}) - QT_{0,1}(t_\mathrm{i}) - QT_{0,2,\mathrm{x}}(t_\mathrm{i}) - QT_{1,1,\mathrm{x}}(t_\mathrm{i}), Q\tilde{T}_{1,2}(t_\mathrm{i})\right).$$

This density ($QT_{1,2,\mathrm{x,x1}}(t_\mathrm{i})$) is that of the $T_{1,2}$-type protocells which get x and x_1 (protocells of density $Q\tilde{T}_{0,2}(t_\mathrm{i}) - QT_{0,2,\mathrm{x}}(t_\mathrm{i}) + Q\tilde{T}_{1,2}(t_\mathrm{i})$ compete for x_1 of density $Qx_1(t_\mathrm{i}) - QT_{0,2,\mathrm{x,x1}}(t_\mathrm{i})$):

If $QT_{1,2,\mathrm{x}}(t_\mathrm{i}) = 0$ or $Qx_1(t_\mathrm{i}) - QT_{0,2,\mathrm{x,x1}}(t_\mathrm{i}) = 0$, then

$QT_{1,2,\mathrm{x,x1}}(t_\mathrm{i}) = 0$,

otherwise,

$$QT_{1,2,\mathrm{x,x1}}(t_\mathrm{i}) = min\left(\frac{QT_{1,2,\mathrm{x}}(t_\mathrm{i})\left(Qx_1(t_\mathrm{i}) - QT_{0,2,\mathrm{x,x1}}(t_\mathrm{i})\right)}{Q\tilde{T}_{0,2}(t_\mathrm{i}) - QT_{0,2,\mathrm{x}}(t_\mathrm{i}) + Q\tilde{T}_{1,2}(t_\mathrm{i})}, QT_{1,2,\mathrm{x}}(t_\mathrm{i})\right).$$

This density ($QT_{1,2}(t_\mathrm{i})$) is that of the $T_{1,2}$-type protocells which get x, x_1 and x_2 (protocells of density $Q\tilde{T}_{0,2}(t_\mathrm{i}) - QT_{0,2,\mathrm{x,x1}}(t_\mathrm{i}) + Q\tilde{T}_1(t_\mathrm{i}) - QT_{1,1,\mathrm{x}}(t_\mathrm{i})$ compete for x_2 of density $Qx_2(t_\mathrm{i}) - QT_{0,2}(t_\mathrm{i}) - QT_{1,1}(t_\mathrm{i})$):

If $QT_{1,2,\mathrm{x,x1}}(t_\mathrm{i}) = 0$ or $Qx_2(t_\mathrm{i}) - QT_{0,2}(t_\mathrm{i}) - QT_{1,1}(t_\mathrm{i}) = 0$, then

$QT_{1,2}(t_\mathrm{i}) = 0$,

otherwise,

$$QT_{1,2}(t_\mathrm{i}) = min\left(\frac{QT_{1,2,\mathrm{x,x1}}(t_\mathrm{i})\left(Qx_2(t_\mathrm{i}) - QT_{0,2}(t_\mathrm{i}) - QT_{1,1}(t_\mathrm{i})\right)}{Q\tilde{T}_{0,2}(t_\mathrm{i}) - QT_{0,2,\mathrm{x,x1}}(t_\mathrm{i}) + Q\tilde{T}_1(t_\mathrm{i}) - QT_{1,1,\mathrm{x}}(t_\mathrm{i})}, QT_{1,2,\mathrm{x,x1}}(t_\mathrm{i})\right).$$

This density of x is not absorbed by the protocells in the competition (surplus of x):

$$Qx_\mathrm{f}(t_\mathrm{i}) = max\left(Qx(t_\mathrm{i}) - Q\tilde{T}_0(t_\mathrm{i}) - Q\tilde{T}_1(t_\mathrm{i}), 0\right).$$

This is the density of x absorbed by the not surviving protocells and, consequently, get out to the environment by the next modeling time point:

$$Qx_\mathrm{h}(t_\mathrm{i}) = min\left(Qx(t_\mathrm{i}) - QT_{0,1}(t_\mathrm{i}), Q\tilde{T}_{0,2}(t_\mathrm{i}) + Q\tilde{T}_1(t_\mathrm{i})\right) - QT_{0,2}(t_\mathrm{i}) - QT_1(t_\mathrm{i}).$$

This density of x_1 is not absorbed by the protocells in the competition (surplus of x_1):

$$Qx_{1,\mathrm{f}}(t_\mathrm{i}) = max\left(Qx_1(t_\mathrm{i}) - Q\tilde{T}_{0,2}(t_\mathrm{i}) - Q\tilde{T}_{1,2}(t_\mathrm{i}), 0\right).$$

This is the density of x_1 absorbed by the not surviving protocells and, consequently, get out to the environment by the next modeling time point:

$Qx_{1,\mathrm{h}}(t_\mathrm{i}) = min\left(Qx_1(t_\mathrm{i}), Q\tilde{T}_{0,2}(t_\mathrm{i}) + Q\tilde{T}_{1,2}(t_\mathrm{i})\right) - QT_{0,2}(t_\mathrm{i}) - QT_{1,2}(t_\mathrm{i}).$

This density of x_2 is not absorbed by the protocells in the competition (surplus of x_2):

$Qx_{2,\mathrm{f}}(t_\mathrm{i}) = max\left(Qx_2(t_\mathrm{i}) - Q\tilde{T}_{0,2}(t_\mathrm{i}) - Q\tilde{T}_1(t_\mathrm{i}), 0\right).$

This is the density of x_2 absorbed by the not surviving protocells and, consequently, get out to the environment by the next modeling time point:

$Qx_{2,\mathrm{h}}(t_\mathrm{i}) = min\left(Qx_2(t_\mathrm{i}), Q\tilde{T}_{0,2}(t_\mathrm{i}) + Q\tilde{T}_1(t_\mathrm{i})\right) - QT_{0,2}(t_\mathrm{i}) - QT_1(t_\mathrm{i}).$

The densities of those protocell types are not changed for which the current modeling time point (t_i) is not an operation time point:

$QT_{2,1}(t_\mathrm{i}) = Q\tilde{T}_{2,1}(t_\mathrm{i}),$

$QT_{2,2}(t_\mathrm{i}) = Q\tilde{T}_{2,2}(t_\mathrm{i}).$

V. $t_\mathrm{i} = h_{\tau_0}(i)$ and $t_\mathrm{i} \neq h_{\tau_1}(i)$ ($t_\mathrm{i} > h_{\tau_1}(i)$ or $h_{\tau_1}(i)$ is not defined) and $t_\mathrm{i} = h_{\tau_2}(i)$
In this case, t_i is an operation time point of T_0 and T_2 but not an operation time point of T_1.

V.1. Densities just before the competition

This sub-case corresponds to sub-figure *a*) in Fig. 2.

V.1/A) $t_\mathrm{i} = 0$
Different densities are not to be determined in this case. Go to the determination of the densities just after the competition, that is, to sub-case V.2 below.

V.1/B) $t_\mathrm{i} > 0$

$Q\tilde{T}_{1,1}(t_\mathrm{i}) = QT_{1,1}(t_{\mathrm{i}-1}),$

$Q\tilde{T}_{1,2}(t_\mathrm{i}) = QT_{1,2}(t_{\mathrm{i}-1}),$

$Qx(t_\mathrm{i}) = max(Qx_\mathrm{f}(t_{\mathrm{i}-1}) + Qx_\mathrm{h}(t_{\mathrm{i}-1}) + b(t_\mathrm{i} - t_{\mathrm{i}-1}), 0).$

V.1/B/a) $t_\mathrm{i} = t_{0,0}$ and $t_\mathrm{i} = t_{2,0}$
In this case, t_i is the initial operation time point of T_0 and T_2.

$Qx_1(t_\mathrm{i}) = max\left(Qx_{1,\mathrm{f}}(t_{\mathrm{i}-1}) + Qx_{1,\mathrm{h}}(t_{\mathrm{i}-1}) + b_1(t_\mathrm{i} - t_{\mathrm{i}-1}), 0\right),$

$Qx_2(t_\mathrm{i}) = max\left(Qx_{2,\mathrm{f}}(t_{\mathrm{i}-1}) + Qx_{2,\mathrm{h}}(t_{\mathrm{i}-1}) + b_2(t_\mathrm{i} - t_{\mathrm{i}-1}), 0\right).$

V.1/B/b) $t_\mathrm{i} = t_{0,0}$ and $t_\mathrm{i} > t_{2,0}$
In this case, t_i is the initial operation time point of T_0 but not the initial operation time point of T_2 (yet).

$Q\tilde{T}_{2,1}(t_\mathrm{i}) = QT_2\left(h_{\tau_2}(i-1)\right),$

$Q\tilde{T}_{2,2}(t_\mathrm{i}) = QT_{2,1}\left(h_{\tau_2}(i-1)\right),$

$Qx_1(t_\mathrm{i}) = max\left(Qx_{1,\mathrm{f}}(t_{\mathrm{i}-1}) + Qx_{1,\mathrm{h}}(t_{\mathrm{i}-1}) + b_1(t_\mathrm{i} - t_{\mathrm{i}-1}), 0\right),$

$Qx_2(t_\mathrm{i}) = max\left(Qx_{2,\mathrm{f}}(t_{\mathrm{i}-1}) + Qx_{2,\mathrm{h}}(t_{\mathrm{i}-1}) + b_2(t_\mathrm{i} - t_{\mathrm{i}-1}) + QT_{2,1}\left(h_{\tau_2}(i-1)\right) + \right.$
$\left. QT_{2,2}\left(h_{\tau_2}(i-1)\right), 0\right).$

V.1/B/c) $t_\mathrm{i} > t_{0,0}$ and $t_\mathrm{i} = t_{2,0}$
In this case, t_i is not the initial operation time point of T_0 (yet) but the initial operation time point of T_2.

$Q\tilde{T}_{0,1}(t_i) = QT_0\left(h_{\tau_0}(i-1)\right)$,

$Q\tilde{T}_{0,2}(t_i) = QT_{0,1}\left(h_{\tau_0}(i-1)\right)$,

$Qx_1(t_i) = max\left(Qx_{1,f}(t_{i-1}) + Qx_{1,h}(t_{i-1}) + b_1(t_i - t_{i-1}) + QT_0\left(h_{\tau_0}(i-1)\right), 0\right)$,

$Qx_2(t_i) = max\left(Qx_{2,f}(t_{i-1}) + Qx_{2,h}(t_{i-1}) + b_2(t_i - t_{i-1}) + QT_0\left(h_{\tau_0}(i-1)\right), 0\right)$.

V.1/B/d) $\boldsymbol{t_i > t_{0,0}}$ ***and*** $\boldsymbol{t_i > t_{2,0}}$

In this case, t_i is not the initial operation time point of T_0 and T_2 (yet).

$Q\tilde{T}_{0,1}(t_i) = QT_0\left(h_{\tau_0}(i-1)\right)$,

$Q\tilde{T}_{0,2}(t_i) = QT_{0,1}\left(h_{\tau_0}(i-1)\right)$,

$Q\tilde{T}_{2,1}(t_i) = QT_2\left(h_{\tau_2}(i-1)\right)$,

$Q\tilde{T}_{2,2}(t_i) = QT_{2,1}\left(h_{\tau_2}(i-1)\right)$,

$Qx_1(t_i) = max\left(Qx_{1,f}(t_{i-1}) + Qx_{1,h}(t_{i-1}) + b_1(t_i - t_{i-1}) + QT_0\left(h_{\tau_0}(i-1)\right), 0\right)$,

$Qx_2(t_i) = max\left(Qx_{2,f}(t_{i-1}) + Qx_{2,h}(t_{i-1}) + b_2(t_i - t_{i-1}) + QT_0\left(h_{\tau_0}(i-1)\right) + QT_2\left(h_{\tau_2}(i-1)\right), 0\right)$.

V.2. Densities just after the competition

This sub-case corresponds to sub-figure *b*) in Fig. 2.

Determination of the density of the $T_{0,1}$*-type protocells surviving the competition (*$QT_{0,1}(t_i)$*)*

This density ($QT_{0,1}(t_i)$) is that of the $T_{0,1}$-type protocells which get x (protocells of density $Q\tilde{T}_0(t_i) + Q\tilde{T}_2(t_i)$ compete for x of density $Qx(t_i)$):

If $Q\tilde{T}_{0,1}(t_i) = 0$ or $Qx(t_i) = 0$, then

$QT_{0,1}(t_i) = 0$,

otherwise,

$QT_{0,1}(t_i) = min\left(\frac{Q\tilde{T}_{0,1}(t_i)Qx(t_i)}{Q\tilde{T}_0(t_i)+Q\tilde{T}_2(t_i)}, Q\tilde{T}_{0,1}(t_i)\right)$.

Determination of the density of the $T_{0,2}$*-type protocells surviving the competition (*$QT_{0,2}(t_i)$*)*

This density ($QT_{0,2,x}(t_i)$) is that of the $T_{0,2}$-type protocells which get x (protocells of density $Q\tilde{T}_{0,2}(t_i) + Q\tilde{T}_2(t_i)$ compete for x of density $Qx(t_i) - QT_{0,1}(t_i)$):

If $Q\tilde{T}_{0,2}(t_i) = 0$ or $Qx(t_i) - QT_{0,1}(t_i) = 0$, then

$QT_{0,2,x}(t_i) = 0$,

otherwise,

$QT_{0,2,x}(t_i) = min\left(\frac{Q\tilde{T}_{0,2}(t_i)\left(Qx(t_i)-QT_{0,1}(t_i)\right)}{Q\tilde{T}_{0,2}(t_i)+Q\tilde{T}_2(t_i)}, Q\tilde{T}_{0,2}(t_i)\right)$.

This density ($QT_{0,2,x,x1}(t_i)$) is that of the $T_{0,2}$-type protocells which get x and x_1 (protocells of density $Q\tilde{T}_{0,2}(t_i) + Q\tilde{T}_2(t_i)$ compete for x_1 of density $Qx_1(t_i)$):

If $QT_{0,2,\mathrm{x}}(t_\mathrm{i}) = 0$ or $Qx_1(t_\mathrm{i}) = 0$, then

$QT_{0,2,\mathrm{x},\mathrm{x1}}(t_\mathrm{i}) = 0$,

otherwise,

$$QT_{0,2,\mathrm{x},\mathrm{x1}}(t_\mathrm{i}) = min\left(\frac{QT_{0,2,\mathrm{x}}(t_\mathrm{i})Qx_1(t_\mathrm{i})}{Q\tilde{T}_{0,2}(t_\mathrm{i})+Q\tilde{T}_2(t_\mathrm{i})}, QT_{0,2,\mathrm{x}}(t_\mathrm{i})\right).$$

This density ($QT_{0,2}(t_\mathrm{i})$) is that of the $T_{0,2}$-type protocells which get x, x_1 and x_2 (protocells of density $Q\tilde{T}_{0,2}(t_\mathrm{i}) + Q\tilde{T}_{2,2}(t_\mathrm{i})$ compete for x_2 of density $Qx_2(t_\mathrm{i})$):

If $QT_{0,2,\mathrm{x},\mathrm{x1}}(t_\mathrm{i}) = 0$ or $Qx_2(t_\mathrm{i}) = 0$, then

$QT_{0,2}(t_\mathrm{i}) = 0$,

otherwise,

$$QT_{0,2}(t_\mathrm{i}) = min\left(\frac{QT_{0,2,\mathrm{x},\mathrm{x1}}(t_\mathrm{i})Qx_2(t_\mathrm{i})}{Q\tilde{T}_{0,2}(t_\mathrm{i})+Q\tilde{T}_{2,2}(t_\mathrm{i})}, QT_{0,2,\mathrm{x},\mathrm{x1}}(t_\mathrm{i})\right).$$

Determination of the density of the $T_{2,1}$-type protocells surviving the competition ($QT_{2,1}(t_\mathrm{i})$)

This density ($QT_{2,1,\mathrm{x}}(t_\mathrm{i})$) is that of the $T_{2,1}$-type protocells which get x (protocells of density $Q\tilde{T}_2(t_\mathrm{i})$ compete for x of density $Qx(t_\mathrm{i}) - QT_{0,1}(t_\mathrm{i}) - QT_{0,2,\mathrm{x}}(t_\mathrm{i})$):

If $Q\tilde{T}_{2,1}(t_\mathrm{i}) = 0$ or $Qx(t_\mathrm{i}) - QT_{0,1}(t_\mathrm{i}) - QT_{0,2,\mathrm{x}}(t_\mathrm{i}) = 0$, then

$QT_{2,1,\mathrm{x}}(t_\mathrm{i}) = 0$,

otherwise,

$$QT_{2,1,\mathrm{x}}(t_\mathrm{i}) = min\left(\frac{Q\tilde{T}_{2,1}(t_\mathrm{i})\left(Qx(t_\mathrm{i})-QT_{0,1}(t_\mathrm{i})-QT_{0,2,\mathrm{x}}(t_\mathrm{i})\right)}{Q\tilde{T}_2(t_\mathrm{i})}, Q\tilde{T}_{2,1}(t_\mathrm{i})\right).$$

This density ($QT_{2,1}(t_\mathrm{i})$) is that of the $T_{2,1}$-type protocells which get x and x_1 (protocells of density $Q\tilde{T}_{0,2}(t_\mathrm{i}) - QT_{0,2,\mathrm{x}}(t_\mathrm{i}) + Q\tilde{T}_2(t_\mathrm{i})$ compete for x_1 of density $Qx_1(t_\mathrm{i}) - QT_{0,2,\mathrm{x},\mathrm{x1}}(t_\mathrm{i})$):

If $QT_{2,1,\mathrm{x}}(t_\mathrm{i}) = 0$ or $Qx_1(t_\mathrm{i}) - QT_{0,2,\mathrm{x},\mathrm{x1}}(t_\mathrm{i}) = 0$, then

$QT_{2,1}(t_\mathrm{i}) = 0$,

otherwise,

$$QT_{2,1}(t_\mathrm{i}) = min\left(\frac{QT_{2,1,\mathrm{x}}(t_\mathrm{i})\left(Qx_1(t_\mathrm{i})-QT_{0,2,\mathrm{x},\mathrm{x1}}(t_\mathrm{i})\right)}{Q\tilde{T}_{0,2}(t_\mathrm{i})-QT_{0,2,\mathrm{x}}(t_\mathrm{i})+Q\tilde{T}_2(t_\mathrm{i})}, QT_{2,1,\mathrm{x}}(t_\mathrm{i})\right).$$

Determination of the density of the $T_{2,2}$-type protocells surviving the competition ($QT_{2,2}(t_\mathrm{i})$)

This density ($QT_{2,2,\mathrm{x}}(t_\mathrm{i})$) is that of the $T_{2,2}$-type protocells which get nutrient x (there is still $Qx(t_\mathrm{i}) - QT_{0,1}(t_i) - QT_{0,2,\mathrm{x}}(t_i) - QT_{2,1,\mathrm{x}}(t_i)$ of x, which is completely left for them):

$$QT_{2,2,\mathrm{x}}(t_\mathrm{i}) = min\left(Qx(t_\mathrm{i}) - QT_{0,1}(t_i) - QT_{0,2,\mathrm{x}}(t_i) - QT_{2,1,\mathrm{x}}(t_i), Q\tilde{T}_{2,2}(t_\mathrm{i})\right).$$

This density ($QT_{2,2,\mathrm{x},\mathrm{x1}}(t_\mathrm{i})$) is that of the $T_{2,2}$-type protocells which get x and x_1 (protocells of density $Q\tilde{T}_{0,2}(t_\mathrm{i}) - QT_{0,2,\mathrm{x}}(t_\mathrm{i}) + Q\tilde{T}_2(t_\mathrm{i}) - QT_{2,1,\mathrm{x}}(t_\mathrm{i})$ compete for x_1 of density $Qx_1(t_\mathrm{i}) - QT_{0,2,\mathrm{x},\mathrm{x1}}(t_\mathrm{i}) - QT_{2,1}(t_\mathrm{i})$):

If $QT_{2,2,\mathrm{x}}(t_\mathrm{i}) = 0$ or $Qx_1(t_\mathrm{i}) - QT_{0,2,\mathrm{x},\mathrm{x1}}(t_\mathrm{i}) - QT_{2,1}(t_\mathrm{i}) = 0$, then

$QT_{2,2,\mathrm{x},\mathrm{x1}}(t_\mathrm{i}) = 0$,

otherwise,

$$QT_{2,2,\mathrm{x,x1}}(t_\mathrm{i}) = min\left(\frac{QT_{2,2,\mathrm{x}}(t_\mathrm{i})\big(Qx_1(t_\mathrm{i}) - QT_{0,2,\mathrm{x,x1}}(t_\mathrm{i}) - QT_{2,1}(t_\mathrm{i})\big)}{Q\tilde{T}_{0,2}(t_\mathrm{i}) - QT_{0,2,\mathrm{x}}(t_\mathrm{i}) + Q\tilde{T}_2(t_\mathrm{i}) - QT_{2,1,\mathrm{x}}(t_\mathrm{i})}, QT_{2,2,\mathrm{x}}(t_\mathrm{i})\right).$$

This density ($QT_{2,2}(t_\mathrm{i})$) is that of the $T_{2,2}$-type protocells which get x, x_1 and x_2 (protocells of density $Q\tilde{T}_{0,2}(t_\mathrm{i}) - QT_{0,2,\mathrm{x,x1}}(t_\mathrm{i}) + Q\tilde{T}_{2,2}(t_\mathrm{i})$ compete for x_2 of density $Qx_2(t_\mathrm{i}) - QT_{0,2}(t_\mathrm{i})$):

If $QT_{2,2,\mathrm{x,x1}}(t_\mathrm{i}) = 0$ or $Qx_2(t_\mathrm{i}) - QT_{0,2}(t_\mathrm{i}) = 0$, then

$QT_{2,2}(t_\mathrm{i}) = 0$,

otherwise,

$$QT_{2,2}(t_\mathrm{i}) = min\left(\frac{QT_{2,2,\mathrm{x,x1}}(t_\mathrm{i})\big(Qx_2(t_\mathrm{i}) - QT_{0,2}(t_\mathrm{i})\big)}{Q\tilde{T}_{0,2}(t_\mathrm{i}) - QT_{0,2,\mathrm{x,x1}}(t_\mathrm{i}) + Q\tilde{T}_{2,2}(t_\mathrm{i})}, QT_{2,2,\mathrm{x,x1}}(t_\mathrm{i})\right).$$

This density of x is not absorbed by the protocells in the competition (surplus of x):

$$Qx_\mathrm{f}(t_\mathrm{i}) = max\big(Qx(t_\mathrm{i}) - Q\tilde{T}_0(t_\mathrm{i}) - Q\tilde{T}_2(t_\mathrm{i}), 0\big).$$

This is the density of x absorbed by the not surviving protocells and, consequently, get out to the environment by the next modeling time point:

$$Qx_\mathrm{h}(t_\mathrm{i}) = min\left(Qx(t_\mathrm{i}) - QT_{0,1}(t_\mathrm{i}), Q\tilde{T}_{0,2}(t_\mathrm{i}) + Q\tilde{T}_2(t_\mathrm{i})\right) - QT_{0,2}(t_\mathrm{i}) - QT_2(t_\mathrm{i}).$$

This density of x_1 is not absorbed by the protocells in the competition (surplus of x_1):

$$Qx_{1,\mathrm{f}}(t_\mathrm{i}) = max\big(Qx_1(t_\mathrm{i}) - Q\tilde{T}_{0,2}(t_\mathrm{i}) - Q\tilde{T}_2(t_\mathrm{i}), 0\big).$$

This is the density of x_1 absorbed by the not surviving protocells and, consequently, get out to the environment by the next modeling time point:

$$Qx_{1,\mathrm{h}}(t_\mathrm{i}) = min\left(Qx_1(t_\mathrm{i}), Q\tilde{T}_{0,2}(t_\mathrm{i}) + Q\tilde{T}_2(t_\mathrm{i})\right) - QT_{0,2}(t_\mathrm{i}) - QT_2(t_\mathrm{i}).$$

This density of x_2 is not absorbed by the protocells in the competition (surplus of x_2):

$$Qx_{2,\mathrm{f}}(t_\mathrm{i}) = max\big(Qx_2(t_\mathrm{i}) - Q\tilde{T}_{0,2}(t_\mathrm{i}) - Q\tilde{T}_{2,2}(t_\mathrm{i}), 0\big).$$

This is the density of x_2 absorbed by the not surviving protocells and, consequently, get out to the environment by the next modeling time point:

$$Qx_{2,\mathrm{h}}(t_\mathrm{i}) = min\left(Qx_2(t_\mathrm{i}), Q\tilde{T}_{0,2}(t_\mathrm{i}) + Q\tilde{T}_{2,2}(t_\mathrm{i})\right) - QT_{0,2}(t_\mathrm{i}) - QT_{2,2}(t_\mathrm{i}).$$

The densities of those protocell types are not changed for which the current modeling time point (t_i) is not an operation time point:

$QT_{1,1}(t_\mathrm{i}) = Q\tilde{T}_{1,1}(t_\mathrm{i})$,

$QT_{1,2}(t_\mathrm{i}) = Q\tilde{T}_{1,2}(t_\mathrm{i})$.

<u>VI.</u> $\boldsymbol{t_\mathrm{i} \neq h_{\tau_0}(i)}$ ($\boldsymbol{t_\mathrm{i} > h_{\tau_0}(i)}$ or $\boldsymbol{h_{\tau_0}(i)}$ is not defined) and $\boldsymbol{t_\mathrm{i} = h_{\tau_1}(i)}$ and $\boldsymbol{t_\mathrm{i} = h_{\tau_2}(i)}$

In this case, t_i is an operation time point of T_1 and T_2 and not an operation time point of T_0.

VI.1. Densities just before the competition

This sub-case corresponds to sub-figure *a*) in Fig. 2.

***VI.1/A)* $\boldsymbol{t_\mathrm{i} = 0}$**

Different densities are not to be determined in this case. Go to the determination of the densities just after the competition, that is, to sub-case VI.2 below.

***VI.1/B)* $\boldsymbol{t_\mathrm{i} > 0}$**

$Q\tilde{T}_{0,1}(t_\mathrm{i}) = QT_{0,1}(t_{\mathrm{i}-1})$,

$Q\tilde{T}_{0,2}(t_{\mathrm{i}}) = QT_{0,2}(t_{\mathrm{i-1}})$,

$Qx(t_{\mathrm{i}}) = max(Qx_{\mathrm{f}}(t_{\mathrm{i-1}}) + Qx_{\mathrm{h}}(t_{\mathrm{i-1}}) + b(t_{\mathrm{i}} - t_{\mathrm{i-1}}), 0)$.

VI.1/B/a) $\boldsymbol{t_{\mathrm{i}} = t_{1,0}}$ ***and*** $\boldsymbol{t_{\mathrm{i}} = t_{2,0}}$

In this case, t_{i} is the initial operation time point of T_1 and T_2.

$Qx_1(t_{\mathrm{i}}) = max\big(Qx_{1,\mathrm{f}}(t_{\mathrm{i-1}}) + Qx_{1,\mathrm{h}}(t_{\mathrm{i-1}}) + \big(b_1(t_{\mathrm{i}} - t_{\mathrm{i-1}})\big), 0\big)$,

$Qx_2(t_{\mathrm{i}}) = max\big(Qx_{2,\mathrm{f}}(t_{\mathrm{i-1}}) + Qx_{2,\mathrm{h}}(t_{\mathrm{i-1}}) + b_2(t_{\mathrm{i}} - t_{\mathrm{i-1}}), 0\big)$.

VI.1/B/b) $\boldsymbol{t_{\mathrm{i}} = t_{1,0}}$ ***and*** $\boldsymbol{t_{\mathrm{i}} > t_{2,0}}$

In this case, t_{i} is the initial operation time point of T_1 but not the initial operation time point of T_2 (yet).

$Q\tilde{T}_{2,1}(t_{\mathrm{i}}) = QT_2\left(h_{\tau_2}(i-1)\right)$,

$Q\tilde{T}_{2,2}(t_{\mathrm{i}}) = QT_{2,1}\left(h_{\tau_2}(i-1)\right)$,

$Qx_1(t_{\mathrm{i}}) = max\big(Qx_{1,\mathrm{f}}(t_{\mathrm{i-1}}) + Qx_{1,\mathrm{h}}(t_{\mathrm{i-1}}) + b_1(t_{\mathrm{i}} - t_{\mathrm{i-1}}), 0\big)$,

$Qx_2(t_{\mathrm{i}}) = max\left(Qx_{2,\mathrm{f}}(t_{\mathrm{i-1}}) + Qx_{2,\mathrm{h}}(t_{\mathrm{i-1}}) + b_2(t_{\mathrm{i}} - t_{\mathrm{i-1}}) + QT_2\left(h_{\tau_2}(i-1)\right), 0\right)$.

VI.1/B/c) $\boldsymbol{t_{\mathrm{i}} > t_{1,0}}$ ***and*** $\boldsymbol{t_{\mathrm{i}} = t_{2,0}}$

In this case, t_{i} is not the initial operation time point of T_1 (yet) but the initial operation time point of T_2.

$Q\tilde{T}_{1,1}(t_{\mathrm{i}}) = QT_1\left(h_{\tau_1}(i-1)\right)$,

$Q\tilde{T}_{1,2}(t_{\mathrm{i}}) = QT_{1,1}\left(h_{\tau_1}(i-1)\right)$,

$Qx_1(t_{\mathrm{i}}) = max\left(Qx_{1,\mathrm{f}}(t_{\mathrm{i-1}}) + Qx_{1,\mathrm{h}}(t_{\mathrm{i-1}}) + b_1(t_{\mathrm{i}} - t_{\mathrm{i-1}}) + QT_1\left(h_{\tau_1}(i-1)\right), 0\right)$,

$Qx_2(t_{\mathrm{i}}) = max\big(Qx_{2,\mathrm{f}}(t_{\mathrm{i-1}}) + Qx_{2,\mathrm{h}}(t_{\mathrm{i-1}}) + b_2(t_{\mathrm{i}} - t_{\mathrm{i-1}}), 0\big)$.

VI.1/B/d) $\boldsymbol{t_{\mathrm{i}} > t_{1,0}}$ ***and*** $\boldsymbol{t_{\mathrm{i}} > t_{2,0}}$

In this case, t_{i} is not the initial operation time point of T_1 and T_2 (yet).

$Q\tilde{T}_{1,1}(t_{\mathrm{i}}) = QT_1\left(h_{\tau_1}(i-1)\right)$,

$Q\tilde{T}_{1,2}(t_{\mathrm{i}}) = QT_{1,1}\left(h_{\tau_1}(i-1)\right)$,

$Q\tilde{T}_{2,1}(t_{\mathrm{i}}) = QT_2\left(h_{\tau_2}(i-1)\right)$,

$Q\tilde{T}_{2,2}(t_{\mathrm{i}}) = QT_{2,1}\left(h_{\tau_2}(i-1)\right)$,

$Qx_1(t_{\mathrm{i}}) = max\left(Qx_{1,\mathrm{f}}(t_{\mathrm{i-1}}) + Qx_{1,\mathrm{h}}(t_{\mathrm{i-1}}) + b_1(t_{\mathrm{i}} - t_{\mathrm{i-1}}) + QT_1\left(h_{\tau_1}(i-1)\right), 0\right)$,

$Qx_2(t_{\mathrm{i}}) = max\left(Qx_{2,\mathrm{f}}(t_{\mathrm{i-1}}) + Qx_{2,\mathrm{h}}(t_{\mathrm{i-1}}) + b_2(t_{\mathrm{i}} - t_{\mathrm{i-1}}) + QT_2\left(h_{\tau_2}(i-1)\right), 0\right)$.

VI.2. Densities just after the competition

This sub-case corresponds to sub-figure b) in Fig. 2.

Determination of the density of the $T_{1,1}$-type protocells surviving the competition ($QT_{1,1}(t_{\mathrm{i}})$)

This density ($QT_{1,1,\mathrm{x}}(t_{\mathrm{i}})$) is that of the $T_{1,1}$-type protocells which get x (protocells of density $Q\tilde{T}_1(t_{\mathrm{i}}) + Q\tilde{T}_2(t_{\mathrm{i}})$ compete for x of density $Qx(t_{\mathrm{i}})$):

If $Q\tilde{T}_{1,1}(t_i) = 0$ or $Qx(t_i) = 0$, then

$QT_{1,1,x}(t_i) = 0$,

otherwise,

$$QT_{1,1,x}(t_i) = min\left(\frac{Q\tilde{T}_{1,1}(t_i)Qx(t_i)}{Q\tilde{T}_1(t_i)+Q\tilde{T}_2(t_i)}, Q\tilde{T}_{1,1}(t_i)\right).$$

This density ($QT_{1,1}(t_i)$) is that of the $T_{1,1}$-type protocells which get x and x_2 (protocells of density $Q\tilde{T}_1(t_i) + Q\tilde{T}_{2,2}(t_i)$ compete for x_2 of density $Qx_2(t_i)$):

If $QT_{1,1,x}(t_i) = 0$ or $Qx_2(t_i) = 0$, then

$QT_{1,1}(t_i) = 0$,

otherwise,

$$QT_{1,1}(t_i) = min\left(\frac{QT_{1,1,x}(t_i)Qx_2(t_i)}{Q\tilde{T}_1(t_i)+Q\tilde{T}_{2,2}(t_i)}, QT_{1,1,x}(t_i)\right).$$

Determination of the density of the $T_{1,2}$-type protocells surviving the competition ($QT_{1,2}(t_i)$)

This density ($QT_{1,2,x}(t_i)$) is that of the $T_{1,2}$-type protocells which get x (protocells of density $Q\tilde{T}_{1,2}(t_i) + Q\tilde{T}_2(t_i)$ compete for x of density $Qx(t_i) - QT_{1,1,x}(t_i)$):

If $Q\tilde{T}_{1,2}(t_i) = 0$ or $Qx(t_i) - QT_{1,1,x}(t_i) = 0$, then

$QT_{1,2,x}(t_i) = 0$,

otherwise,

$$QT_{1,2,x}(t_i) = min\left(\frac{Q\tilde{T}_{1,2}(t_i)\left(Qx(t_i)-QT_{1,1,x}(t_i)\right)}{Q\tilde{T}_{1,2}(t_i)+Q\tilde{T}_2(t_i)}, Q\tilde{T}_{1,2}(t_i)\right).$$

This density ($QT_{1,2,x,x1}(t_i)$) is that of the $T_{1,2}$-type protocells which get x and x_1 (protocells of density $Q\tilde{T}_{1,2}(t_i) + Q\tilde{T}_2(t_i)$ compete for x_1 of density $Qx_1(t_i)$):

If $QT_{1,2,x}(t_i) = 0$ or $Qx_1(t_i) = 0$, then

$QT_{1,2,x,x1}(t_i) = 0$,

otherwise,

$$QT_{1,2,x,x1}(t_i) = min\left(\frac{QT_{1,2,x}(t_i)Qx_1(t_i)}{Q\tilde{T}_{1,2}(t_i)+Q\tilde{T}_2(t_i)}, QT_{1,2,x}(t_i)\right).$$

This density ($QT_{1,2}(t_i)$) is that of the $T_{1,2}$-type protocells which get x, x_1 and x_2 (protocells of density $Q\tilde{T}_1(t_i) - QT_{1,1,x}(t_i) + Q\tilde{T}_{2,2}(t_i)$ compete for x_2 of density $Qx_2(t_i) - QT_{1,1}(t_i)$):

If $QT_{1,2,x,x1}(t_i) = 0$ or $Qx_2(t_i) - QT_{1,1}(t_i) = 0$, then

$QT_{1,2}(t_i) = 0$,

otherwise,

$$QT_{1,2}(t_i) = min\left(\frac{QT_{1,2,x,x1}(t_i)\left(Qx_2(t_i)-QT_{1,1}(t_i)\right)}{Q\tilde{T}_1(t_i)-QT_{1,1,x}(t_i)+Q\tilde{T}_{2,2}(t_i)}, QT_{1,2,x,x1}(t_i)\right).$$

Determination of the density of the $T_{2,1}$-type protocells surviving the competition ($QT_{2,1}(t_i)$)

This density ($QT_{2,1,x}(t_i)$) is that of the $T_{2,1}$-type protocells which get x (protocells of density $Q\tilde{T}_2(t_i)$ compete for x of density $Qx(t_i) - QT_{1,1,x}(t_i) - QT_{1,2,x}(t_i)$):

If $Q\tilde{T}_{2,1}(t_{\mathrm{i}}) = 0$ or $Qx(t_{\mathrm{i}}) - QT_{1,1,\mathrm{x}}(t_{\mathrm{i}}) - QT_{1,2,\mathrm{x}}(t_{\mathrm{i}}) = 0$, then

$QT_{2,1,\mathrm{x}}(t_{\mathrm{i}}) = 0$,

otherwise,

$$QT_{2,1,\mathrm{x}}(t_{\mathrm{i}}) = min\left(\frac{Q\tilde{T}_{2,1}(t_{\mathrm{i}})\big(Qx(t_{\mathrm{i}}) - QT_{1,1,\mathrm{x}}(t_{\mathrm{i}}) - QT_{1,2,\mathrm{x}}(t_{\mathrm{i}})\big)}{Q\tilde{T}_{2}(t_{\mathrm{i}})}, Q\tilde{T}_{2,1}(t_{\mathrm{i}})\right).$$

This density ($QT_{2,1}(t_{\mathrm{i}})$) is that of the $T_{2,1}$-type protocells which get x and x_1 (protocells of density $Q\tilde{T}_{1,2}(t_{\mathrm{i}}) - QT_{1,2,\mathrm{x}}(t_{\mathrm{i}}) + Q\tilde{T}_{2}(t_{\mathrm{i}})$ compete for x_1 of density $Qx_1(t_{\mathrm{i}}) - QT_{1,2,\mathrm{x,x1}}(t_{\mathrm{i}})$):

If $QT_{2,1,\mathrm{x}}(t_{\mathrm{i}}) = 0$ or $Qx_1(t_{\mathrm{i}}) - QT_{1,2,\mathrm{x,x1}}(t_{\mathrm{i}}) = 0$, then

$QT_{2,1}(t_{\mathrm{i}}) = 0$,

otherwise,

$$QT_{2,1}(t_{\mathrm{i}}) = min\left(\frac{QT_{2,1,\mathrm{x}}(t_{\mathrm{i}})\big(Qx_1(t_{\mathrm{i}}) - QT_{1,2,\mathrm{x,x1}}(t_{\mathrm{i}})\big)}{Q\tilde{T}_{1,2}(t_{\mathrm{i}}) - QT_{1,2,\mathrm{x}}(t_{\mathrm{i}}) + Q\tilde{T}_{2}(t_{\mathrm{i}})}, QT_{2,1,\mathrm{x}}(t_{\mathrm{i}})\right).$$

Determination of the density of the $T_{2,2}$-type protocells surviving the competition ($QT_{2,2}(t_{\mathrm{i}})$)

This density ($QT_{2,2,\mathrm{x}}(t_{\mathrm{i}})$) is that of the $T_{2,2}$-type protocells which get nutrient x (there is still $Qx(t_{\mathrm{i}}) - QT_{1,1,\mathrm{x}}(t_i) - QT_{1,2,\mathrm{x}}(t_i) - QT_{2,1,\mathrm{x}}(t_i)$ of x, which is completely left for them):

$$QT_{2,2,\mathrm{x}}(t_{\mathrm{i}}) = min\left(Qx(t_{\mathrm{i}}) - QT_{1,1,\mathrm{x}}(t_i) - QT_{1,2,\mathrm{x}}(t_i) - QT_{2,1,\mathrm{x}}(t_i), Q\tilde{T}_{2,2}(t_{\mathrm{i}})\right).$$

This density ($QT_{2,2,\mathrm{x,x1}}(t_{\mathrm{i}})$) is that of the $T_{2,2}$-type protocells which get x and x_1 (protocells of density $Q\tilde{T}_{1,2}(t_{\mathrm{i}}) - QT_{1,2,\mathrm{x}}(t_{\mathrm{i}}) + Q\tilde{T}_{2}(t_{\mathrm{i}}) - QT_{2,1,\mathrm{x}}(t_{\mathrm{i}})$ compete for x_1 of density $Qx_1(t_{\mathrm{i}}) - QT_{1,2,\mathrm{x,x1}}(t_{\mathrm{i}}) - QT_{2,1}(t_{\mathrm{i}})$):

If $QT_{2,2,\mathrm{x}}(t_{\mathrm{i}}) = 0$ or $Qx_1(t_{\mathrm{i}}) - QT_{1,2,\mathrm{x,x1}}(t_{\mathrm{i}}) - QT_{2,1}(t_{\mathrm{i}}) = 0$, then

$QT_{2,2,\mathrm{x,x1}}(t_{\mathrm{i}}) = 0$,

otherwise,

$$QT_{2,2,\mathrm{x,x1}}(t_{\mathrm{i}}) = min\left(\frac{QT_{2,2,\mathrm{x}}(t_{\mathrm{i}})\big(Qx_1(t_{\mathrm{i}}) - QT_{1,2,\mathrm{x,x1}}(t_{\mathrm{i}}) - QT_{2,1}(t_{\mathrm{i}})\big)}{Q\tilde{T}_{1,2}(t_{\mathrm{i}}) - QT_{1,2,\mathrm{x}}(t_{\mathrm{i}}) + Q\tilde{T}_{2}(t_{\mathrm{i}}) - QT_{2,1,\mathrm{x}}(t_{\mathrm{i}})}, QT_{2,2,\mathrm{x}}(t_{\mathrm{i}})\right).$$

This density ($QT_{2,2}(t_{\mathrm{i}})$) is that of the $T_{2,2}$-type protocells which get x, x_1 and x_2 (protocells of density $Q\tilde{T}_{1}(t_{\mathrm{i}}) - QT_{1,1,\mathrm{x}}(t_{\mathrm{i}}) - QT_{1,2,\mathrm{x,x1}}(t_{\mathrm{i}}) + Q\tilde{T}_{2,2}(t_{\mathrm{i}})$ compete for x_2 of density $Qx_2(t_{\mathrm{i}}) - QT_{1,1}(t_{\mathrm{i}}) - QT_{1,2}(t_{\mathrm{i}})$):

If $QT_{2,2,\mathrm{x,x1}}(t_{\mathrm{i}}) = 0$ or $Qx_2(t_{\mathrm{i}}) - QT_{1}(t_{\mathrm{i}}) = 0$, then

$QT_{2,2}(t_{\mathrm{i}}) = 0$,

otherwise,

$$QT_{2,2}(t_{\mathrm{i}}) = min\left(\frac{QT_{2,2,\mathrm{x,x1}}(t_{\mathrm{i}})\big(Qx_2(t_{\mathrm{i}}) - QT_{1}(t_{\mathrm{i}})\big)}{Q\tilde{T}_{1}(t_{\mathrm{i}}) - QT_{1,1,\mathrm{x}}(t_{\mathrm{i}}) - QT_{1,2,\mathrm{x,x1}}(t_{\mathrm{i}}) + Q\tilde{T}_{2,2}(t_{\mathrm{i}})}, QT_{2,2,\mathrm{x,x1}}(t_{\mathrm{i}})\right).$$

This density of x is not absorbed by the protocells in the competition (surplus of x):

$$Qx_{\mathrm{f}}(t_{\mathrm{i}}) = max\big(Qx(t_{\mathrm{i}}) - Q\tilde{T}_{1}(t_{\mathrm{i}}) - Q\tilde{T}_{2}(t_{\mathrm{i}}), 0\big).$$

This is the density of x absorbed by the not surviving protocells and, consequently, get out to the environment by the next modeling time point:

$Qx_{\mathrm{h}}(t_{\mathrm{i}}) = min\left(Qx(t_{\mathrm{i}}), Q\tilde{T}_1(t_{\mathrm{i}}) + Q\tilde{T}_2(t_{\mathrm{i}})\right) - QT_1(t_{\mathrm{i}}) - QT_2(t_{\mathrm{i}})$.

This density of x_1 is not absorbed by the protocells in the competition (surplus of x_1):

$Qx_{1,\mathrm{f}}(t_{\mathrm{i}}) = max\left(Qx_1(t_{\mathrm{i}}) - Q\tilde{T}_{1,2}(t_{\mathrm{i}}) - Q\tilde{T}_2(t_{\mathrm{i}}), 0\right)$.

This is the density of x_1 absorbed by the not surviving protocells and, consequently, get out to the environment by the next modeling time point:

$Qx_{1,\mathrm{h}}(t_{\mathrm{i}}) = min\left(Qx_1(t_{\mathrm{i}}), Q\tilde{T}_{1,2}(t_{\mathrm{i}}) + Q\tilde{T}_2(t_{\mathrm{i}})\right) - QT_{1,2}(t_{\mathrm{i}}) - QT_2(t_{\mathrm{i}})$.

This density of x_2 is not absorbed by the protocells in the competition (surplus of x_2):

$Qx_{2,\mathrm{f}}(t_{\mathrm{i}}) = max\left(Qx_2(t_{\mathrm{i}}) - Q\tilde{T}_1(t_{\mathrm{i}}) - Q\tilde{T}_{2,2}(t_{\mathrm{i}}), 0\right)$.

This is the density of x_2 absorbed by the not surviving protocells and, consequently, get out to the environment by the next modeling time point:

$Qx_{2,\mathrm{h}}(t_{\mathrm{i}}) = min\left(Qx_2(t_{\mathrm{i}}), Q\tilde{T}_1(t_{\mathrm{i}}) + Q\tilde{T}_{2,2}(t_{\mathrm{i}})\right) - QT_1(t_{\mathrm{i}}) - QT_{2,2}(t_{\mathrm{i}})$.

The densities of those protocell types are not changed for which the current modeling time point (t_{i}) is not an operation time point:

$QT_{0,1}(t_{\mathrm{i}}) = Q\tilde{T}_{0,1}(t_{\mathrm{i}})$,

$QT_{0,2}(t_{\mathrm{i}}) = Q\tilde{T}_{0,2}(t_{\mathrm{i}})$.

VII. $\boldsymbol{t_{\mathrm{i}} = h_{\tau_0}(i)}$ **and** $\boldsymbol{t_{\mathrm{i}} = h_{\tau_1}(i)}$ **and** $\boldsymbol{t_{\mathrm{i}} = h_{\tau_2}(i)}$
In this case, t_{i} is an operation time point of T_0, T_1 and T_2.

VII.1. Densities just before the competition

This sub-case corresponds to sub-figure *a*) in Fig. 2.

VII.1/A) $\boldsymbol{t_{\mathrm{i}} = 0}$
Different densities are not to be determined in this case. Go to the determination of the densities just after the competition, that is, to sub-case VII.2 below.

VII.1/B) $\boldsymbol{t_{\mathrm{i}} > 0}$

$Qx(t_{\mathrm{i}}) = max(Qx_{\mathrm{f}}(t_{\mathrm{i-1}}) + Qx_{\mathrm{h}}(t_{\mathrm{i-1}}) + b(t_{\mathrm{i}} - t_{\mathrm{i-1}}), 0)$.

VII.1/B/a) $\boldsymbol{t_{\mathrm{i}} = t_{0,0}}$ ***and*** $\boldsymbol{t_{\mathrm{i}} = t_{1,0}}$ ***and*** $\boldsymbol{t_{\mathrm{i}} = t_{2,0}}$
In this case, t_{i} is the initial operation time point of T_0, T_1 and T_2.

$Qx_1(t_{\mathrm{i}}) = max\left(Qx_{1,\mathrm{f}}(t_{\mathrm{i-1}}) + Qx_{1,\mathrm{h}}(t_{\mathrm{i-1}}) + b_1(t_{\mathrm{i}} - t_{\mathrm{i-1}}), 0\right)$,

$Qx_2(t_{\mathrm{i}}) = max\left(Qx_{2,\mathrm{f}}(t_{\mathrm{i-1}}) + Qx_{2,\mathrm{h}}(t_{\mathrm{i-1}}) + b_2(t_{\mathrm{i}} - t_{\mathrm{i-1}}), 0\right)$.

VII.1/B/b) $\boldsymbol{t_{\mathrm{i}} = t_{0,0}}$ ***and*** $\boldsymbol{t_{\mathrm{i}} = t_{1,0}}$ ***and*** $\boldsymbol{t_{\mathrm{i}} > t_{2,0}}$
In this case, t_{i} is the initial operation time point of T_0 and T_1 but not the initial operation time point of T_2 (yet).

$Q\tilde{T}_{2,1}(t_{\mathrm{i}}) = QT_2\left(h_{\tau_2}(i-1)\right)$,

$Q\tilde{T}_{2,2}(t_{\mathrm{i}}) = QT_{2,1}\left(h_{\tau_2}(i-1)\right)$,

$Qx_1(t_{\mathrm{i}}) = max\left(Qx_{1,\mathrm{f}}(t_{\mathrm{i-1}}) + Qx_{1,\mathrm{h}}(t_{\mathrm{i-1}}) + b_1(t_{\mathrm{i}} - t_{\mathrm{i-1}}), 0\right)$,

$Qx_2(t_{\mathrm{i}}) = max\left(Qx_{2,\mathrm{f}}(t_{\mathrm{i-1}}) + Qx_{2,\mathrm{h}}(t_{\mathrm{i-1}}) + b_2(t_{\mathrm{i}} - t_{\mathrm{i-1}}) + QT_2\left(h_{\tau_2}(i-1)\right), 0\right)$.

VII.1/B/c) $\boldsymbol{t_{\mathrm{i}} = t_{0,0}}$ ***and*** $\boldsymbol{t_{\mathrm{i}} > t_{1,0}}$ ***and*** $\boldsymbol{t_i = t_{2,0}}$

In this case, t_{i} is the initial operation time point of T_0 and T_2 but not the initial operation time point of T_1 (yet).

$$Q\tilde{T}_{1,1}(t_{\mathrm{i}}) = QT_1\left(h_{\tau_1}(i-1)\right),$$

$$Q\tilde{T}_{1,2}(t_{\mathrm{i}}) = QT_{1,1}\left(h_{\tau_1}(i-1)\right),$$

$$Qx_1(t_{\mathrm{i}}) = max\left(Qx_{1,\mathrm{f}}(t_{\mathrm{i-1}}) + Qx_{1,\mathrm{h}}(t_{\mathrm{i-1}}) + b_1(t_{\mathrm{i}} - t_{\mathrm{i-1}}) + QT_1\left(h_{\tau_1}(i-1)\right), 0\right),$$

$$Qx_2(t_{\mathrm{i}}) = max\left(Qx_{2,\mathrm{f}}(t_{\mathrm{i-1}}) + Qx_{2,\mathrm{h}}(t_{\mathrm{i-1}}) + b_2(t_{\mathrm{i}} - t_{\mathrm{i-1}}), 0\right).$$

VII.1/B/d) $\boldsymbol{t_{\mathrm{i}} = t_{0,0}}$ ***and*** $\boldsymbol{t_{\mathrm{i}} > t_{1,0}}$ ***and*** $\boldsymbol{t_{\mathrm{i}} > t_{2,0}}$

In this case, t_{i} is the initial operation time point of T_0 but not the initial operation time point of T_1 and T_2 (yet).

$$Q\tilde{T}_{1,1}(t_{\mathrm{i}}) = QT_1\left(h_{\tau_1}(i-1)\right),$$

$$Q\tilde{T}_{1,2}(t_{\mathrm{i}}) = QT_{1,1}\left(h_{\tau_1}(i-1)\right),$$

$$Q\tilde{T}_{2,1}(t_{\mathrm{i}}) = QT_2\left(h_{\tau_2}(i-1)\right),$$

$$Q\tilde{T}_{2,2}(t_{\mathrm{i}}) = QT_{2,1}\left(h_{\tau_2}(i-1)\right),$$

$$Qx_1(t_{\mathrm{i}}) = max\left(Qx_{1,\mathrm{f}}(t_{\mathrm{i-1}}) + Qx_{1,\mathrm{h}}(t_{\mathrm{i-1}}) + b_1(t_{\mathrm{i}} - t_{\mathrm{i-1}}) + QT_1\left(h_{\tau_1}(i-1)\right), 0\right),$$

$$Qx_2(t_{\mathrm{i}}) = max\left(Qx_{2,\mathrm{f}}(t_{\mathrm{i-1}}) + Qx_{2,\mathrm{h}}(t_{\mathrm{i-1}}) + b_2(t_{\mathrm{i}} - t_{\mathrm{i-1}}) + QT_2\left(h_{\tau_2}(i-1)\right), 0\right).$$

VII.1/B/e) $\boldsymbol{t_{\mathrm{i}} > t_{0,0}}$ ***and*** $\boldsymbol{t_{\mathrm{i}} = t_{1,0}}$ ***and*** $\boldsymbol{t_{\mathrm{i}} = t_{2,0}}$

In this case, t_{i} is not the initial operation time point of T_0 (yet) but the initial operation time point of T_1 and T_2.

$$Q\tilde{T}_{0,1}(t_{\mathrm{i}}) = QT_0\left(h_{\tau_0}(i-1)\right),$$

$$Q\tilde{T}_{0,2}(t_{\mathrm{i}}) = QT_{0,1}\left(h_{\tau_0}(i-1)\right),$$

$$Qx_1(t_{\mathrm{i}}) = max\left(Qx_{1,\mathrm{f}}(t_{\mathrm{i-1}}) + Qx_{1,\mathrm{h}}(t_{\mathrm{i-1}}) + b_1(t_{\mathrm{i}} - t_{\mathrm{i-1}}) + QT_0\left(h_{\tau_0}(i-1)\right), 0\right),$$

$$Qx_2(t_{\mathrm{i}}) = max\left(Qx_{2,\mathrm{f}}(t_{\mathrm{i-1}}) + Qx_{2,\mathrm{h}}(t_{\mathrm{i-1}}) + b_2(t_{\mathrm{i}} - t_{\mathrm{i-1}}) + QT_0\left(h_{\tau_0}(i-1)\right), 0\right).$$

VII.1/B/f) $\boldsymbol{t_{\mathrm{i}} > t_{0,0}}$ ***and*** $\boldsymbol{t_{\mathrm{i}} = t_{1,0}}$ ***and*** $\boldsymbol{t_{\mathrm{i}} > t_{2,0}}$

In this case, t_{i} is not the initial operation time point of T_0 and T_2 (yet) but the initial operation time point of T_1.

$$Q\tilde{T}_{0,1}(t_{\mathrm{i}}) = QT_0\left(h_{\tau_0}(i-1)\right),$$

$$Q\tilde{T}_{0,2}(t_{\mathrm{i}}) = QT_{0,1}\left(h_{\tau_0}(i-1)\right),$$

$$Q\tilde{T}_{2,1}(t_{\mathrm{i}}) = QT_2\left(h_{\tau_2}(i-1)\right),$$

$$Q\tilde{T}_{2,2}(t_{\mathrm{i}}) = QT_{2,1}\left(h_{\tau_2}(i-1)\right),$$

$$Qx_1(t_{\mathrm{i}}) = max\left(Qx_{1,\mathrm{f}}(t_{\mathrm{i-1}}) + Qx_{1,\mathrm{h}}(t_{\mathrm{i-1}}) + b_1(t_{\mathrm{i}} - t_{\mathrm{i-1}}) + QT_0\left(h_{\tau_0}(i-1)\right), 0\right),$$

$Qx_2(t_i) = max\left(Qx_{2,f}(t_{i-1}) + Qx_{2,h}(t_{i-1}) + b_2(t_i - t_{i-1}) + QT_0\left(h_{\tau_0}(i-1)\right) + QT_2\left(h_{\tau_2}(i-1)\right), 0\right).$

VII.1/B/g) $\boldsymbol{t_i > t_{0,0}}$ ***and*** $\boldsymbol{t_i > t_{1,0}}$ ***and*** $\boldsymbol{t_i = t_{2,0}}$

In this case, t_i is not the initial operation time point of T_0 and T_1 (yet) but the initial operation time point of T_2.

$Q\tilde{T}_{0,1}(t_i) = QT_0\left(h_{\tau_0}(i-1)\right),$

$Q\tilde{T}_{0,2}(t_i) = QT_{0,1}\left(h_{\tau_0}(i-1)\right),$

$Q\tilde{T}_{1,1}(t_i) = QT_1\left(h_{\tau_1}(i-1)\right),$

$Q\tilde{T}_{1,2}(t_i) = QT_{1,1}\left(h_{\tau_1}(i-1)\right),$

$Qx_1(t_i) = max\left(Qx_{1,f}(t_{i-1}) + Qx_{1,h}(t_{i-1}) + b_1(t_i - t_{i-1}) + QT_0\left(h_{\tau_0}(i-1)\right) + QT_1\left(h_{\tau_1}(i-1)\right), 0\right),$

$Qx_2(t_i) = max\left(Qx_{2,f}(t_{i-1}) + Qx_{2,h}(t_{i-1}) + b_2(t_i - t_{i-1}) + QT_0\left(h_{\tau_0}(i-1)\right), 0\right).$

VII.1/B/h) $\boldsymbol{t_i > t_{0,0}}$ ***and*** $\boldsymbol{t_i > t_{1,0}}$ ***and*** $\boldsymbol{t_i > t_{2,0}}$

In this case, t_i is not the initial operation time point of T_0, T_1 and T_2 (yet).

$Q\tilde{T}_{0,1}(t_i) = QT_0\left(h_{\tau_0}(i-1)\right),$

$Q\tilde{T}_{0,2}(t_i) = QT_{0,1}\left(h_{\tau_0}(i-1)\right),$

$Q\tilde{T}_{1,1}(t_i) = QT_1\left(h_{\tau_1}(i-1)\right),$

$Q\tilde{T}_{1,2}(t_i) = QT_{1,1}\left(h_{\tau_1}(i-1)\right),$

$Q\tilde{T}_{2,1}(t_i) = QT_2\left(h_{\tau_2}(i-1)\right),$

$Q\tilde{T}_{2,2}(t_i) = QT_{2,1}\left(h_{\tau_2}(i-1)\right),$

$Qx_1(t_i) = max\left(Qx_{1,f}(t_{i-1}) + Qx_{1,h}(t_{i-1}) + b_1(t_i - t_{i-1}) + QT_0\left(h_{\tau_0}(i-1)\right) + QT_1\left(h_{\tau_1}(i-1)\right), 0\right),$

$Qx_2(t_i) = max\left(Qx_{2,f}(t_{i-1}) + Qx_{2,h}(t_{i-1}) + b_2(t_i - t_{i-1}) + QT_0\left(h_{\tau_0}(i-1)\right) + QT_2\left(h_{\tau_2}(i-1)\right), 0\right).$

VII.2. Densities just after the competition

This sub-case corresponds to sub-figure *b*) in Fig. 2.

Determination of the density of the $T_{0,1}$-type protocells surviving the competition ($QT_{0,1}(t_i)$)

This density ($QT_{0,1}(t_i)$) is that of the $T_{0,1}$-type protocells which get x (protocells of density $Q\tilde{T}(t_i)$ compete for x of density $Qx(t_i)$):

If $Q\tilde{T}_{0,1}(t_i) = 0$ or $Qx(t_i) = 0$, then

$QT_{0,1}(t_i) = 0,$

otherwise,

$$QT_{0,1}(t_i) = min\left(\frac{Q\tilde{T}_{0,1}(t_i)Qx(t_i)}{Q\tilde{T}(t_i)}, Q\tilde{T}_{0,1}(t_i)\right).$$

Determination of the density of the $T_{0,2}$-type protocells surviving the competition ($QT_{0,2}(t_i)$)

This density ($QT_{0,2,x}(t_i)$) is that of the $T_{0,2}$-type protocells which get x (protocells of density $Q\tilde{T}_{0,2}(t_i) + Q\tilde{T}_1(t_i) + Q\tilde{T}_2(t_i)$ compete for x of density $Qx(t_i) - QT_{0,1}(t_i)$):

If $Q\tilde{T}_{0,2}(t_i) = 0$ or $Qx(t_i) - QT_{0,1}(t_i) = 0$, then

$QT_{0,2,x}(t_i) = 0$,

otherwise,

$$QT_{0,2,x}(t_i) = min\left(\frac{Q\tilde{T}_{0,2}(t_i)\left(Qx(t_i) - QT_{0,1}(t_i)\right)}{Q\tilde{T}_{0,2}(t_i) + Q\tilde{T}_1(t_i) + Q\tilde{T}_2(t_i)}, Q\tilde{T}_{0,2}(t_i)\right).$$

This density ($QT_{0,2,x,x1}(t_i)$) is that of the $T_{0,2}$-type protocells which get x and x_1 (protocells of density $Q\tilde{T}_{0,2}(t_i) + Q\tilde{T}_{1,2}(t_i) + Q\tilde{T}_2(t_i)$ compete for x_1 of density $Qx_1(t_i)$):

If $QT_{0,2,x}(t_i) = 0$ or $Qx_1(t_i) = 0$, then

$QT_{0,2,x,x1}(t_i) = 0$,

otherwise,

$$QT_{0,2,x,x1}(t_i) = min\left(\frac{QT_{0,2,x}(t_i)Qx_1(t_i)}{Q\tilde{T}_{0,2}(t_i) + Q\tilde{T}_{1,2}(t_i) + Q\tilde{T}_2(t_i)}, QT_{0,2,x}(t_i)\right).$$

This density ($QT_{0,2}(t_i)$) is that of the $T_{0,2}$-type protocells which get x, x_1 and x_2 (protocells of density $Q\tilde{T}_{0,2}(t_i) + Q\tilde{T}_1(t_i) + Q\tilde{T}_{2,2}(t_i)$ compete for x_2 of density $Qx_2(t_i)$):

If $QT_{0,2,x,x1}(t_i) = 0$ or $Qx_2(t_i) = 0$, then

$QT_{0,2}(t_i) = 0$,

otherwise,

$$QT_{0,2}(t_i) = min\left(\frac{QT_{0,2,x,x1}(t_i)Qx_2(t_i)}{Q\tilde{T}_{0,2}(t_i) + Q\tilde{T}_1(t_i) + Q\tilde{T}_{2,2}(t_i)}, QT_{0,2,x,x1}(t_i)\right).$$

Determination of the density of the $T_{1,1}$-type protocells surviving the competition ($QT_{1,1}(t_i)$)

This density ($QT_{1,1,x}(t_i)$) is that of the $T_{1,1}$-type protocells which get x (protocells of density $Q\tilde{T}_1(t_i) + Q\tilde{T}_2(t_i)$ compete for x of density $Qx(t_i) - QT_{0,1}(t_i) - QT_{0,2,x}(t_i)$):

If $Q\tilde{T}_{1,1}(t_i) = 0$ or $Qx(t_i) - QT_{0,1}(t_i) - QT_{0,2,x}(t_i) = 0$, then

$QT_{1,1,x}(t_i) = 0$,

otherwise,

$$QT_{1,1,x}(t_i) = min\left(\frac{Q\tilde{T}_{1,1}(t_i)\left(Qx(t_i) - QT_{0,1}(t_i) - QT_{0,2,x}(t_i)\right)}{Q\tilde{T}_1(t_i) + Q\tilde{T}_2(t_i)}, Q\tilde{T}_{1,1}(t_i)\right).$$

This density ($QT_{1,1}(t_i)$) is that of the $T_{1,1}$-type protocells which get x and x_2 (protocells of density $Q\tilde{T}_{0,2}(t_i) - QT_{0,2,x,x1}(t_i) + Q\tilde{T}_1(t_i) + Q\tilde{T}_{2,2}(t_i)$ compete for x_2 of density $Qx_2(t_i) - QT_{0,2}(t_i)$):

If $QT_{1,1,x}(t_i) = 0$ or $Qx_2(t_i) - QT_{0,2}(t_i) = 0$, then

$QT_{1,1}(t_\mathrm{i}) = 0,$

otherwise,

$$QT_{1,1}(t_\mathrm{i}) = min\left(\frac{QT_{1,1,\mathrm{x}}(t_\mathrm{i})\big(Qx_2(t_\mathrm{i}) - QT_{0,2}(t_\mathrm{i})\big)}{Q\tilde{T}_{0,2}(t_\mathrm{i}) - QT_{0,2,\mathrm{x,x1}}(t_\mathrm{i}) + Q\tilde{T}_1(t_\mathrm{i}) + Q\tilde{T}_{2,2}(t_\mathrm{i})}, QT_{1,1,\mathrm{x}}(t_\mathrm{i})\right).$$

Determination of the density of the $T_{1,2}$-type protocells surviving the competition ($QT_{1,2}(t_\mathrm{i})$)

This density ($QT_{1,2,\mathrm{x}}(t_\mathrm{i})$) is that of the $T_{1,2}$-type protocells which get x (protocells of density $Q\tilde{T}_{1,2}(t_\mathrm{i}) + Q\tilde{T}_2(t_\mathrm{i})$ compete for x of density $Qx(t_\mathrm{i}) - QT_{0,1}(t_\mathrm{i}) - QT_{0,2,\mathrm{x}}(t_\mathrm{i}) - QT_{1,1,\mathrm{x}}(t_\mathrm{i})$):

If $Q\tilde{T}_{1,2}(t_\mathrm{i}) = 0$ or $Qx(t_\mathrm{i}) - QT_{0,1}(t_\mathrm{i}) - QT_{0,2,\mathrm{x}}(t_\mathrm{i}) - QT_{1,1,\mathrm{x}}(t_\mathrm{i}) = 0$, then

$QT_{1,2,\mathrm{x}}(t_\mathrm{i}) = 0,$

otherwise,

$$QT_{1,2,\mathrm{x}}(t_\mathrm{i}) = min\left(\frac{Q\tilde{T}_{1,2}(t_\mathrm{i})\big(Qx(t_\mathrm{i}) - QT_{0,1}(t_\mathrm{i}) - QT_{0,2,\mathrm{x}}(t_\mathrm{i}) - QT_{1,1,\mathrm{x}}(t_\mathrm{i})\big)}{Q\tilde{T}_{1,2}(t_\mathrm{i}) + Q\tilde{T}_2(t_\mathrm{i})}, QT_{1,2,\mathrm{x}}(t_\mathrm{i})\right).$$

This density ($QT_{1,2,\mathrm{x,x1}}(t_\mathrm{i})$) is that of the $T_{1,2}$-type protocells which get x and x_1 (protocells of density $Q\tilde{T}_{0,2}(t_\mathrm{i}) - QT_{0,2,\mathrm{x}}(t_\mathrm{i}) + Q\tilde{T}_{1,2}(t_\mathrm{i}) + Q\tilde{T}_2(t_\mathrm{i})$ compete for x_1 of density $Qx_1(t_\mathrm{i}) - QT_{0,2,\mathrm{x,x1}}(t_\mathrm{i})$):

If $QT_{1,2,\mathrm{x}}(t_\mathrm{i}) = 0$ or $Qx_1(t_\mathrm{i}) - QT_{0,2,\mathrm{x,x1}}(t_\mathrm{i}) = 0$, then

$QT_{1,2,\mathrm{x,x1}}(t_\mathrm{i}) = 0,$

otherwise,

$$QT_{1,2,\mathrm{x,x1}}(t_\mathrm{i}) = min\left(\frac{QT_{1,2,\mathrm{x}}(t_\mathrm{i})\big(Qx_1(t_\mathrm{i}) - QT_{0,2,\mathrm{x,x1}}(t_\mathrm{i})\big)}{Q\tilde{T}_{0,2}(t_\mathrm{i}) - QT_{0,2,\mathrm{x}}(t_\mathrm{i}) + Q\tilde{T}_{1,2}(t_\mathrm{i}) + Q\tilde{T}_2(t_\mathrm{i})}, QT_{1,2,\mathrm{x}}(t_\mathrm{i})\right).$$

This density ($QT_{1,2}(t_\mathrm{i})$) is that of the $T_{1,2}$-type protocells which get x, x_1 and x_2 (protocells of density $Q\tilde{T}_{0,2}(t_\mathrm{i}) - QT_{0,2,\mathrm{x,x1}}(t_\mathrm{i}) + Q\tilde{T}_1(t_\mathrm{i}) - QT_{1,1,\mathrm{x}}(t_\mathrm{i}) + Q\tilde{T}_{2,2}(t_\mathrm{i})$ compete for x_2 of density $Qx_2(t_\mathrm{i}) - QT_{0,2}(t_\mathrm{i}) - QT_{1,1}(t_\mathrm{i})$):

If $QT_{1,2,\mathrm{x,x1}}(t_\mathrm{i}) = 0$ or $Qx_2(t_\mathrm{i}) - QT_{0,2}(t_\mathrm{i}) - QT_{1,1}(t_\mathrm{i}) = 0$, then

$QT_{1,2}(t_\mathrm{i}) = 0,$

otherwise,

$$QT_{1,2}(t_\mathrm{i}) = min\left(\frac{QT_{1,2,\mathrm{x,x1}}(t_\mathrm{i})\big(Qx_2(t_\mathrm{i}) - QT_{0,2}(t_\mathrm{i}) - QT_{1,1}(t_\mathrm{i})\big)}{Q\tilde{T}_{0,2}(t_\mathrm{i}) - QT_{0,2,\mathrm{x,x1}}(t_\mathrm{i}) + Q\tilde{T}_1(t_\mathrm{i}) - QT_{1,1,\mathrm{x}}(t_\mathrm{i}) + Q\tilde{T}_{2,2}(t_\mathrm{i})}, QT_{1,2,\mathrm{x,x1}}(t_\mathrm{i})\right).$$

Determination of the density of the $T_{2,1}$-type protocells surviving the competition ($QT_{2,1}(t_\mathrm{i})$)

This density ($QT_{2,1,\mathrm{x}}(t_\mathrm{i})$) is that of the $T_{2,1}$-type protocells which get x (protocells of density $Q\tilde{T}_2(t_\mathrm{i})$ compete for x of density $Qx(t_\mathrm{i}) - QT_{0,1}(t_\mathrm{i}) - QT_{0,2,\mathrm{x}}(t_\mathrm{i}) - QT_{1,1,\mathrm{x}}(t_\mathrm{i}) - QT_{1,2,\mathrm{x}}(t_\mathrm{i})$):

If $Q\tilde{T}_{2,1}(t_\mathrm{i}) = 0$ or $Qx(t_\mathrm{i}) - QT_{0,1}(t_\mathrm{i}) - QT_{0,2,\mathrm{x}}(t_\mathrm{i}) - QT_{1,1,\mathrm{x}}(t_\mathrm{i}) - QT_{1,2,\mathrm{x}}(t_\mathrm{i}) = 0$, then

$QT_{2,1,\mathrm{x}}(t_\mathrm{i}) = 0,$

otherwise,

$$QT_{2,1,\mathrm{x}}(t_\mathrm{i}) = min\left(\frac{Q\tilde{T}_{2,1}(t_\mathrm{i})\big(Qx(t_\mathrm{i}) - QT_{0,1}(t_\mathrm{i}) - QT_{0,2,\mathrm{x}}(t_\mathrm{i}) - QT_{1,1,\mathrm{x}}(t_\mathrm{i}) - QT_{1,2,\mathrm{x}}(t_\mathrm{i})\big)}{Q\tilde{T}_2(t_\mathrm{i})}, Q\tilde{T}_{2,1}(t_\mathrm{i})\right).$$

This density ($QT_{2,1}(t_{\mathrm{i}})$) is that of the $T_{2,1}$-type protocells which get x and x_1 (protocells of density $Q\tilde{T}_{0,2}(t_{\mathrm{i}}) - QT_{0,2,\mathrm{x}}(t_{\mathrm{i}}) + Q\tilde{T}_{1,2}(t_{\mathrm{i}}) - QT_{1,2,\mathrm{x}}(t_{\mathrm{i}}) + Q\tilde{T}_2(t_{\mathrm{i}})$ compete for x_1 of density $Qx_1(t_{\mathrm{i}}) - QT_{0,2,\mathrm{x,x1}}(t_{\mathrm{i}}) - QT_{1,2,\mathrm{x,x1}}(t_{\mathrm{i}})$):

If $QT_{2,1,\mathrm{x}}(t_{\mathrm{i}}) = 0$ or $Qx_1(t_{\mathrm{i}}) - QT_{0,2,\mathrm{x,x1}}(t_{\mathrm{i}}) - QT_{1,2,\mathrm{x,x1}}(t_{\mathrm{i}}) = 0$, then

$QT_{2,1}(t_{\mathrm{i}}) = 0$,

otherwise,

$$QT_{2,1}(t_{\mathrm{i}}) = min\left(\frac{QT_{2,1,\mathrm{x}}(t_{\mathrm{i}})\left(Qx_1(t_{\mathrm{i}}) - QT_{0,2,\mathrm{x,x1}}(t_{\mathrm{i}}) - QT_{1,2,\mathrm{x,x1}}(t_{\mathrm{i}})\right)}{Q\tilde{T}_{0,2}(t_{\mathrm{i}}) - QT_{0,2,\mathrm{x}}(t_{\mathrm{i}}) + Q\tilde{T}_{1,2}(t_{\mathrm{i}}) - QT_{1,2,\mathrm{x}}(t_{\mathrm{i}}) + Q\tilde{T}_2(t_{\mathrm{i}})}, QT_{2,1,\mathrm{x}}(t_{\mathrm{i}})\right).$$

Determination of the density of the $T_{2,2}$-type protocells surviving the competition ($QT_{2,2}(t_{\mathrm{i}})$)

This density ($QT_{2,2,\mathrm{x}}(t_{\mathrm{i}})$) is that of the $T_{2,2}$-type protocells which get nutrient x (there is still $Qx(t_{\mathrm{i}}) - QT_{0,1}(t_i) - QT_{0,2,\mathrm{x}}(t_i) - QT_{1,1,\mathrm{x}}(t_i) - QT_{1,2,\mathrm{x}}(t_i) - QT_{2,1,\mathrm{x}}(t_i)$ of x, which is completely left for them):

$$QT_{2,2,\mathrm{x}}(t_{\mathrm{i}}) = min\left(Qx(t_{\mathrm{i}}) - QT_{0,1}(t_i) - QT_{0,2,\mathrm{x}}(t_i) - QT_{1,1,\mathrm{x}}(t_i) - QT_{1,2,\mathrm{x}}(t_i) - QT_{2,1,\mathrm{x}}(t_i), Q\tilde{T}_{2,2}(t_{\mathrm{i}})\right).$$

This density ($QT_{2,2,\mathrm{x,x1}}(t_{\mathrm{i}})$) is that of the $T_{2,2}$-type protocells which get x and x_1 (protocells of density $Q\tilde{T}_{0,2}(t_{\mathrm{i}}) - QT_{0,2,\mathrm{x}}(t_{\mathrm{i}}) + Q\tilde{T}_{1,2}(t_{\mathrm{i}}) - QT_{1,2,\mathrm{x}}(t_{\mathrm{i}}) + Q\tilde{T}_2(t_{\mathrm{i}}) - QT_{2,1,\mathrm{x}}(t_{\mathrm{i}})$ compete for x_1 of density $Qx_1(t_{\mathrm{i}}) - QT_{0,2,\mathrm{x,x1}}(t_{\mathrm{i}}) - QT_{1,2,\mathrm{x,x1}}(t_{\mathrm{i}}) - QT_{2,1}(t_{\mathrm{i}})$):

If $QT_{2,2,\mathrm{x}}(t_{\mathrm{i}}) = 0$ or $Qx_1(t_{\mathrm{i}}) - QT_{0,2,\mathrm{x,x1}}(t_{\mathrm{i}}) - QT_{1,2,\mathrm{x,x1}}(t_{\mathrm{i}}) - QT_{2,1}(t_{\mathrm{i}}) = 0$, then

$QT_{2,2,\mathrm{x,x1}}(t_{\mathrm{i}}) = 0$,

otherwise,

$$QT_{2,2,\mathrm{x,x1}}(t_{\mathrm{i}}) = min\left(\frac{QT_{2,2,\mathrm{x}}(t_{\mathrm{i}})\left(Qx_1(t_{\mathrm{i}}) - QT_{0,2,\mathrm{x,x1}}(t_{\mathrm{i}}) - QT_{1,2,\mathrm{x,x1}}(t_{\mathrm{i}}) - QT_{2,1}(t_{\mathrm{i}})\right)}{Q\tilde{T}_{0,2}(t_{\mathrm{i}}) - QT_{0,2,\mathrm{x}}(t_{\mathrm{i}}) + Q\tilde{T}_{1,2}(t_{\mathrm{i}}) - QT_{1,2,\mathrm{x}}(t_{\mathrm{i}}) + Q\tilde{T}_2(t_{\mathrm{i}}) - QT_{2,1,\mathrm{x}}(t_{\mathrm{i}})}, QT_{2,2,\mathrm{x}}(t_{\mathrm{i}})\right).$$

This density ($QT_{2,2}(t_{\mathrm{i}})$) is that of the $T_{2,2}$-type protocells which get x, x_1 and x_2 (protocells of density $Q\tilde{T}_{0,2}(t_{\mathrm{i}}) - QT_{0,2,\mathrm{x,x1}}(t_{\mathrm{i}}) + Q\tilde{T}_1(t_{\mathrm{i}}) - QT_{1,1,\mathrm{x}}(t_{\mathrm{i}}) - QT_{1,2,\mathrm{x,x1}}(t_{\mathrm{i}}) + Q\tilde{T}_{2,2}(t_{\mathrm{i}})$ compete for x_2 of density $Qx_2(t_{\mathrm{i}}) - QT_{0,2}(t_{\mathrm{i}}) - QT_1(t_{\mathrm{i}})$):

If $QT_{2,2,\mathrm{x,x1}}(t_{\mathrm{i}}) = 0$ or $Qx_2(t_{\mathrm{i}}) - QT_{0,2}(t_{\mathrm{i}}) - QT_1(t_{\mathrm{i}}) = 0$, then

$QT_{2,2}(t_{\mathrm{i}}) = 0$,

otherwise,

$$QT_{2,2}(t_{\mathrm{i}}) = min\left(\frac{QT_{2,2,\mathrm{x,x1}}(t_{\mathrm{i}})\left(Qx_2(t_{\mathrm{i}}) - QT_{0,2}(t_{\mathrm{i}}) - QT_1(t_{\mathrm{i}})\right)}{Q\tilde{T}_{0,2}(t_{\mathrm{i}}) - QT_{0,2,\mathrm{x,x1}}(t_{\mathrm{i}}) + Q\tilde{T}_1(t_{\mathrm{i}}) - QT_{1,1,\mathrm{x}}(t_{\mathrm{i}}) - QT_{1,2,\mathrm{x,x1}}(t_{\mathrm{i}}) + Q\tilde{T}_{2,2}(t_{\mathrm{i}})}, QT_{2,2,\mathrm{x,x1}}(t_{\mathrm{i}})\right).$$

This density of x is not absorbed by the protocells in the competition (surplus of x):

$$Qx_{\mathrm{f}}(t_{\mathrm{i}}) = max\left(Qx(t_{\mathrm{i}}) - Q\tilde{T}(t_{\mathrm{i}}), 0\right).$$

This is the density of x absorbed by the not surviving protocells and, consequently, get out to the environment by the next modeling time point:

$$Qx_{\mathrm{h}}(t_{\mathrm{i}}) = min\left(Qx(t_{\mathrm{i}}) - QT_{0,1}(t_{\mathrm{i}}), Q\tilde{T}_{0,2}(t_{\mathrm{i}}) + Q\tilde{T}_1(t_{\mathrm{i}}) + Q\tilde{T}_2(t_{\mathrm{i}})\right) - QT_{0,2}(t_{\mathrm{i}}) - QT_1(t_{\mathrm{i}}) - -QT_2(t_{\mathrm{i}}).$$

This density of x_1 is not absorbed by the protocells in the competition (surplus of x_1):

$Qx_{1,\mathrm{f}}(t_\mathrm{i}) = max\big(Qx_1(t_\mathrm{i}) - Q\tilde{T}_{0,2}(t_\mathrm{i}) - Q\tilde{T}_{1,2}(t_\mathrm{i}) - Q\tilde{T}_2(t_\mathrm{i}), 0\big).$

This is the density of x_1 absorbed by the not surviving protocells and, consequently, get out to the environment by the next modeling time point:

$Qx_{1,\mathrm{h}}(t_\mathrm{i}) = min\left(Qx_1(t_\mathrm{i}), Q\tilde{T}_{0,2}(t_\mathrm{i}) + Q\tilde{T}_{1,2}(t_\mathrm{i}) + Q\tilde{T}_2(t_\mathrm{i})\right) - QT_{0,2}(t_\mathrm{i}) - QT_{1,2}(t_\mathrm{i}) - QT_2(t_\mathrm{i}).$

This density of x_2 is not absorbed by the protocells in the competition (surplus of x_2):

$Qx_{2,\mathrm{f}}(t_\mathrm{i}) = max\big(Qx_2(t_\mathrm{i}) - Q\tilde{T}_{0,2}(t_\mathrm{i}) - Q\tilde{T}_1(t_\mathrm{i}) - Q\tilde{T}_{2,2}(t_\mathrm{i}), 0\big).$

This is the density of x_2 absorbed by the not surviving protocells and, consequently, get out to the environment by the next modeling time point:

$Qx_{2,\mathrm{h}}(t_\mathrm{i}) = min\left(Qx_2(t_\mathrm{i}), Q\tilde{T}_{0,2}(t_\mathrm{i}) + Q\tilde{T}_1(t_\mathrm{i}) + Q\tilde{T}_{2,2}(t_\mathrm{i})\right) - QT_{0,2}(t_\mathrm{i}) - QT_1(t_\mathrm{i}) - QT_{2,2}(t_\mathrm{i}).$

B. Supplementary calculations for the equilibrium

Two operation cases, VI.1/B/d+VI.2 and VII.1/B/h+VII.2 correspond to our equilibrium analysis. Their describing systems of equalities are listed as follows. (The zero variables like $QT_{1,1} = QT_{1,2} = QT_{2,1} = QT_{2,2} = 0$ are not displayed in the systems.)

***VI.1/B/d*+VI.2** (where $i = 1, 3, 5, ...$)

$Q\tilde{T}_{0,1}(t_\mathrm{i}) = QT_{0,1}(t_{\mathrm{i}-1}),$

$Q\tilde{T}_{0,2}(t_\mathrm{i}) = QT_{0,2}(t_{\mathrm{i}-1}),$

$Qx(t_\mathrm{i}) = Qx_\mathrm{f}(t_{\mathrm{i}-1}) + Qx_\mathrm{h}(t_{\mathrm{i}-1}) + b(t_\mathrm{i} - t_{\mathrm{i}-1}),$

$Qx_1(t_\mathrm{i}) = max\big(Qx_{1,\mathrm{f}}(t_{\mathrm{i}-1}) + Qx_{1,\mathrm{h}}(t_{\mathrm{i}-1}) + b_1(t_\mathrm{i} - t_{\mathrm{i}-1}), 0\big),$

$Qx_2(t_\mathrm{i}) = max\big(Qx_{2,\mathrm{f}}(t_{\mathrm{i}-1}) + Qx_{2,\mathrm{h}}(t_{\mathrm{i}-1}) + b_2(t_\mathrm{i} - t_{\mathrm{i}-1}), 0\big),$

$QT_{0,1}(t_\mathrm{i}) = Q\tilde{T}_{0,1}(t_\mathrm{i}),$

$QT_{0,2}(t_\mathrm{i}) = Q\tilde{T}_{0,2}(t_\mathrm{i}),$

$Qx_\mathrm{f}(t_\mathrm{i}) = max(Qx(t_\mathrm{i}), 0),$

$Qx_\mathrm{h}(t_\mathrm{i}) = 0,$

$Qx_{1,\mathrm{f}}(t_\mathrm{i}) = max(Qx_1(t_\mathrm{i}), 0),$

$Qx_{1,\mathrm{h}}(t_\mathrm{i}) = 0,$

$Qx_{2,\mathrm{f}}(t_\mathrm{i}) = max(Qx_2(t_\mathrm{i}), 0),$

$Qx_{2,\mathrm{h}}(t_\mathrm{i}) = 0$

***VII.1/B/h*+VII.2** (where $i = 2, 4, 6, ...$)

$Qx(t_\mathrm{i}) = Qx_\mathrm{f}(t_{\mathrm{i}-1}) + Qx_\mathrm{h}(t_{\mathrm{i}-1}) + b(t_\mathrm{i} - t_{\mathrm{i}-1}),$

$Q\tilde{T}_{0,1}(t_\mathrm{i}) = QT_0\left(h_{\tau_0}(i-1)\right),$

$Q\tilde{T}_{0,2}(t_\mathrm{i}) = QT_{0,1}\left(h_{\tau_0}(i-1)\right),$

$Qx_1(t_\mathrm{i}) = max\left(Qx_{1,\mathrm{f}}(t_{\mathrm{i}-1}) + Qx_{1,\mathrm{h}}(t_{\mathrm{i}-1}) + b_1(t_\mathrm{i} - t_{\mathrm{i}-1}) + QT_0\left(h_{\tau_0}(i-1)\right), 0\right),$

$Qx_2(t_\mathrm{i}) = max\left(Qx_{2,\mathrm{f}}(t_{\mathrm{i}-1}) + Qx_{2,\mathrm{h}}(t_{\mathrm{i}-1}) + b_2(t_\mathrm{i} - t_{\mathrm{i}-1}) + QT_0\left(h_{\tau_0}(i-1)\right), 0\right),$

$$QT_{0,1}(t_i) = min\left(\frac{Q\tilde{T}_{0,1}(t_i)Qx(t_i)}{Q\tilde{T}_0(t_i)}, Q\tilde{T}_{0,1}(t_i)\right),$$

$$QT_{0,2,x}(t_i) = min\left(Qx(t_i) - QT_{0,1}(t_i), Q\tilde{T}_{0,2}(t_i)\right),$$

$$QT_{0,2,x,x1}(t_i) = min\left(\frac{QT_{0,2,x}(t_i)Qx_1(t_i)}{Q\tilde{T}_{0,2}(t_i)}, QT_{0,2,x}(t_i)\right),$$

$$QT_{0,2}(t_i) = min\left(\frac{QT_{0,2,x,x1}(t_i)Qx_2(t_i)}{Q\tilde{T}_{0,2}(t_i)}, QT_{0,2,x,x1}(t_i)\right),$$

$$Qx_f(t_i) = max\left(Qx(t_i) - Q\tilde{T}_0(t_i), 0\right),$$

$$Qx_h(t_i) = min\left(Qx(t_i) - QT_{0,1}(t_i), Q\tilde{T}_{0,2}(t_i)\right) - QT_{0,2}(t_i),$$

$$Qx_{1,f}(t_i) = max\left(Qx_1(t_i) - Q\tilde{T}_{0,2}(t_i), 0\right),$$

$$Qx_{1,h}(t_i) = min\left(Qx_1(t_i), Q\tilde{T}_{0,2}(t_i)\right) - QT_{0,2}(t_i),$$

$$Qx_{2,f}(t_i) = max\left(Qx_2(t_i) - Q\tilde{T}_{0,2}(t_i), 0\right),$$

$$Qx_{2,h}(t_i) = min\left(Qx_2(t_i), Q\tilde{T}_{0,2}(t_i)\right) - QT_{0,2}(t_i)$$

It can be seen that the necessary conditions for changing from one of the two operation cases to the other one always hold. By shifting system VII.1/B/h+VII.2 (where $i = 2, 4, 6, ...$) in time by one step, we obtain the following system:

$$Qx(t_{i+1}) = Qx_f(t_i) + Qx_h(t_i) + b(t_{i+1} - t_i),$$

$$Q\tilde{T}_{0,1}(t_{i+1}) = QT_0\left(h_{\tau_0}(i)\right),$$

$$Q\tilde{T}_{0,2}(t_{i+1}) = QT_{0,1}\left(h_{\tau_0}(i)\right),$$

$$Qx_1(t_{i+1}) = max\left(Qx_{1,f}(t_i) + Qx_{1,h}(t_i) + b_1(t_{i+1} - t_i) + QT_0\left(h_{\tau_0}(i)\right), 0\right),$$

$$Qx_2(t_{i+1}) = max\left(Qx_{2,f}(t_i) + Qx_{2,h}(t_i) + b_2(t_{i+1} - t_i) + QT_0\left(h_{\tau_0}(i)\right), 0\right),$$

$$QT_{0,1}(t_{i+1}) = min\left(\frac{Q\tilde{T}_{0,1}(t_{i+1})Qx(t_{i+1})}{Q\tilde{T}_0(t_i+1)}, Q\tilde{T}_{0,1}(t_{i+1})\right),$$

$$QT_{0,2,x}(t_{i+1}) = min\left(Qx(t_{i+1}) - QT_{0,1}(t_{i+1}), Q\tilde{T}_{0,2}(t_{i+1})\right),$$

$$QT_{0,2,x,x1}(t_{i+1}) = min\left(\frac{QT_{0,2,x}(t_{i+1})Qx_1(t_{i+1})}{Q\tilde{T}_{0,2}(t_{i+1})}, QT_{0,2,x}(t_{i+1})\right),$$

$$QT_{0,2}(t_{i+1}) = min\left(\frac{QT_{0,2,x,x1}(t_{i+1})Qx_2(t_{i+1})}{Q\tilde{T}_{0,2}(t_{i+1})}, QT_{0,2,x,x1}(t_{i+1})\right),$$

$$Qx_f(t_{i+1}) = max\left(Qx(t_{i+1}) - Q\tilde{T}_0(t_{i+1}), 0\right),$$

$$Qx_h(t_{i+1}) = min\left(Qx(t_{i+1}) - QT_{0,1}(t_{i+1}), Q\tilde{T}_{0,2}(t_{i+1})\right) - QT_{0,2}(t_{i+1}),$$

$$Qx_{1,f}(t_{i+1}) = max\left(Qx_1(t_{i+1}) - Q\tilde{T}_{0,2}(t_{i+1}), 0\right),$$

$$Qx_{1,h}(t_{i+1}) = min\left(Qx_1(t_{i+1}), Q\tilde{T}_{0,2}(t_{i+1})\right) - QT_{0,2}(t_{i+1}),$$

$$Qx_{2,f}(t_{i+1}) = max\left(Qx_2(t_{i+1}) - Q\tilde{T}_{0,2}(t_{i+1}), 0\right),$$

$Qx_{2,\mathrm{h}}(t_{\mathrm{i+1}}) = min\left(Qx_2(t_{\mathrm{i+1}}), Q\tilde{T}_{0,2}(t_{\mathrm{i+1}})\right) - QT_{0,2}(t_{\mathrm{i+1}})$.

Using the fact that $h_{\tau_0}(i) = t_{\mathrm{i+1}} - 1 = t_{\mathrm{i-1}}$ $(i = 2, 4, 6, ...)$, the shifted system VII.1/B/*h*+VII.2 (where $i = 2, 4, 6, ...$) takes the form

$Qx(t_{\mathrm{i+1}}) = Qx_{\mathrm{f}}(t_{\mathrm{i}}) + Qx_{\mathrm{h}}(t_{\mathrm{i}}) + b(t_{\mathrm{i+1}} - t_{\mathrm{i}})$,

$Q\tilde{T}_{0,1}(t_{\mathrm{i+1}}) = QT_0(t_{i-1})$,

$Q\tilde{T}_{0,2}(t_{\mathrm{i+1}}) = QT_{0,1}(t_{i-1})$,

$Qx_1(t_{\mathrm{i+1}}) = max\left(Qx_{1,\mathrm{f}}(t_{\mathrm{i}}) + Qx_{1,\mathrm{h}}(t_{\mathrm{i}}) + b_1(t_{\mathrm{i+1}} - t_{\mathrm{i}}) + QT_0(t_{i-1}), 0\right)$,

$Qx_2(t_{\mathrm{i+1}}) = max\left(Qx_{2,\mathrm{f}}(t_{\mathrm{i}}) + Qx_{2,\mathrm{h}}(t_{\mathrm{i}}) + b_2(t_{\mathrm{i+1}} - t_{\mathrm{i}}) + QT_0(t_{i-1}), 0\right)$,

$QT_{0,1}(t_{\mathrm{i+1}}) = min\left(\frac{Q\tilde{T}_{0,1}(t_{\mathrm{i+1}})Qx(t_{\mathrm{i+1}})}{Q\tilde{T}_0(t_{\mathrm{i}}+1)}, Q\tilde{T}_{0,1}(t_{\mathrm{i+1}})\right)$,

$QT_{0,2,\mathrm{x}}(t_{\mathrm{i+1}}) = min\left(Qx(t_{\mathrm{i+1}}) - QT_{0,1}(t_{\mathrm{i+1}}), Q\tilde{T}_{0,2}(t_{\mathrm{i+1}})\right)$,

$QT_{0,2,\mathrm{x,x1}}(t_{\mathrm{i+1}}) = min\left(\frac{QT_{0,2,\mathrm{x}}(t_{\mathrm{i+1}})Qx_1(t_{\mathrm{i+1}})}{Q\tilde{T}_{0,2}(t_{\mathrm{i+1}})}, QT_{0,2,\mathrm{x}}(t_{\mathrm{i+1}})\right)$,

$QT_{0,2}(t_{\mathrm{i+1}}) = min\left(\frac{QT_{0,2,\mathrm{x,x1}}(t_{\mathrm{i+1}})Qx_2(t_{\mathrm{i+1}})}{Q\tilde{T}_{0,2}(t_{\mathrm{i+1}})}, QT_{0,2,\mathrm{x,x1}}(t_{\mathrm{i+1}})\right)$,

$Qx_{\mathrm{f}}(t_{\mathrm{i+1}}) = max\left(Qx(t_{\mathrm{i+1}}) - Q\tilde{T}_0(t_{\mathrm{i+1}}), 0\right)$,

$Qx_{\mathrm{h}}(t_{\mathrm{i+1}}) = min\left(Qx(t_{\mathrm{i+1}}) - QT_{0,1}(t_{\mathrm{i+1}}), Q\tilde{T}_{0,2}(t_{\mathrm{i+1}})\right) - QT_{0,2}(t_{\mathrm{i+1}})$,

$Qx_{1,\mathrm{f}}(t_{\mathrm{i+1}}) = max\left(Qx_1(t_{\mathrm{i+1}}) - Q\tilde{T}_{0,2}(t_{\mathrm{i+1}}), 0\right)$,

$Qx_{1,\mathrm{h}}(t_{\mathrm{i+1}}) = min\left(Qx_1(t_{\mathrm{i+1}}), Q\tilde{T}_{0,2}(t_{\mathrm{i+1}})\right) - QT_{0,2}(t_{\mathrm{i+1}})$,

$Qx_{2,\mathrm{f}}(t_{\mathrm{i+1}}) = max\left(Qx_2(t_{\mathrm{i+1}}) - Q\tilde{T}_{0,2}(t_{\mathrm{i+1}}), 0\right)$,

$Qx_{2,\mathrm{h}}(t_{\mathrm{i+1}}) = min\left(Qx_2(t_{\mathrm{i+1}}), Q\tilde{T}_{0,2}(t_{\mathrm{i+1}})\right) - QT_{0,2}(t_{\mathrm{i+1}})$.

The iteration of the operation cases produces the following system of equations, as the mentioned time-contracted model (for $i = 2, 4, 6$ ...):

$Qx(t_{\mathrm{i+1}}) = Qx_{\mathrm{f}}(t_{\mathrm{i-1}}) + Qx_{\mathrm{h}}(t_{\mathrm{i-1}}) + b(t_{\mathrm{i+1}} - t_{\mathrm{i-1}})$,

$Q\tilde{T}_{0,1}(t_{\mathrm{i+1}}) = QT_0(t_{\mathrm{i-1}})$,

$Q\tilde{T}_{0,2}(t_{\mathrm{i+1}}) = QT_{0,1}(t_{\mathrm{i-1}})$,

$Qx_1(t_{\mathrm{i+1}}) = Qx_{1,\mathrm{f}}(t_{\mathrm{i-1}}) + Qx_{1,\mathrm{h}}(t_{\mathrm{i-1}}) + b_1(t_{\mathrm{i+1}} - t_{\mathrm{i-1}}) + QT_0(t_{\mathrm{i-1}})$,

$Qx_2(t_{\mathrm{i+1}}) = Qx_{2,\mathrm{f}}(t_{\mathrm{i-1}}) + Qx_{2,\mathrm{h}}(t_{\mathrm{i-1}}) + b_2(t_{\mathrm{i+1}} - t_{\mathrm{i-1}}) + QT_0(t_{\mathrm{i-1}})$,

$QT_{0,1}(t_{\mathrm{i+1}}) = \frac{Q\tilde{T}_{0,1}(t_{\mathrm{i+1}})Qx(t_{\mathrm{i+1}})}{Q\tilde{T}_0(t_{\mathrm{i+1}})}$,

$QT_{0,2,\mathrm{x}}(t_{\mathrm{i+1}}) = Qx(t_{\mathrm{i+1}}) - QT_{0,1}(t_{\mathrm{i+1}})$,

$QT_{0,2,\mathrm{x,x1}}(t_{\mathrm{i+1}}) = QT_{0,2,\mathrm{x}}(t_{\mathrm{i+1}})$,

$QT_{0,2}(t_{\mathrm{i+1}}) = Qx(t_{\mathrm{i+1}}) - QT_{0,1}(t_{\mathrm{i+1}})$,

$Qx_{\mathrm{f}}(t_{\mathrm{i+1}}) = 0$,

$Qx_{\mathrm{h}}(t_{\mathrm{i+1}}) = Qx(t_{\mathrm{i+1}}) - QT_0(t_{\mathrm{i+1}})$,

$Qx_{1,\mathrm{f}}(t_{\mathrm{i+1}}) = Qx_1(t_{\mathrm{i+1}}) - Q\tilde{T}_{0,2}(t_{\mathrm{i+1}}),$

$Qx_{1,\mathrm{h}}(t_{\mathrm{i+1}}) = Q\tilde{T}_{0,2}(t_{\mathrm{i+1}}) - QT_{0,2}(t_{\mathrm{i+1}}),$

$Qx_{2,\mathrm{f}}(t_{\mathrm{i+1}}) = Qx_2(t_{\mathrm{i+1}}) - Q\tilde{T}_{0,2}(t_{\mathrm{i+1}}),$

$Qx_{2,\mathrm{h}}(t_{\mathrm{i+1}}) = Q\tilde{T}_{0,2}(t_{\mathrm{i+1}}) - QT_{0,2}(t_{\mathrm{i+1}}).$

Since $t_{\mathrm{i}} - t_{\mathrm{i-1}} = 0.5$ ($i = 1, 2, 3, \ldots$) and $b_1 = b_2$, furthermore, by using the expressions for $Q\tilde{T}_{0,1}$ and $Q\tilde{T}_{0,2}$ in the equation of $QT_{0,1}$, it yields the following system of equations:

$Qx(t_{\mathrm{i+1}}) = Qx_{\mathrm{f}}(t_{\mathrm{i-1}}) + Qx_{\mathrm{h}}(t_{\mathrm{i-1}}) + b,$

$Q\tilde{T}_{0,1}(t_{\mathrm{i+1}}) = QT_0(t_{\mathrm{i-1}}),$

$Q\tilde{T}_{0,2}(t_{\mathrm{i+1}}) = QT_{0,1}(t_{\mathrm{i-1}}),$

$Qx_1(t_{\mathrm{i+1}}) = Qx_{1,\mathrm{f}}(t_{\mathrm{i-1}}) + Qx_{1,\mathrm{h}}(t_{\mathrm{i-1}}) + b_1 + QT_0(t_{\mathrm{i-1}}),$

$Qx_2(t_{\mathrm{i+1}}) = Qx_{2,\mathrm{f}}(t_{\mathrm{i-1}}) + Qx_{2,\mathrm{h}}(t_{\mathrm{i-1}}) + b_1 + QT_0(t_{\mathrm{i-1}}),$

$QT_{0,1}(t_{\mathrm{i+1}}) = \frac{QT_0(t_{\mathrm{i+1}})Qx(t_{\mathrm{i+1}})}{QT_{0,1}(t_{\mathrm{i-1}}) + QT_0(t_{\mathrm{i-1}})},$

$QT_{0,2,\mathrm{x}}(t_{\mathrm{i+1}}) = Qx(t_{\mathrm{i+1}}) - QT_{0,1}(t_{\mathrm{i+1}}),$

$QT_{0,2,\mathrm{x,x1}}(t_{\mathrm{i+1}}) = QT_{0,2,\mathrm{x}}(t_{\mathrm{i+1}}),$

$QT_{0,2}(t_{\mathrm{i+1}}) = Qx(t_{\mathrm{i+1}}) - QT_{0,1}(t_{\mathrm{i+1}}),$

$Qx_{\mathrm{f}}(t_{\mathrm{i+1}}) = 0,$

$Qx_{\mathrm{h}}(t_{\mathrm{i+1}}) = Qx(t_{\mathrm{i+1}}) - QT_0(t_{\mathrm{i+1}}),$

$Qx_{1,\mathrm{f}}(t_{\mathrm{i+1}}) = Qx_1(t_{\mathrm{i+1}}) - Q\tilde{T}_{0,2}(t_{\mathrm{i+1}}),$

$Qx_{1,\mathrm{h}}(t_{\mathrm{i+1}}) = Q\tilde{T}_{0,2}(t_{\mathrm{i+1}}) - QT_{0,2}(t_{\mathrm{i+1}}),$

$Qx_{2,\mathrm{f}}(t_{\mathrm{i+1}}) = Qx_2(t_{\mathrm{i+1}}) - Q\tilde{T}_{0,2}(t_{\mathrm{i+1}}),$

$Qx_{2,\mathrm{h}}(t_{\mathrm{i+1}}) = Q\tilde{T}_{0,2}(t_{\mathrm{i+1}}) - QT_{0,2}(t_{\mathrm{i+1}}).$

Taking into account the equations for Qx_{f} and for Qx_{h} gives the opportunity to express Qx with the aid of $QT_{0,1}$ and $QT_{0,2}$:

$Qx(t_{\mathrm{i+1}}) = Qx(t_{\mathrm{i-1}}) - QT_0(t_{\mathrm{i-1}}) + b,$

$Q\tilde{T}_{0,1}(t_{\mathrm{i+1}}) = QT_0(t_{\mathrm{i-1}}),$

$Q\tilde{T}_{0,2}(t_{\mathrm{i+1}}) = QT_{0,1}(t_{\mathrm{i-1}}),$

$Qx_1(t_{\mathrm{i+1}}) = Qx_{1,f}(t_{\mathrm{i-1}}) + Qx_{1,h}(t_{\mathrm{i-1}}) + b_1 + QT_0(t_{\mathrm{i-1}}),$

$Qx_2(t_{\mathrm{i+1}}) = Qx_{2,f}(t_{\mathrm{i-1}}) + Qx_{2,h}(t_{\mathrm{i-1}}) + b_1 + QT_0(t_{\mathrm{i-1}}),$

$QT_{0,1}(t_{\mathrm{i+1}}) = \frac{QT_0(t_{\mathrm{i+1}})Qx(t_{\mathrm{i+1}})}{QT_{0,1}(t_{\mathrm{i-1}}) + QT_0(t_{\mathrm{i-1}})},$

$QT_{0,2,\mathrm{x}}(t_{\mathrm{i+1}}) = Qx(t_{\mathrm{i+1}}) - QT_{0,1}(t_{\mathrm{i+1}}),$

$QT_{0,2,\mathrm{x,x1}}(t_{\mathrm{i+1}}) = QT_{0,2,\mathrm{x}}(t_{\mathrm{i+1}}),$

$QT_{0,2}(t_{\mathrm{i+1}}) = Qx(t_{\mathrm{i+1}}) - QT_{0,1}(t_{\mathrm{i+1}}),$

$Qx_{\mathrm{f}}(t_{\mathrm{i+1}}) = 0,$

$Qx_{\mathrm{h}}(t_{\mathrm{i+1}}) = Qx(t_{\mathrm{i+1}}) - QT_0(t_{\mathrm{i+1}}),$

$Qx_{1,\mathrm{f}}(t_{\mathrm{i+1}}) = Qx_1(t_{\mathrm{i+1}}) - Q\tilde{T}_{0,2}(t_{\mathrm{i+1}}),$

$Qx_{1,\mathrm{h}}(t_{\mathrm{i+1}}) = Q\tilde{T}_{0,2}(t_{\mathrm{i+1}}) - QT_{0,2}(t_{\mathrm{i+1}}),$

$Qx_{2,\mathrm{f}}(t_{\mathrm{i+1}}) = Qx_2(t_{\mathrm{i+1}}) - Q\tilde{T}_{0,2}(t_{\mathrm{i+1}}),$

$Qx_{2,\mathrm{h}}(t_{\mathrm{i+1}}) = Q\tilde{T}_{0,2}(t_{\mathrm{i+1}}) - QT_{0,2}(t_{\mathrm{i+1}}).$

The below system of equations is resulted for the "main" variables. $Qx_{1,\mathrm{h}}$ and $Qx_{2,\mathrm{h}}$ are not listed since they can be expressed directly from the "main" variables (see the above equalities).

$Qx(t_{\mathrm{i+1}}) = Qx(t_{\mathrm{i-1}}) - QT_0(t_{\mathrm{i-1}}) + b,$

$QT_{0,1}(t_{\mathrm{i+1}}) = \frac{QT_0(t_{\mathrm{i+1}})Qx(t_{\mathrm{i+1}})}{QT_{0,1}(t_{\mathrm{i-1}})+QT_0(t_{\mathrm{i-1}})},$

$QT_{0,2}(t_{\mathrm{i+1}}) = Qx(t_{\mathrm{i+1}}) - QT_{0,1}(t_{\mathrm{i+1}}),$

$Qx_1(t_{\mathrm{i+1}}) = Qx_1(t_{\mathrm{i-1}}) + QT_{0,1}(t_{\mathrm{i-1}}) + b_1,$

$Qx_2(t_{\mathrm{i+1}}) = Qx_2(t_{\mathrm{i-1}}) + QT_{0,2}(t_{\mathrm{i-1}}) + b_1,$

where Qx_1 and Qx_2 are also expressed. It can be seen that if $b_1 \geq 0$, then Qx_1 and Qx_2 tend to infinity (as i tends to infinity) according to our initial assumption, so the system to be examined from the point of view of stability is

$Qx(t_{\mathrm{i+1}}) = Qx(t_{\mathrm{i-1}}) - QT_0(t_{\mathrm{i-1}}) + b,$

$QT_{0,1}(t_{\mathrm{i+1}}) = \frac{QT_0(t_{\mathrm{i+1}})Qx(t_{\mathrm{i+1}})}{QT_{0,1}(t_{\mathrm{i-1}})+QT_0(t_{\mathrm{i-1}})},$

$QT_{0,2}(t_{\mathrm{i+1}}) = Qx(t_{\mathrm{i+1}}) - QT_{0,1}(t_{\mathrm{i+1}}).$

Based on the first and the third equation of this system it follows that $Qx(t_{\mathrm{i+1}}) = b$ and $b = QT_0(t_{\mathrm{i+1}})$, therefore the system takes the form:

$$Qx(t_{\mathrm{i+1}}) = b, \tag{B.1}$$

$$QT_{0,1}(t_{\mathrm{i+1}}) = \frac{b^2}{QT_{0,1}(t_{\mathrm{i-1}})+b}, \tag{B.2}$$

$$QT_{0,2}(t_{\mathrm{i+1}}) = b - QT_{0,1}(t_{\mathrm{i+1}}). \tag{B.3}$$

To obtain the possible equilibria, one should solve the following system of algebraic equations:

$Qx^* = b,$

$QT^*_{0,1} = \frac{b^2}{QT^*_{0,1}+b},$

$QT^*_{0,2} = b - QT^*_{0,1}.$

The only possible solution for the second equation is

$QT^*_{0,1} = \frac{\sqrt{5}-1}{2}b,$

therefore, the equilibrium is

$\left(Qx^*, QT^*_{0,1}, QT^*_{0,2}\right) = \left(b, \frac{\sqrt{5}-1}{2}b, (1 - \frac{\sqrt{5}-1}{2})b\right).$

Since $\frac{d}{dx}b = 0$ (with respect to any independent variable x) and

$\frac{d}{dx}\left(\frac{b^2}{b+x}\right) = -\frac{b^2}{(b+x)^2},$

therefore, the Jacobian of system (B.1)-(B.3) is

$$J(Qx, QT_{0,1}, QT_{0,2}) = \begin{bmatrix} 0 & 0 & 0 \\ 0 & -\frac{b^2}{(b+QT_{0,1})^2} & 0 \\ 0 & -1 & 0 \end{bmatrix},$$

which, when considered at the equilibrium, takes the following simplified form:

$$\begin{bmatrix} 0 & 0 & 0 \\ 0 & -\frac{4}{(1+\sqrt{5})^2} & 0 \\ 0 & -1 & 0 \end{bmatrix}.$$

The eigenvalues of the latter matrix are $\lambda_1 = \lambda_2 = 0$, and $\lambda_3 = -\frac{4}{(1+\sqrt{5})^2}$. Since the absolute values of all the eigenvalues are less than 1, then according to the well-known stability analysis for discrete-time systems (see e.g. (Elaydi, 2005)), the obtained equilibrium $(Qx^*, QT_{0,1}^*, QT_{0,2}^*) = \left(b, \frac{\sqrt{5}-1}{2}b, (1 - \frac{\sqrt{5}-1}{2})b\right)$ is locally asymptotically stable with respect to the subspace of variables Qx, $QT_{0,1}$ and $QT_{0,2}$.
